# A highly photostable and bright green fluorescent protein

**DOI:** 10.1038/s41587-022-01278-2

**Published:** 2022-04-25

**Authors:** Masahiko Hirano, Ryoko Ando, Satoshi Shimozono, Mayu Sugiyama, Noriyo Takeda, Hiroshi Kurokawa, Ryusaku Deguchi, Kazuki Endo, Kei Haga, Reiko Takai-Todaka, Shunsuke Inaura, Yuta Matsumura, Hiroshi Hama, Yasushi Okada, Takahiro Fujiwara, Takuya Morimoto, Kazuhiko Katayama, Atsushi Miyawaki

**Affiliations:** 1 Biotechnological Optics Research Team, RIKEN Center for Advanced Photonics, Saitama, Japan; 2 Laboratory for Cell Function Dynamics, RIKEN Center for Brain Science, Saitama, Japan; 3 Asamushi Research Center for Marine Biology, Tohoku University, Aomori, Japan; 4 Department of Biology, Miyagi University of Education, Sendai, Japan; 5 Department of Infection Control and Immunology, Ōmura Satoshi Memorial Institute, Kitasato University, Tokyo, Japan; 6 Safety Science Laboratories, Kao Corporation, Tokyo, Japan; 7 Laboratory for Cell Polarity Regulation, RIKEN Center for Biosystems Dynamics Research, Osaka, Japan; 8 Department of Cell Biology and Department of Physics, UBI and WPI-IRCN, The University of Tokyo, Tokyo, Japan; 9 Institute for Integrated Cell-Material Sciences, Kyoto University, Kyoto, Japan; 10 Present Address: Graduate School of Integrated Sciences for Life, Hiroshima University, Hiroshima, Japan; 11 Present Address: Narita Elementary School, Miyagi, Japan

**Keywords:** Fluorescence imaging, Fluorescent proteins, Wide-field fluorescence microscopy, Super-resolution microscopy, Super-resolution microscopy

## Abstract

The low photostability of fluorescent proteins is a limiting factor in many applications of fluorescence microscopy. Here we present StayGold, a green fluorescent protein (GFP) derived from the jellyfish *Cytaeis uchidae*. StayGold is over one order of magnitude more photostable than any currently available fluorescent protein and has a cellular brightness similar to mNeonGreen. We used StayGold to image the dynamics of the endoplasmic reticulum (ER) with high spatiotemporal resolution over several minutes using structured illumination microscopy (SIM) and observed substantially less photobleaching than with a GFP variant optimized for stability in the ER. Using StayGold fusions and SIM, we also imaged the dynamics of mitochondrial fusion and fission and mapped the viral spike proteins in fixed cells infected with severe acute respiratory syndrome coronavirus 2. As StayGold is a dimer, we created a tandem dimer version that allowed us to observe the dynamics of microtubules and the excitatory post-synaptic density in neurons. StayGold will substantially reduce the limitations imposed by photobleaching, especially in live cell or volumetric imaging.

## Abstract (web summary)

StayGold is over one order of magnitude more photostable than current fluorescent proteins

## Main

Over the past few decades, owing to improvements in gene transfer and protein-targeting techniques, researchers have succeeded in labeling subcellular components, such as organelles and the cytoskeleton, with fluorescent proteins (FPs), enabling efficient observation of these structures in living cells and organisms^1,2^. For most applications, as low as possible expression levels of an FP-tagged protein are preferable to minimize the perturbation of the system under observation^3^. However, obtaining high signal-to-noise-ratio images from samples with low-level expression requires strong excitation light, which often causes severe photobleaching. Similarly, obtaining a high spatiotemporal resolution to study the fast dynamics of fine subcellular structures requires continuous acquisition of images on a time scale from seconds to minutes^4,5^, which inevitably leads to noticeable FP photobleaching. Highly photostable FPs are, therefore, needed to enable fast and long super-resolution imaging.

To date, the development of photostable FPs has nearly always been accompanied by a decrease in brightness^6,7^. Exceptions could be observed in the development of stable and bright blue-emitting FPs, such as Azurite^8^ and EBFP2 (ref. ^9^), and a recently published photostable yellow-emitting FP, mGold^10^. The main complication arises from molecular oxygen (O_2_), which is required for maturation of chromophores of FPs, and high accessibility to O_2_ contributes to the increase in maturation speed and maximal brightness in cells^11^. However, decomposition of FP chromophores also involves O_2_, which reacts in a photochemical reaction with the chromophores that remain in their singlet or triplet excited states^12^. In this regard, enhanced O_2_ accessibility decreases photostability. Accordingly, there is a tradeoff between brightness and photostability in FP performance.

A typical example of this tradeoff can be seen in the evolution of two monomeric orange-emitting FPs, mOrange^13^ and mKusabiraOrange (mKO)^14^, which were developed independently by two research groups. mOrange matures faster but photobleaches more easily than mKO. Subsequently, a variant of mOrange (mOrange2) was developed that achieved photostability at the expense of maturation speed^6^. On the other hand, a fast-maturing variant of mKO (mKO2) was developed at the expense of photostability^15^. In addition, although a large number of bright green-emitting FPs have been developed, most of them are less photostable than enhanced GFP (EGFP). Because of the apparent inverse relationship between photostability and brightness in the current generation of FPs, few substantial gains in designing bright and photostable products have been made.

Here we report the cloning of an FP with exceptional photostability from the jellyfish *C. uchidae*. In contrast to most previous engineered FPs, introduction of a single point mutation substantially increased the brightness of the FP without compromising the photostability. The resulting protein, called StayGold, can be localized to subcellular components with appropriate tags. To fully benefit from the rich photon budget, we combined StayGold with super-resolution SIM. In live cell imaging experiments, we visualized the ER and mitochondria with improved spatiotemporal resolution and a considerable extension of the observation period. In fixed samples, on the other hand, we could increase signal intensity substantially by using intense illumination powers for excitation. We show that StayGold can be molecularly linked to a single-domain antibody fragment^16^ to enable super-resolution immunocytochemical detection of severe acute respiratory syndrome coronavirus 2 (SARS-CoV-2) spike protein.

## Results

### Molecular cloning and mutagenesis of FP from jellyfish

**Fig. 1:**
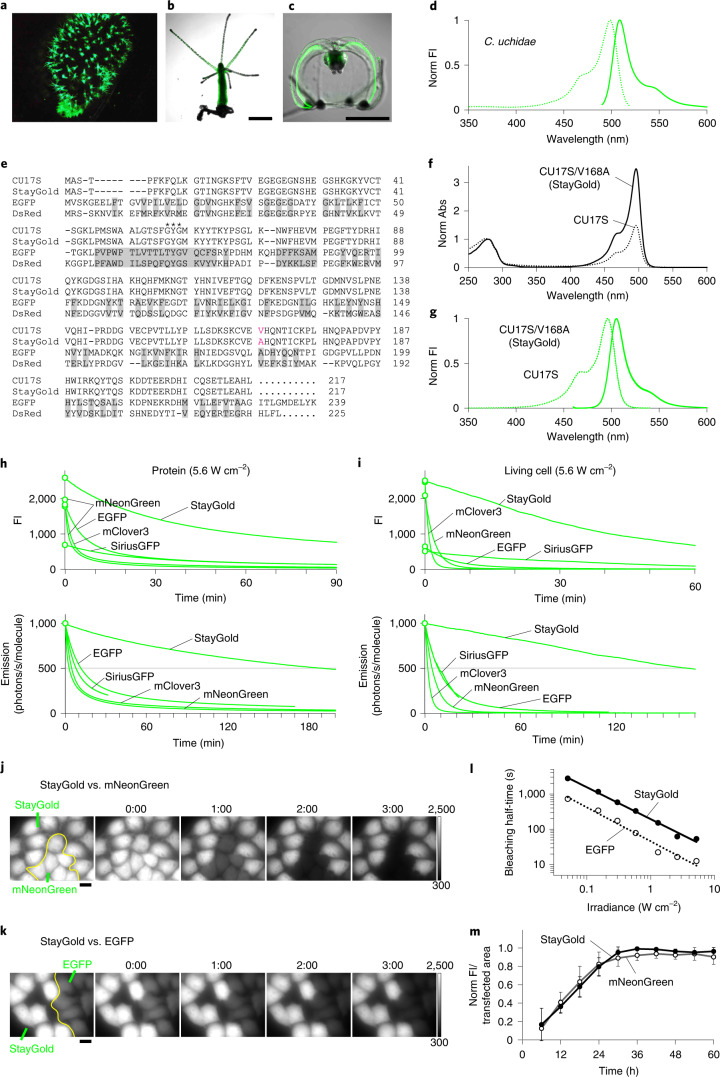
Photostable properties of jellyfish-derived fluorescent protein StayGold. **a**–**c**, Natural fluorescence of *C. uchidae*. Fluorescent polyps on a shell of *N. livescens*; the shell is ellipsoidal and measures approximately 1.5 cm by 1.0 cm (**a**). Fluorescence images of an isolated polyp (**b**) and a female medusa (**c**) superimposed on differential interference contrast images. Scale bars, 0.5 mm. **d**, Normalized excitation (dotted line) and emission (solid line) spectra of a tissue homogenate prepared from *C. uchidae* medusae. **e**, Amino acid sequence alignments of CU17S, StayGold, EGFP and DsRed. Residues whose side chains form the interior of the β-barrel are shaded (EGFP and DsRed). Asterisks: residues responsible for chromophore synthesis. The V168A mutation is indicated in red. **f**, Absorption spectra of CU17S (dotted line) and StayGold (solid line), normalized against the peak at 280 nm. **g**, Normalized excitation (dotted line) and emission (solid line) spectra of CU17S and StayGold. **h**, **i**, Photostability of green-emitting FPs under continuous WF illumination (5.6 W cm^−^^2^). Plotted as measured intensity versus time (top) or as intensity versus normalized total exposure time with an initial emission rate of 1,000 photons/s/molecule (bottom). **h**, Purified protein (1 μM) in polyacrylamide gel. The data are shared with Fig. 2b. **i**, Expressed in human (HeLa) cells in HBSS. **j**, **k**, Fluorescence images of StayGold-expressing and mNeonGreen-expressing (**j**) or EGFP-expressing (**k**) cells (stable HeLa cell transformants) at the indicated times (minutes:seconds). Illumination intensity: 16 W cm^−^^2^. The gray scale indicates the lowest and highest intensities of the image. Scale bars, 20 μm. See Supplementary Video 1. **l**, log–log plot of bleaching half-time (*Y*) of StayGold (solid circles) or EGFP (open circles) and irradiance (*X*). Data were fitted to the equation log(*Y*) = −αlog(*X*) + *c*. α values were 0.90 and 0.96 for StayGold and EGFP, respectively. **m**, Chromophore maturation of StayGold (solid circles) and mNeonGreen (open circles) after cDNA transfection. Fluorescence intensity divided by the transfected cell occupation area was plotted every 6 hours and normalized to the maximum value. Data points are shown as means ± s.e.m. (*n* = 4 different experiments).

*C. uchidae* (phylum Cnidaria, class Hydrozoa) produces colonies of fluorescent polyps (Fig. 1a,b) on shells of *Nassarius livescens*, a gastropod living in the sandy-mud bottom of the sea^17^. It also produces millimeter-sized free-swimming medusae that express green fluorescence in the epithelium of the ex-umbrella and the sub-umbrella, as well as in the gonads (Fig. 1c). This jellyfish has been used as an educational material to help school children in Sendai City, Japan, learn about fluorescence (Supplementary Fig. 1a). Although medusa fluorescence has been spectroscopically characterized (Fig. 1d), its molecular basis remains unknown.

Our RNA sequencing (RNA-seq) and subsequent anchored polymerase chain reaction (PCR) analyses using total RNA from *C. uchidae* (Supplementary Fig. 1b) identified a transcript encoding an FP with a chromophore-forming tripeptide GYG at the appropriate position. This protein, temporarily referred to as CU17S, had a unique primary structure (Supplementary Fig. 1c). The closest homologue, sharing only 15.2% identity, was obeCFP, an FP cloned from *Obelia* medusa^18^. Amino acid sequence alignment of CU17S with EGFP and DsRed is shown in Fig. 1e. Transformation of the cDNA into *Escherichia coli* generated dim green fluorescent colonies. The absorption spectrum of purified CU17S exhibited a major peak at 496 nm (Fig. 1f). The excitation and emission spectra (Fig. 1g) were identical to those observed in *C. uchidae*. When expressed in HeLa cells, the green fluorescence of CU17S was distributed throughout the cytosolic and nuclear compartments. Our qualitative observation by wide-field (WF) microscopy suggested that, although CU17S did not fluoresce brightly in *E. coli* or mammalian cells, the fluorescence did not photobleach substantially. However, because it is generally assumed that there is an inverse correlation between the brightness and photostability of FPs, CU17S was not expected to improve brightness while maintaining excellent photostability. Nevertheless, through random mutagenesis of the CU17S gene, we discovered that the V168A mutation effectively improved the efficiency of both protein expression and chromophore maturation of the FP; CU17S/V168A was abundantly produced in bacteria. Analytical equilibrium ultracentrifugation and pseudonative SDS-PAGE revealed that this FP formed an obligate dimer (Supplementary Fig. 2). The absorption spectrum of CU17S/V168A exhibited a lofty peak at 496 nm (Fig. 1f) with a p*K*_a_ of <4 (Supplementary Fig. 3), and the absolute extinction coefficient^19^ (Supplementary Fig. 4a) was 159,000 M^−1^ cm^−1^ at neutral pH. CU17S/V168A had the same excitation and emission spectra as CU17S (Fig. 1g), and the fluorescence quantum yield (QY_f_) was 0.93. These values indicate that the molecular brightness of CU17S/V168A surpasses the most popular *Aequorea* GFP variant EGFP or other bright green-emitting FPs (Table 1). Furthermore, we noted that CU17S/V168A possesses outstanding photostability, which we thought justified its being called ‘StayGold’.

**Table 1:** Characteristics of StayGold and reference green-emitting FPs

Protein	λab^a^/λem^b^ (nm)	ε^c^ (10^3^ M^−1^ cm^−1^)		QY_f_^d^	Brightness		Photostability *t*_1/2_ (s)^g^			Protein yield^h^ (mg L^−1^)
							Protein	Expressed in living cells		
		λab	488		Mol^e^	Cell^f^		HBSS	DMEM	
StayGold	496/505	159	105	0.93	148	2.06	11,487	9,919	12,421	194
EGFP	488/509	51	51	0.71	36	1.00	700	493	481	93
SiriusGFP	502/516	54	35	0.19	10	0.45	477	522	558	127
mClover3	505/518	99	52	0.84	83	1.73	289	116	66	137
mNeonGreen	505/518	112	64	0.87	97	2.05	176	265	335	172

^a^Absorbance maximum. ^b^Emission maximum. ^c^Absolute extinction coefficient at λab (left) and 488 nm (right). The measurement was based on the fact that, after alkali denaturation of these FPs, the chromophore, containing a dehydrotyrosine residue conjugated to the imidazolone group, absorbs light maximally at 447 nm with a molar extinction coefficient of 44,000 M^−1^ cm^−1^ (ref. ^19^). See Supplementary Fig. 4a. ^d^ Fluorescence quantum yield measured using an absolute photoluminescence quantum yield spectrometer. ^e^Product of ε (λab) and QY_f_. This value reflects the molecular brightness of an FP. ^f^Cellular brightness calculated from data shown in Supplementary Fig. 7b. The fluorescence from each green-emitting FP (with excitation at 488 nm) was corrected by mCherry fluorescence and then normalized to that of EGFP (ref. ^43^). Equimolar co-expression of a green-emitting FP and mCherry using the bicistronic expression system^44^. ^g^Time in seconds to reduce emission rate from 1,000 to 500 photons/s/molecule under WF illumination. ^h^Amount of purified protein from 1 L of bacterial culture. All values were measured in this study. SiriusGFP is a variant of EGFP that exhibits a two-fold increase in photostability relative to EGFP but a three-fold decrease in brightness^20^.

### Outstanding photostability of StayGold

We compared the photostability of StayGold with that of four green-emitting FPs: EGFP (ref. ^11^), SiriusGFP (ref. ^20^), mNeonGreen (ref. ^21^) and mClover3 (ref. ^22^). These five FPs were treated under the same conditions in parallel. First, the proteins were expressed in *E. coli* and purified. Based on the protein concentrations determined with a Bradford Assay Kit, we prepared 1 μM FP solutions in polyacrylamide gel. The solution/gel mixtures, sandwiched between two coverslips, were subjected to photobleaching and imaging experiments with continuous unattenuated arc-lamp illumination (5.6 W cm^−^^2^). This simple comparison revealed that StayGold was brighter and strikingly more photostable than any of the other FPs (Fig. 1h, top). We adopted the currently accepted standard method for evaluating FP photostability^6^. First, the extinction coefficients (Supplementary Fig. 4a) at the center wavelength of the illumination (488 nm) and the fluorescence quantum yields of the other FPs were determined (Table 1). Then, consideration of the irradiance (5.6 W cm^−^^2^) and the determinants of brightness of the five FPs yielded the normalized curves for their photobleaching (Fig. 1h, bottom). For each FP, the time for photobleaching from an initial emission rate of 1,000 photons/s/molecule down to 500 (*t*_1/2_) was calculated (Table 1). Of the four previously reported FPs, EGFP had the highest *t*_1/2_ value at 701 seconds. However, the *t*_1/2_ of StayGold far exceeded this value, surpassing 10,000 seconds. These results indicate that StayGold can emit over ten-fold more photons before photobleaching than the other green-emitting FPs.

**Fig. 2:**
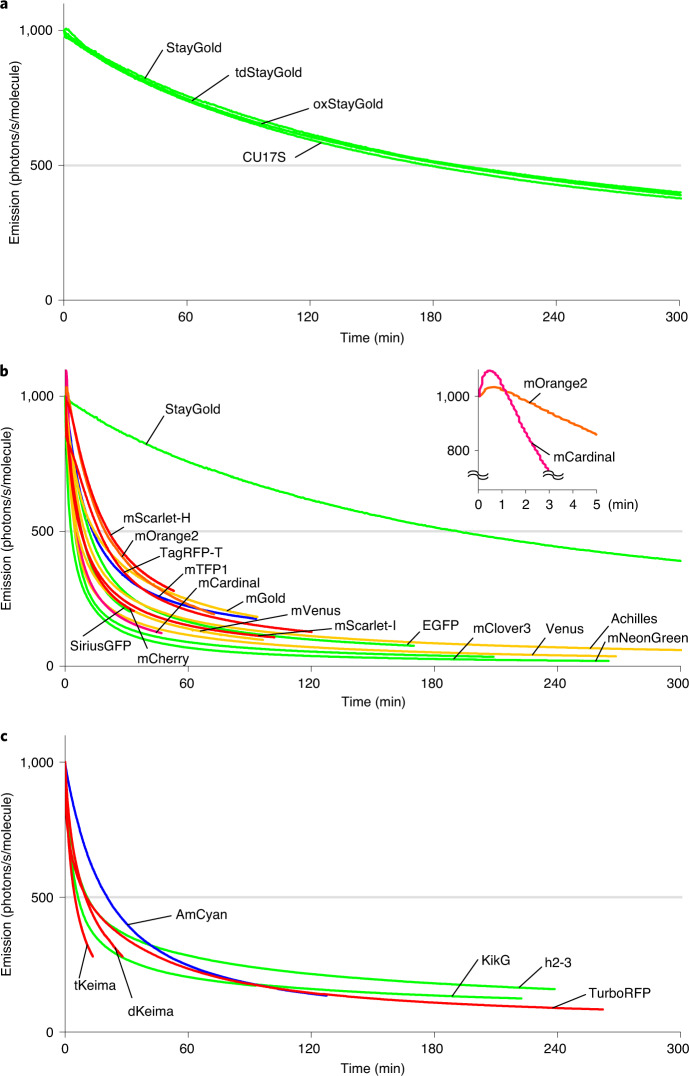
Comparison of photobleaching curves. **a**–**c**, Plotted as intensity versus normalized total exposure time, with an initial emission rate of 1,000 photons/s/molecule. See Supplementary Table 1. **a**, StayGold variants were photobleached under continuous WF illumination (5.6 W cm^−2^). **b**, Various colored FPs were photobleached under continuous WF illumination (3.4–5.6 W cm^−2^). Inset: A photoactivation component was noted for mOrange2 and mCardinal. All curves shown in Fig. 1h (top) are incorporated here. **c**, Multimeric FPs were photobleached under continuous WF illumination (3.4–5.6 W cm^−2^). The curve of StayGold shown in Fig. 1h is incorporated in **a** and **b**.

We examined the brightness-adjusted photostability of fluorescence from living cultured HeLa cells that stably expressed each of the five FPs (Fig. 1i and Supplementary Fig. 5). The conspicuous photostability of StayGold was again apparent. For example, whereas the *t*_1/2_ of StayGold was greater than 10,000 seconds, that of EGFP was below 500 seconds (Table 1). In one attempt, we mixed StayGold-expressing cells and mNeonGreen-expressing cells for direct comparison of photobleaching in a single field of view and verified the large difference in their photobleaching rates (Fig. 1j and Supplementary Video 1, first half). It should be noted that the two cell populations were indistinguishable at *t* = 0 because they had the same initial fluorescence intensities. We also performed a one-to-one comparison between StayGold-expressing and EGFP-expressing cell lines and found that the former was brighter and more photostable than the latter (Fig. 1k and Supplementary Video 1, 2nd half). The superior photostability of StayGold relative to EGFP was observed across the full range of light intensities of the arc-lamp illumination (Fig. 1l). To examine the involvement of O_2_ in the photobleaching of the five green-emitting FPs, we exposed FP-expressing living cells to continuous arc-lamp illumination under anoxic and hyperoxic conditions, in addition to normoxic conditions, and found that StayGold was O_2_-sensitive, as were the other FPs (Supplementary Fig. 6). We then performed time-lapse imaging experiments after cDNA transfection into HeLa cells. Although StayGold showed stronger fluorescence than mNeonGreen half a day or 3 days after transfection (Supplementary Fig. 7a), their maturation rates proved to be the same (Fig. 1m). Quantification of the cellular brightness of the five green-emitting FPs verified the most efficient maturation of both StayGold and mNeonGreen (Supplementary Fig. 7b and Table 1). Compared to StayGold, CU17S is poorly folded in both bacterial and mammalian expression systems (Supplementary Fig. 4b). It is noted, however, that folded CU17S is as photostable as StayGold (Fig. 2a and Supplementary Table 1a).

Over the past 2 decades, photostability has been improved for red-, orange- and cyan-emitting FPs to develop several robust FPs, such as TagRFP-T (ref. ^6^), mOrange2 (ref. ^6^), mScarlet-H (ref. ^7^) and mTFP1 (ref. ^23^). Although a direct comparison of FPs of different colors is impossible, it is possible to quantitively assess their photostability on the basis of *t*_1/2_ calculations (Supplementary Fig. 4c). Figure 2b and Supplementary Table 1b show the normalized photobleaching data for 16 FPs that we investigated; the findings demonstrate the superiority of StayGold’s glowing capability within the realm of FPs.

### Labeling ER lumen with StayGold-based markers

We expressed StayGold and other green-emitting FPs as soluble markers of the ER lumen by fusing the calreticulin signal sequence and the ER-retrieval motif to their N- and C-termini, respectively (er-FPs). In our efforts to direct StayGold to the ER lumen, however, we faced two technical challenges.

First, cysteine residues in an FP prevent correct folding of the β-barrel due to the formation of unnecessary disulfide bonds in the oxidizing ER environment. To date, cysteine-less variants of FPs (oxFPs), including oxGFP, have been successfully engineered and used to efficiently label the ER from the inside^1^. StayGold has five cysteine residues. After site-directed random mutagenesis, we generated oxStayGold, in which Cys174 and Cys208 are replaced with Ile. Photobleaching experiments using purified proteins showed that these two mutations did not affect the outstanding photostability of StayGold (Fig. 2a and Supplementary Table 1a).

Second, StayGold’s head and tail are relatively short (Fig. 1e), which makes this FP sensitive to C- and N-terminal fusions. We borrowed termini from other proven FPs and found that nine amino acids from the N-terminal region of EGFP (n1 or n2) and ten amino acids at the C-terminus of dfGFP (ref. ^24^) (c4) could be fused to StayGold (Supplementary Fig. 8) to improve its targeting function to some subcellular components (Supplementary Fig. 9).

**Fig. 3:**
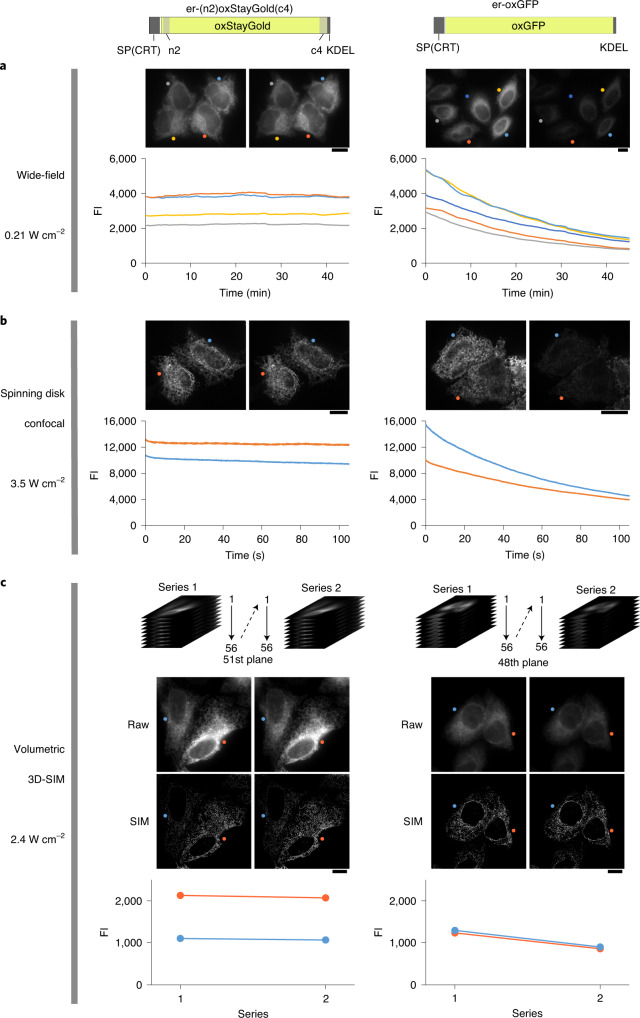
Photostability of a cysteine mutant of StayGold targeted to the ER lumen. HeLa cells expressing er-(n2)oxStayGold(c4) or er-oxGFP were subjected to continuous live imaging. Comparison was made side by side. Scale bars, 10 μm. **a**, WF (arc-lamp) illumination with an irradiance value of 0.21 W cm^−2^. **b**, Spinning disk confocal illumination with an irradiance value of 3.5 W cm^−2^. **a**,**b**, The first and last images are shown (top). The averaged fluorescence intensities of individual cells are plotted against time (bottom). **c**, Volumetric 3D-SIM imaging with an irradiance value of 2.4 W cm^−2^. Repetitive collection of a 3D stack of 56 3D-SIM images. Raw and reconstructed 3D-SIM images of the 51st (left) or 48th (right) plane in *z*-series 1 and 2. The averaged fluorescence intensities of individual cells are plotted (bottom).

Ultimately, we generated a construct, er-(n2)oxStayGold(c4) (Fig. 3), which was targeted to the ER more efficiently than er-oxStayGold or er-(n2)StayGold. WF (Fig. 3a) and spinning disk confocal (Fig. 3b) microscopy confirmed that er-(n2)oxStayGold(c4) was more tolerant to continuous illumination than er-oxGFP.

### Analysis of ER network rearrangements

The ER is organized into an interconnected reticular network of branching tubules that extends throughout the entire cytosol. Although previous studies mostly imaged only a part of the ER network (the peripheral ER), such local imaging is not appropriate for the assessment of photobleaching in the ER because diffusional exchange occurs between bleached and unbleached FPs. Therefore, we always placed the entire cell within the field of view. For fast and cell-wide super-resolution imaging of the ER network, we employed three-dimensional structured illumination microscopy (3D-SIM), a WF microscopy technique that demands great photostability of dyes because it acquires and combines 15 (five phases × three angles) images per frame^25^. Unlike multiphoton and light sheet microscopy, 3D-SIM excites fluorophores both above and below the focal plane while enabling optical sectioning (Supplementary Fig. 10). Accordingly, while a *z*-series is collected for volumetric visualization of a specimen, each fluorophore is almost continuously exposed to excitation light. We performed comparative volumetric 3D-SIM imaging experiments and observed substantial photobleaching of er-oxGFP, but not er-(n2)oxStayGold(c4), after acquisition of a *z*-series stack (Fig. 3c).

**Extended Data Fig. 1:**
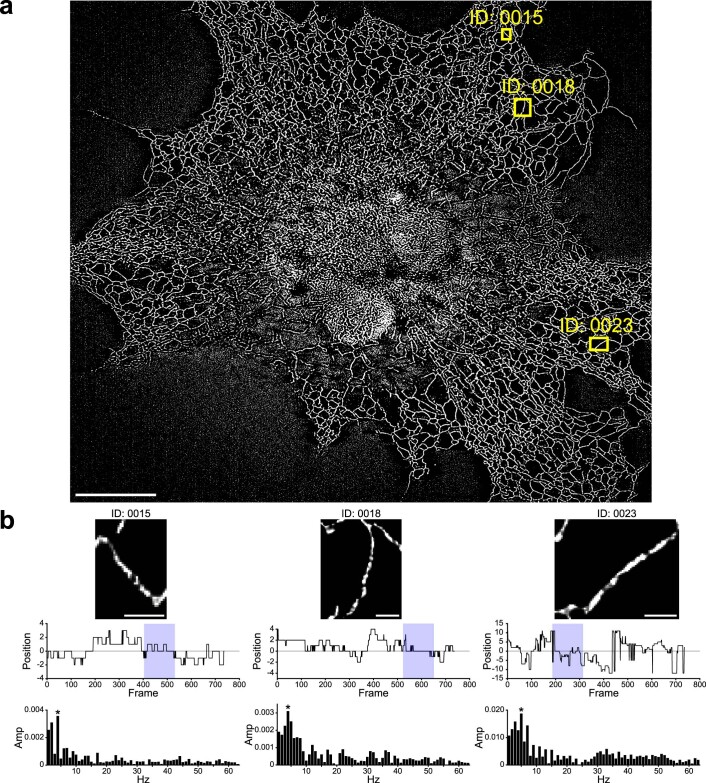
Analyzing rapid motion of ER tubules by using a new 3D-SIM technique that achieves nanoscale resolution on a millisecond time scale. A COS-7 cell expressing er-(n2)oxStayGold(c4) was imaged by lattice SIM continuously at the temporal resolution of 134.47 frames/s for 5.473 s. The total number of frames was 736. See Supplementary Video 2. **a**, A binarized image. Scale bar, 10 μm. **b**, Oscillating ER tubules with relatively stable anchors were selected. Shown are three examples observed in boxed regions (ID: 0015, ID: 0018, and ID: 0023). *top*, High-magnification images. Scale bars, 1 μm. *middle*, Kymograph built along lines drawn perpendicularly to selected tubules. *bottom*, Fast Fourier transform (FFT) in a time window consisting of 128 (2^7^) consecutive time points (shaded zone). FFT spectra with a frequency unit of 1.05 (134.47/128) Hz and the Nyquist frequency (highest frequency to be analyzed) of 67.235 (134.47/2) Hz. In the selected peripheral ER, FFT spectra peaking at 4.2 and 5.25 Hz (asterisks) were detected. Shown is a representative of n = 5 independent experiments that imaged rapid motion of ER tubules at the temporal resolution of > 100 frames/s.

Two recent studies elegantly visualized the peripheral ER network using grazing incidence (GI)-SIM, a new two-dimensional (2D)-SIM technique that creates an appropriately thick illumination field over a cover glass (Supplementary Fig. 10). This technique can image the entire volume of the basal cell cortex for more comprehensive analysis of the ER than total internal reflection fluorescence structured illumination microscopy (TIRF-SIM). Owing to the higher fluorescence intensity, GI-SIM achieves higher temporal resolution than TIRF-SIM. Using GI-SIM, Nixon-Abell et al. and Guo et al. imaged the ER near the basal plasma membrane at 40 and 266 frames per second, respectively, at a ~100-nm resolution, to discover that the so-called ‘peripheral ER sheets’ are dynamic, dense ER tubular arrays that oscillate very rapidly (4–10.1 Hz)^4,5^. We confirmed these findings by employing an advanced 3D-SIM system with a cell-wide field of view that covered the perinuclear as well as peripheral regions. The entire tubular network was visualized at a temporal resolution of 134.47 frames per second (Extended Data Fig. 1 and Supplementary Video 2).

**Fig. 4:**
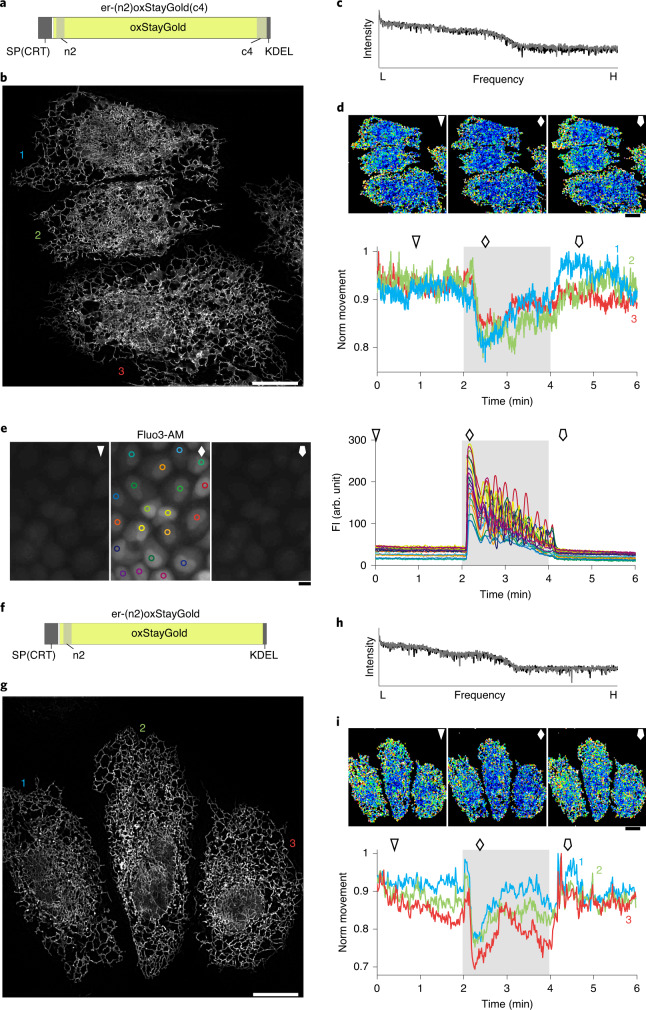
Agonist-induced and antagonist-induced longitudinal changes in ER structures revealed by fast, sustainable, wide 3D-SIM. **a**–**d**, Three neighboring HeLa cells expressing er-(n2)oxStayGold(c4) were imaged continuously at a temporal resolution of 2.6 frames per second. Illumination intensity: 2.4 W cm^−2^. See Supplementary Video 3. **a**, Domain structure of er-(n2)oxStayGold(c4). SP(CRT), calreticulin signal peptide. **b**, A 3D-SIM image at one time point. **c**, FFT spectra of the first and last images (gray and black lines, respectively) from the reconstructed dataset. **d**, Quantification of ER network rearrangements. Temporal profiles of ER movement in individual cells are shown. **e**, In a separate experiment, Fluo3 was used to measure intracellular free Ca^2+^ concentration ([Ca^2+^]_i_) in cultured HeLa cells. In all observed cells, the application of histamine (10 μM) resulted in an initial peak and subsequent sinusoidal oscillations in [Ca^2+^]_i_; the additional application of cyproheptadine (100 μM) stopped the oscillations, resulting in a drop in [Ca^2+^]_i_ to previous resting values. **f**–**i**, Three neighboring HeLa cells expressing er-(n2)oxStayGold were imaged continuously at 1.1 frames per second. Illumination intensity: 2.4 W cm^−2^. See Supplementary Video 4. **f**, Domain structure of er-(n2)oxStayGold. **g**, A 3D-SIM image at one time point. **h**, FFT spectra for the first and last images (gray and black lines, respectively) of the reconstructed dataset. **i**, Quantification of ER network rearrangements. Temporal profiles of ER movement in individual cells. **d**,**e**,**i**, 10 μM histamine and 100 μM cyproheptadine were applied at 2 minutes and 4 minutes, respectively; the time zone of Ca^2+^ mobilization is shaded. Movement heat maps (**d** and **i**) or fluorescence images (**e**) at time points indicated by triangles, rhombuses and pentagons are shown. **b**,**g**, ER labeling shown in **g** was 8–9 times dimmer than that in **b**.**c**,**h**, L: low; H: high. Scale bars in **b**,**d**,**e**,**g** and **i**, 10 μm.

**Extended Data Fig. 2:**
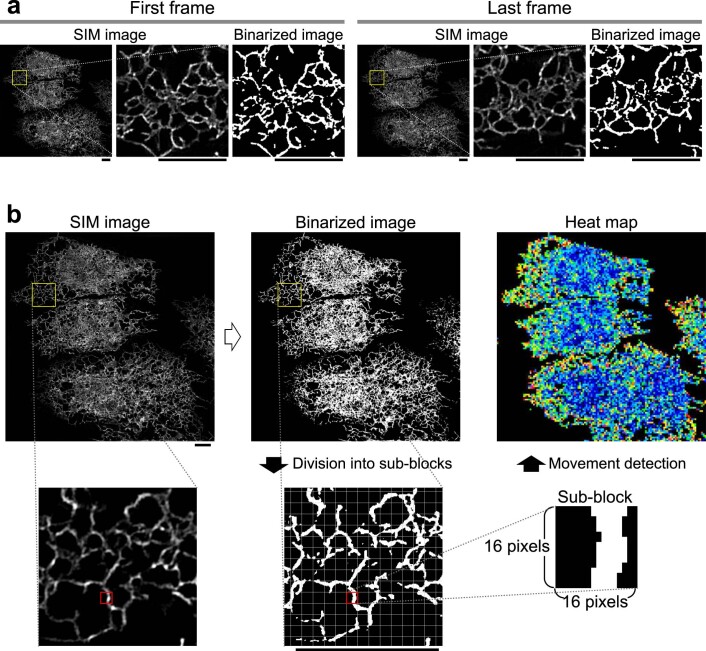
Cell-wide, spatiotemporally high-resolution, sustainable imaging of ER network rearrangement. The three neighboring HeLa cells expressing er-(n2)oxStayGold(c4) (Fig. 4a–d) were analyzed. Scale bars, 5 μm. **a**, Magnified SIM and binarized images at t = 0 min (First frame) and t = 6 min (Last frame) of a peripheral region of the uppermost cell. Compare with Supplementary Fig. 11i. The preservation of 3D-SIM image quality in the last frame was confirmed in all six-minute experiments that employed er-(n2)oxStayGold(c4). Shown is a representative of n = 9 independent transfections, including experiments #1, #2, #3, and #4 (Supplementary Fig. 11a-d). **b**, Automatic quantification of ER network rearrangement. See Methods (Automatic quantification of ER network rearrangement). Magnifications of the boxed region are shown (bottom). Considering that the diameter of an ER tubule spanned several pixels, we reasoned that the sub-block size was optimal for the efficient detection of target movement.

To thoroughly investigate multiple mechanisms of tubular ER formation, Guo et al. also used GI-SIM to observe ER network rearrangements with a temporal resolution of 0.5 Hz^5^. By contrast, we acquired single-layer 3D-SIM images of three neighboring HeLa cells expressing er-(n2)oxStayGold(c4) (Fig. 4a,b) at 2.6 Hz continuously over 6 minutes, during which the cells were challenged with a histamine and an anti-histamine reagent sequentially to initiate Ca^2+^ mobilization and then shut it down (Supplementary Video 3). As evidenced by fast Fourier transform (FFT) spectral data^26^ (Fig. 4c), the super-resolution quality of the 3D-SIM images was well preserved throughout the experiment (Extended Data Fig. 2a). To comprehensively characterize ER network dynamics, we performed fully automatic image processing (Extended Data Fig. 2b). After extraction of fluorescent ER tubules, a 2K image was divided into sub-blocks, each of which consisted of 16 × 16 pixels. We used a custom-made algorithm to quantify the displacement of ER tubules in each sub-block. As shown by the heat maps (Fig. 4d, top), the ER network was most mobile near the plasma membrane at the free edge. Interestingly, the overall ER network was rather immobile (Fig. 4d, bottom) while inositol 1,4,5-trisphosphate-induced Ca^2+^ release took place (Fig. 4e). After conducting separate experiments using er-(n2)oxStayGold(c4) and er-oxGFP and analyzing the data comparatively, we confirmed that high photostability is essential to this type of ER imaging by 3D-SIM (Supplementary Fig. 11 and Supplementary Discussion 1).

We also generated another construct, er-(n2)oxStayGold (Fig. 4f,g), which appeared to label ER more moderately than er-(n2)oxStayGold(c4). The reversible, Ca^2+^ mobilization-dependent stabilization of ER network dynamics was verified in a sustainable 3D-SIM experiment that visualized er-(n2)oxStayGold-accumulating ER tubules at 1.1 Hz (Fig. 4h,i and Supplementary Video 4).

### Labeling mitochondrial matrix with StayGold-based markers

We engineered StayGold and mNeonGreen to localize in the mitochondrial matrix by fusing a tandem repeat of the COX VIII pre-sequence^27^ (Supplementary Fig. 12a; mt-StayGold and mt-mNeonGreen). When transfected into HeLa cells, substantial cell-to-cell variation in the expression level was observed for each construct. Among mt-StayGold transfectants, we chose a rather dim cell with modestly labeled mitochondria for continuous 3D-SIM imaging. However, due to the high motility of mitochondria, we had to increase the imaging frame rate and, accordingly, the illumination intensity to achieve sufficient spatial resolution. mt-StayGold provided moderate but well-sustained signals throughout an experiment (Supplementary Video 5), whereas mt-mNeonGreen lost its initial bright signals halfway under the same conditions.

**Extended Data Fig. 3:**
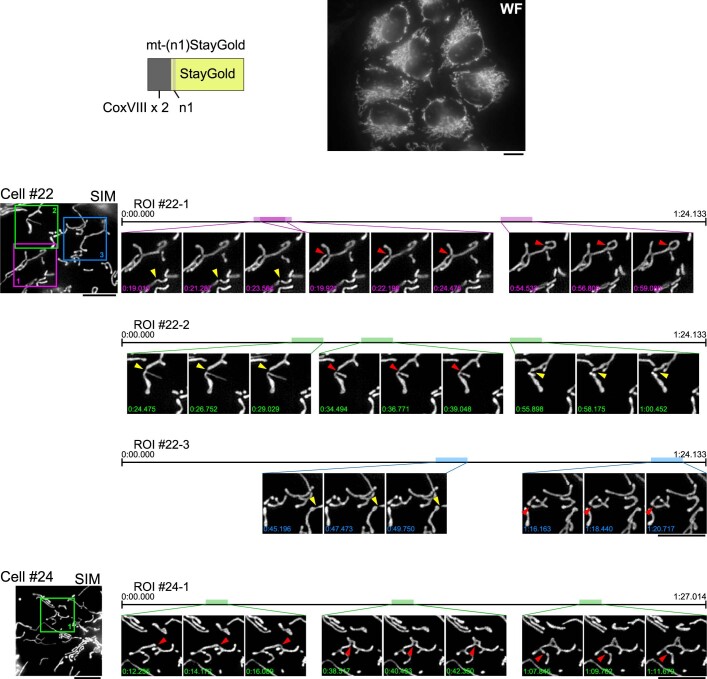
Visualizing mitochondrial fission and fusion by StayGold SIM technology. *upper*, HeLa cells stably expressing mt-(n1)StayGold were photographed by wide-field (WF) microscopy. The uniformity of fluorescence intensity among observed cells of this cell line is evident. *lower*, HeLa cells stably expressing mt-(n1)StayGold were imaged by 3D-SIM continuously with an irradiance value of 3.9 W/cm^2^. Shown are two representative experimental results. In one experiment, which spanned 1 min 24.133 sec, Cell #22 was imaged at 4.4 Hz. In the other experiment, which spanned 1 min 27.014 sec, Cell #24 was imaged at 5.3 Hz. SIM images in boxed regions (ROIs) at the indicated times (min: sec) are presented to show fission (yellow arrowhead) and fusion (red arrowhead) events detected during the observation time periods. Each event is highlighted by a triad of images. Scale bars, 5 μm. Similar results were obtained from 2 other independent cultured cell samples.

Although we chose cells with moderate FP expression levels for live imaging, it was also interesting to what extent mitochondrial labeling intensity could be increased with the soluble markers. We noticed that mt-(n1)StayGold gave much brighter mitochondrial labeling than mt-StayGold (Supplementary Fig. 12b and Supplementary Discussion 2). We constructed HeLa cells that constitutively expressed mt-(n1)StayGold. The stable transformant proliferated normally (Supplementary Fig. 5b) while exhibiting strong and uniform labeling of mitochondria, enabling fast, sustained, wide 3D-SIM imaging for efficient detection of their fission and fusion (Extended Data Fig. 3). As these events take place under a variety of metabolic stress conditions^28^, high-resolution imaging of live mitochondria should also be cell-wide and long-term for a comprehensive understanding of their regulations.

### StayGold tandem dimer

For protein fusion applications, we attempted to fuse two copies of a StayGold construct that was appended at both the N- and C-termini using a flexible linker (EV linker)^29^ to create a tandem dimer (Supplementary Fig. 13a). The resultant FP construct, tdStayGold, showed the same photostability as StayGold (Fig. 2a, Supplementary Fig. 4b and Supplementary Table 1a). An OSER assay^3^ using CytERM-tdStayGold showed almost no whorl structures, indicating the monovalent fusion of tdStayGold (Supplementary Fig. 13b).

**Extended Data Fig. 4:**
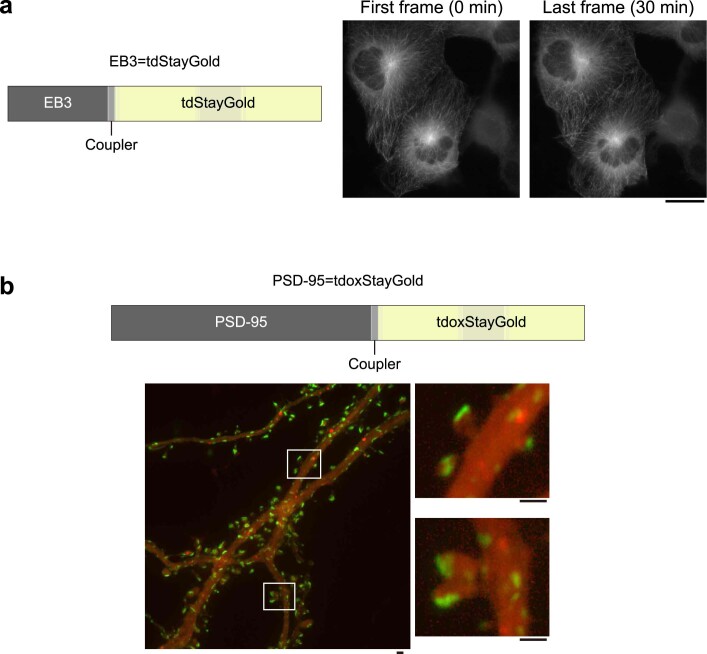
Protein fusion applications. **a**, Labeling microtubule plus-end with tdStayGold. *left*, Domain structure of EB3 = tdStayGold. EB3: Microtubule (MT)-associated end-binding protein 3. *right*, COS-7 cells expressing EB3 = tdStayGold were continuously imaged for 30 min (see Supplementary Video 6). Exposure time: 0.5 s. The first and last fluorescence images are shown. Comparison of the two images reveals that there was no substantial photobleaching during the 30-min observation. Scale bar, 20 μm. The EB3 dynamics shown are representative of n = 3 similar observations. **b**, Labeling postsynaptic density (PSD) with tdoxStayGold. *top*, Domain structure of PSD-95=tdoxStayGold. *bottom*, Cultured neurons prepared from embryonic rat hippocampus were cotransfected with the cDNAs of PSD-95=tdoxStayGold and mCherry at DIV (days in vitro) 5. Transfected neurons were imaged at DIV 25 in DMEM/F12 (1:1) containing 2% (vol/vol) FBS, N2-supplement (1×), and B-27 (1.5×) using the SpinSR10. Volumetric imaging was performed in *z* steps of 0.25 µm. The imaged volume comprised 30 *z* slices. Areas enclosed by white boxes are enlarged on the right side. Scale bars, 1 µm. Similar images were obtained from 6 other imaging experiments performed at DIV 21–25 on 4 independent neuronal samples. **a**,**b**, =: Coupler linker, a triple repeat of the amino acid linker Gly-Gly-Gly-Gly-Ser.

Microtubule (MT)-associated end-binding protein 3 (EB3), a core component of the MT plus-end protein complex, surfs on the growing tips of MTs. As MT dynamics was found to depend on the monomer/dimer equilibrium of EB3 (ref. ^30^), we tagged this protein with tdStayGold (Extended Data Fig. 4a). We imaged COS cells that expressed EB3 = tdStayGold by WF microscopy continuously. We visualized many fluorescent comets moving along MTs toward the cell periphery with minor background (cytosolic) fluorescence (Supplementary Video 6). No photobleaching was noted during the 30-minute observation. As the EB3 dynamics seemed to be preserved until the end of measurements, it is probable that the illumination mode did not cause any substantial phototoxicity.

PSD-95 is an important excitatory post-synaptic density (PSD) protein that regulates the trafficking and localization of glutamate receptors and signaling molecules to modulate synaptic plasticity^31^. As PSD-95 is involved in multivalent interaction networks in PSD (ref. ^32^), the FP used for tagging this protein should be monomeric. We tagged PSD-95 with a similar tandem dimer, tdoxStayGold. We used spinning disk confocal microscopy to image cultured neurons that expressed PSD-95-tdoxStayGold and observed disc-shaped signal assemblies on dendritic spines and shafts (Extended Data Fig. 4b and Supplementary Discussion 3).

### Visualizing SARS-CoV-2 assembly by StayGold/SIM technology

In 2020, the Coronavirus Disease 2019 (COVID-19) pandemic caused by SARS-CoV-2 substantially affected our study aimed at practical application of StayGold. In hopes of combatting the pandemic with resources at hand, we embarked on an urgent project to develop a technique for visualizing SARS-CoV-2 using FPs. This virus expresses a surface spike (S) glycoprotein that is responsible for viral entry into the host cell; the protein ectodomain is composed of the S1 and S2 subunits. Over the past year, several potent neutralizing antibodies were developed against the S1 protein. We were most interested in nanobodies (Nbs), which are the variable domains of heavy chain-only antibodies and can be genetically engineered to be fluorescent antigen detectors via FP fusions^16,33^. Screening for S1 binding ability in a cDNA library of 10 trillion synthetic Nb sequences yielded a high-affinity (1.4 nM) binder. Due to its high neutralization potency, the Nb, hereinafter referred to as Nb(S1), effectively alleviated symptoms in SARS-CoV-2-infected Syrian hamsters after nasal delivery^34^.

**Fig. 5:**
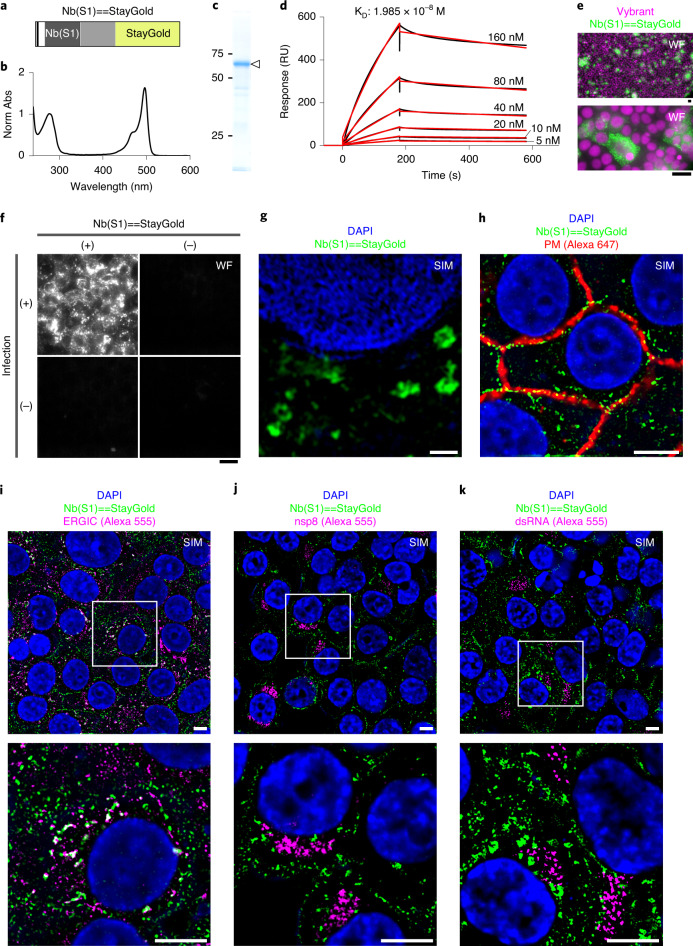
Visualizing SARS-CoV-2 assembly in cells by StayGold SIM technology. **a**, Domain structure of Nb(S1) = = StayGold. = =: EV linker^29^, indicated as a gray bar. **b**, Absorption spectrum of Nb(S1) = = StayGold. Normalized against the peak at 280 nm. **c**, Coomassie brilliant blue staining for the visualization of Nb(S1) = = StayGold separated by SDS-PAGE. The migration position is indicated by an arrowhead. **d**, SPR experiment of S1 binding to immobilized Nb(S1) = = StayGold. Black traces show raw data; red lines show kinetic fit. **e**, VeroE6/TMPRSS2 cells infected with SARS-CoV-2 at an MOI of 0.1 and fixed at 24 hours post-infection (hpi). Spike protein (green). Nuclei were counterstained with Vybrant (magenta). Representative of *n* = 9 independent samples. **f**, SARS-CoV-2-infected (top) and SARS-CoV-2-uninfected (bottom) VeroE6/TMPRSS2 cells. Treated (left) and untreated (right) with Nb(S1) = = StayGold (3.4 μg ml^−1^). Representative of *n* = 2 independent infections. **g**, A single-layer 3D-SIM image of SARS-CoV-2 spike protein (green) and Nuclear (blue) extracted from volumetric image data shown in Extended Data Fig. 5. Representative of *n* = 5 cells over three independent infections. **h**, A single-layer 3D-SIM image of SARS-CoV-2 spike protein (green), Nuclear (blue) and plasma membrane (red). Representative of *n* = 2 areas over two independent infections. **i**–**k**, Single-layer 3D-SIM images of SARS-CoV-2 spike protein (green) and ERGIC3 (magenta) (**i**), nsp8 (magenta) (**j**) or dsRNA (magenta) (**k**). Nuclear (blue) images are sectional but not SIM-reconstructed. Areas enclosed by white boxes are enlarged below. **e**,**f**, WF, wide-field microscopy observation. **f**–**k**, VeroE6/TMPRSS2 cells infected with SARS-CoV-2 (MOI = 0.02, 36 hpi). **i**–**k**, These single-layer 3D-SIM images are part of volumetric imaging data. Representative of *n* = 3 imaging experiments from a single sample per staining condition (Supplementary Fig. 15). **g**,**h**, Nuclear and plasma membrane images are sectional but not SIM-reconstructed. **h**,**i**, Samples are shared. Scale bars in **e** and **f**, 20 μm; **g**, 1 μm; and **h**–**k**, 5 μm. All the cells were fixed.

Through structure-guided design, two recent studies successfully engineered multivalent Nb constructs with extremely high neutralization potency in vitro^35,36^. Similarly, we expected that divalent binding by StayGold fusion would enhance target binding by increasing avidity. We inserted the EV linker between Nb(S1) and StayGold to generate Nb(S1) = = StayGold (Fig. 5a). This approach achieved good yield, soluble expression in bacterial culture and efficient purification of the recombinant chimeric protein. The full maturation of the StayGold chromophore was verified by absorption measurements (Fig. 5b). Furthermore, the molecular integrity of the fusion construct was verified by SDS-PAGE (Fig. 5c). A surface plasmon resonance experiment revealed that Nb(S1) = = StayGold had somewhat lower affinity toward S1 than Nb(S1) (Fig. 5d).

VeroE6 cells were engineered to constitutively express TMPRSS2, which promotes viral entry by cleaving S protein. VeroE6/TMPRSS2 cells were infected with SARS-CoV-2 and fixed and processed for immunoreaction with Nb(S1) = = StayGold, followed by nuclear counterstaining with Vybrant dye. By conventional WF microscopy, we observed islands of infected cells filled with green fluorescent puncta (Fig. 5e). Fluorescent puncta were observed in infected cells reacted with Nb(S1) = = StayGold, but not in uninfected cells or non-reacted infected cells, indicating that the signal was specific to SARS-CoV-2 infection and S1 (Fig. 5f).

**Extended Data Fig. 5:**
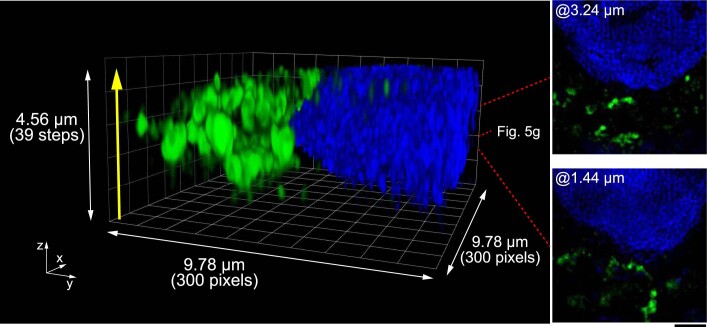
Volumetric 3D-SIM imaging of S protein in a SARS-CoV-2-infected VeroE6/TMPRSS2 cell. The Nb(S1) = = StayGold fluorescence (green) was 3D-SIM reconstructed, whereas DAPI fluorescence (blue) was not. Images were acquired from *z* = 0 to *z* = 4.56 μm in steps of 0.12 μm upward (in the direction of a yellow arrow). A volume of 9.78 × 9.78 × 4.56 μm^3^ is 3D rendered. Two single-layer (*xy*) images at *z* = 1.44 and 3.24 μm are shown. Scale bar, 2 μm. The *z* position of the *xy* image of Fig. 5g is indicated. The volumetric imaging experiment in *z* steps of 0.12 μm was repeated 4 times with 39–79 slices. In each case, the fluorescence in the last frame (slice) was considerably strong for reliable super-resolution imaging.

Like many other RNA viruses, SARS-CoV-2 markedly remodels the endomembrane system of the host cell (Supplementary Fig. 14). The virus-induced, ER-derived membranous structures include convoluted membranes (CMs) and double-membrane vesicles (DMVs). CMs accumulate viral non-structural proteins (nsps) that rearrange host cell membranes to establish DMVs, the major platform for viral replication in double-stranded RNA (dsRNA) species. DMVs and CMs have been well-characterized by a variety of advanced imaging techniques, such as cryo-electron microscopy (cryo-EM)^37–39^. By contrast, although viral assembly takes place mainly in the ER-Golgi intermediate compartment (ERGIC), the assembly process involving the S protein has barely been characterized. A recent study, however, visualized SARS-CoV-2 assembly pathways through a unique combination of cryo-EM techniques:^40^ newly synthesized S proteins are transported in small (~100 nm) dense vesicles, which are fused with ERGIC single-membrane vesicles (SMVs) measuring a few hundred nanometers in diameter, and the interior of these vesicles is lined with S proteins. For the comprehensive high-spatial-resolution mapping of S proteins, we employed volumetric 3D-SIM to resolve punctate signals (Extended Data Fig. 5). The highly photostable StayGold enabled large-scale super-resolution microscopy observations. With the improved spatial resolution of 3D-SIM (100–120 nm), we were able to observe S signals in the shape of a ring in an *x–y* image (Fig. 5g). As S proteins on SMV membranes cluster at the assembly site during viral budding, the ring structure appears to be mostly irregular. Subsequently, assembled virions decorated with S proteins are released into the lumen of SMVs; the virions are transported through the secretory pathway and released by the cell. In fact, we often saw green fluorescence signals on the plasma membrane (Fig. 5h).

To further characterize the S signal-containing vesicles, we performed volumetric dual-color 3D-SIM imaging experiments. ERGIC-SMVs, CMs and DMVs were immunolocalized using anti-ERGIC3, anti-nsp8 and anti-dsRNA antibodies, respectively (Fig. 5i–k and Supplementary Fig. 15); these immunolocalizations were labeled with red fluorescence (Alexa Fluor 555). We observed substantial overlap between S and ERGIC3 signals. By contrast, no overlap was observed between S and nsp8 signals or between S and dsRNA signals.

To confirm the reproducibility of Nb(S1) reactivity, we diversified the construct. We fused Nb(S1) to Achilles, a fast-maturing yellow-emitting FP (ref. ^41^), with another linker to generate Nb(S1) = Achilles (Supplementary Fig. 16). In WF microscopy, very similar punctate fluorescence signals were observed in SARS-CoV-2-infected VeroE6/TMPRSS2 cells. Co-immunostaining of nsp8 with red fluorescence (Alexa Fluor 555) revealed considerable variation in the ratio of S and nsp8 signals among infected cells.

## Discussion

Live cell imaging has proven to be a powerful method to understand the dynamics of various cellular processes in culture and in vivo. However, because of the limited photostability of the current generation of FPs, maintaining a sufficiently high signal-to-noise ratio for extended periods of time required either a high initial brightness of the sample or a low observation frequency. Both strategies have practical limitations. When cellular proteins are labeled with FPs, a common concern is that bright cells with the corresponding high expression levels of the tagged proteins will show non-physiological behaviors, whereas temporal downsampling may miss transient but important signals. The high photostability and brightness of StayGold allowed us to select moderately bright cells for observation with continuous illumination and to perform experiments in which imaging performance was not limited by photobleaching.

StayGold is endowed with outstanding photostability and excellent brightness (for a discussion of the technical difficulties in determining and comparing photostability, see Supplementary Discussion 4). However, a detailed understanding of how StayGold interacts with O_2_ to synthesize its chromophore and why it is so insensitive to photochemical degradation will require determination of the protein structure, which is underway. Also, single-molecule imaging of StayGold, which is also ongoing, will help us to elucidate photophysical properties, including blinking, which is an indicator of inter-system crossing to the triplet state.

We anticipate that er-(n2)oxStayGold(c4) can be effectively combined with methods for rapid super-resolution imaging, such as GI-SIM, to further enhance the maximal duration of imaging experiments. GI-SIM is a specialized 2D-SIM technique that achieves nanoscale resolution on a millisecond time scale. Using GI-SIM, two studies^4,5^ discovered the very rapid oscillatory motion of ER tubules, which could not be characterized by conventional spatiotemporal resolution and explains the appearance of continuous sheet-like structures of the ER. Due to its poor *z*-axis resolution, however, GI-SIM can be applied only to rather loose ER networks in the cell periphery. We employed 3D-SIM instead to image the ER in a cell-wide plane that contained the nucleus. This imaging mode allowed us to detect all changes in the morphology of the ER. Whereas GI-SIM limits the illumination volume to the basal region of a cell, 3D-SIM illuminates the entire cell volume, including portions outside the imaging plane. Thus, our experiments using 3D-SIM required the high photostability of the fluorescent ER marker. Using er-(n2)oxStayGold(c4), we imaged the ER network dynamics of multiple cells simultaneously and continuously for up to 6 minutes, without deterioration of super-resolution image quality (Fig. 4 and Supplementary Discussion 5).

A potential limitation of StayGold is its dimeric nature, and a monomeric version of StayGold is under development. To avoid potential complications that can arise when tagging membrane proteins with dimeric FPs, in our experiments we labeled the ER (Figs. 3 and 4) and the mitochondria (Extended Data Fig. 3 and Supplementary Fig. 12) by expressing StayGold as luminal soluble markers. Although it is possible that the dimeric structure is indispensable for the protein’s outstanding photostability, other oligomeric FPs were not exceptionally photostable, and successful monomerization of previous FPs did not, in most cases, affect their photostability substantially (Fig. 2c, Supplementary Fig. 4d and Supplementary Table 1c). Currently, StayGold cannot substitute for popular bright monomeric green-emitting FPs in all applications. mNeonGreen, for example, will still be the first choice for molecular fusion applications in conventional time-lapse imaging experiments where illumination power density can be kept to less than 0.5 W cm^−^^2^.

Substantial efforts have always been required to engineer wild-type FPs for use in fluorescence imaging. Because many wild-type FPs are oligomers, monomerization is one of the most important aims in any engineering effort for an FP (ref. ^42^). It is also noted that most wild-type FPs are sensitive to N- or C-terminal fusions and that the evolution of FPs as useful tools has involved substantial modifications of their N- and C-termini to develop fusion-tolerant FPs, such as the mFruit series^13^. Although successful engineering of truly monomeric forms of StayGold (mStayGold) with appropriate appendages at the N- and C-termini may require determination of the crystal structure, we are engaged in their directed evolution via random and rational mutagenesis. mStayGold will then enable the visualization and quantification of tagged proteins at low copy number expressed via genome editing techniques; it will also enable the tracking of such proteins at the single-molecule level in cells over extended periods of time.

## Methods

### Animals

Colonies of hydrozoan *C. uchidae* were obtained from the sea near Asamushi Marine Biological Station (Aomori Prefecture, Japan) and maintained in artificial seawater SEA LIFE (Marine Tech).

### Photomicrography of *C. uchidae*

Fluorescence images of polyps were taken in the dark with a fluorescence stereomicroscope (Olympus SZX12). Fluorescence and differential interference contrast images of medusae were taken with a confocal microscope (Nikon C1) (Fig. 1a–c and Supplementary Fig. 1a).

### Tissue homogenate preparation

Medusae were collected in Hiroshima Bay (Hiroshima Prefecture, Japan) for the preparation of tissue homogenates. Samples for spectroscopy were prepared using a glass Teflon homogenizer in PBS^−^ containing 10 μM E-64, 10 μM leupeptin and 1 μM Pep-A.

### RNA preparation

Total RNA was isolated from strain #17 female medusae using the NucleoSpin RNA Purification Kit (Macherey-Nagel) and concentrated by EtOH-LiCl precipitation.

### RNA-seq

Creation of RNA-seq cDNA libraries, sequencing and de novo transcriptome assembly were carried out by BGI to obtain 189,734 contigs and subsequently 102,642 unigene clusters. Then, BLAST searches were performed to identify unigene #1784 as a *C. uchidae* ortholog corresponding to hydrozoan GFPs (Supplementary Fig. 1b).

### Gene construction for bacterial expression (FP)

The CU17S gene was amplified using primers containing 5′-*Bam*HI and 3′-*Xho*I sites, and the restricted product was cloned in-frame into the *Bam*HI/*Xho*I sites of pRSET_B_ (Thermo Fisher Scientific) to generate pRSET_B_/CU17S, which was used as the template for mutagenesis. Random mutations were introduced using error-prone PCR. Bacterial cells transformed with mutagenized plasmids were screened for efficient chromophore maturation at 37 °C. The obtained product with mutation V168A was pRSET_B_/StayGold.

On the other hand, EGFP, SiriusGFP, mClover3 and mNeonGreen genes were amplified using primers containing 5′-*Bam*HI and 3′-*Eco*RI sites, and the restricted products were cloned in-frame into the *Bam*HI/*Eco*RI sites of pRSET_B_ to generate pRSET_B_/EGFP, pRSET_B_/SiriusGFP, pRSET_B_/mClover3 and pRSET_B_/mNeonGreen, respectively.

Likewise, mTFP1 (ref. ^23^), Venus^45^, Achilles^41^, mGold^10^, mOrange2 (ref. ^6^), mCherry^13^, mScarlet-I (ref. ^7^), mScarlet-H (ref. ^7^), mCardinal^46^, TagRFP-T (ref. ^6^), AmCyan^47^, tKeima^48^, dKeima^48^, KikG (ref. ^49^), h2-3 (ref. ^50^) and TurboRFP (ref. ^51^) genes were transferred to pRSET_B_ vector using the *Bam*HI and *Eco*RI sites.

### Gene construction for mammalian expression (cytosolic expression)

The gene for StayGold was re-synthesized with mammalian-preferred codons (h-StayGold). The 5′ end of h-StayGold, EGFP, SiriusGFP, mClover3, mNeonGreen or CU17S gene was modified by PCR to have an *Xho*I site followed by the protein translation initiation site. *Xho*I/*Xba*I fragments encoding StayGold, EGFP, SiriusGFP, mClover3, mNeonGreen and CU17S were subcloned into pCSII-EF to generate pCSII-EF/StayGold, pCSII-EF/EGFP, pCSII-EF/SiriusGFP, pCSII-EF/mClover3, pCSII-EF/mNeonGreen and pCSII-EF/CU17S, respectively. The mCherry gene was amplified using primers containing 5′-*Xho*I and 3′-*Xba*I sites, and the restricted product was cloned into the *Xho*I/*Xba*I sites of pCSII-EF to generate pCSII-EF/mCherry.

### Gene construction for mammalian expression (bicistronic expression)

The T2A (ref. ^44^) gene was synthesized with 5′-*Hin*dIII and 3′-*Eco*RI sites, and the restricted product was cloned into the *Hin*dIII/*Eco*RI sites of pBlueScript (pBS) to generate pBS/T2A. The mCherry gene was amplified using primers containing 5′-*Xho*I and 3′-*Hin*dIII sites, and the restricted product was cloned in-frame into the *Xho*I/*Hin*dIII sites of pBS/T2A to generate pBS/mCherry-T2A. The green-emitting FP (h-StayGold, EGFP, SiriusGFP, mClover3 or mNeonGreen) gene was amplified using primers containing 5′-*Bam*HI and 3′-*Xba*I sites, and the restricted product was cloned in-frame into the *Bam*HI/*Xba*I sites of pBS/mCherry-T2A to generate pBS/mCherry-T2A-green-emitting FP. Lastly, *Xho*I/*Xba*I fragments encoding mCherry-T2A-green-emitting FP were subcloned into pCSII-EF to generate pCSII-EF/mCherry-T2A-green-emitting FP plasmids.

### Gene construction for mammalian expression (ER targeting)

First, (n2)StayGold was constructed by inserting amino acids 4–12 of EGFP between residues 3 and 4 of StayGold. Second, er-(n2)StayGold was generated by extending (n2)StayGold at the N-terminus with the signal peptide from calreticulin and at the C-terminus with an ER retention signal (KDEL). Third, all the five cysteine residues (Cys40, Cys150, Cys165, Cys174 and Cys208) of er-(n2)StayGold were subjected to site-directed random mutagenesis according to a published protocol^52^ where multiple degenerative primers were used to mutate amino acid residues randomly at multiple sites simultaneously. Screening for bright fluorescence labeling of the ER of transfected HeLa cells revealed that substituting Ile for both Cys174 and Cys208 was effective, whereas the other cysteines seemed to be indispensable. The resulting construct called er-(n2)oxStayGold was found to have the additional mutation His169Tyr accidentally. The er-(n2)oxStayGold gene was subcloned into the *Hin*dIII/*Eco*RI site of pcDNA3. Finally, er-(n2)oxStayGold(c4) was constructed by inserting amino acids 223–233 of dfGFP (ref. ^24^) into the C-terminal region of er-(n2)oxStayGold.

### Protein purification

Recombinant proteins with a polyhistidine tag at the N-terminus were expressed in *E. coli* (JM109 (DE3)). Transformed *E. coli* was incubated in a Luria–Bertani medium containing 0.1 mg ml^−1^ of ampicillin at room temperature with gentle shaking for several days. Protein purification by Ni^2+^ affinity chromatography was performed as described previously^53^.

### In vitro spectroscopy

Absorption spectra were acquired using a spectrophotometer (U-2910, Hitachi). Fluorescence excitation and emission spectra were acquired using a fluorescence spectrophotometer (F-2500, Hitachi). Absolute fluorescence quantum yields were measured using an absolute photoluminescence quantum yield spectrometer (C9920-02, Hamamatsu Photonics). Protein concentrations were measured using a Protein Assay Dye Reagent Concentrate Kit (5000006, Bio-Rad) with BSA as the standard.

### pH titrations

Measurement was performed at room temperature (25 °C) immediately after pH adjustment. Absorption was measured at the protein concentration of 10 μM using a U-2910 spectrophotometer (Hitachi). The following buffers were used to adjust pH:

pH 3: 50 mM Glycine-HCl buffer

pH 4–5: 100 mM CH_3_COONa-CH_3_COOH buffer

pH 6: 100 mM MES (NaOH) buffer

pH 7–8: 100 mM HEPES (NaOH) buffer

pH 9–10: 100 mM Glycine-NaOH buffer

pH 11: 100 mM Na_2_HPO_4_-NaOH buffer

pH 12: 100 mM KCl-NaPH buffer

See Supplementary Fig. 3.

### Analytical ultracentrifugation

Sedimentation equilibrium experiments were carried out as described previously^54^. Purified recombinant StayGold at 2.8 μM in 50 mM HEPES-NaOH (pH 7.4) was analyzed on a Beckman Optima XL-A analytical ultracentrifuge at 24 °C, and absorbance was measured at 497 nm (Supplementary Fig. 2a).

### Pseudonative SDS-PAGE analysis

Non-heated protein samples were separated on 10% polyacrylamide gels as described previously^55^. SDS-PAGE molecular weight standards, HR (Bio-Rad, 161-0303) and LR (Bio-Rad, 161-0304) were used (Supplementary Fig. 2b).

### Gene construction for mammalian expression (mitochondrial targeting)

(n1)StayGold was constructed by inserting amino acids 5–13 of EGFP between residues 4 and 5 of StayGold. mt-StayGold and mt-(n1)StayGold were constructed by fusing a tandem repeat of the cytochrome c oxidase subunit VIII pre-sequence^27^ to the N-termini of StayGold and (n1)StayGold, respectively.

### Gene construction for mammalian expression (subcellular targeting)

The cDNA fragments encoding (n1)StayGold(c4)-21aa-β-tubulin, (n1)StayGold(c4)-21aa-β-actin, (n1)StayGold(c4)-28aa-α-tubulin and StayGold(c4)-20aa-Giantin were generated based on pBS Coupler 4 and 6 (ref. ^56^).

The (n1)StayGold(c4) gene was amplified using primers containing 5′-*Not*I and 3′-*Eco*RI sites. Also, the β-tubulin gene was amplified using primers containing 5′-*Hin*dIII and 3′-*Xho*I sites. The two restricted products were sequentially cloned into the *Not*I/*Eco*RI and *Hin*dIII/*Xho*I sites of pBS Coupler 4. Finally, the *Not*I/*Xho*I fragment was cloned into pcDNA3 to generate pcDNA3/(n1)StayGold(c4)-21aa-β-tubulin.

The (n1)StayGold(c4) gene was amplified using primers containing 5′-*Not*I and 3′-*Eco*RI sites. Also, the β-actin gene was amplified using primers containing 5′-*Hin*dIII and 3′-*Xho*I sites. The two restricted products were sequentially cloned into the *Not*I/*Eco*RI and *Hin*dIII/*Xho*I sites of pBS Coupler 4. Finally, the *Not*I/*Xho*I fragment was cloned into pcDNA3 to generate pcDNA3/(n1)StayGold(c4)-21aa-β-actin.

The (n1)StayGold(c4) gene was amplified using primers containing 5′-*Not*I and 3′-*Bam*HI sites. Also, the α-tubulin gene was amplified using primers containing 5′-*Hin*dIII and 3′-*Xho*I sites. The two restricted products were sequentially cloned into the *Not*I/*Bam*HI and *Hin*dIII/*Xho*I sites of pBS Coupler 6. Finally, the *Not*I/*Xho*I fragment was cloned into pcDNA3 to generate pcDNA3/(n1)StayGold(c4)-28aa-α-tubulin.

The StayGold(c4) gene was amplified using primers containing 5′-*Bam*HI and 3′-*Eco*RI sites. Also, the gene encoding amino acids 3,131–3,259 of human Giantin^7^ was amplified using primers containing 5′-*Hin*dIII and 3′-*Xho*I sites. The two restricted products were sequentially cloned into the *Bam*HI/*Eco*RI and *Hin*dIII/*Xho*I sites of pBS Coupler 6. Finally, the *Not*I/*Xho*I fragment was cloned into pcDNA3 to generate pcDNA3/StayGold(c4)-20aa-Giantin.

pcDNA3/tau-6aa-StayGold, N1/Lifeact-2aa-StayGold, pcDNA3/Lyn-2aa-StayGold and pCS2/StayGold-2aa-CAAX were produced by replacing the YFP gene in pcDNA3/tau-YFP (ref. ^48^) with an *Eco*RI/*Xho*I fragment of StayGold; replacing the mCherry gene in mCherry-Lifeact-7 (Addgene, 54491) with a *Bam*HI/*Not*I fragment of StayGold; replacing the AzamiGreen gene in Lyn-AG/pcDNA3 (ref. ^55^) with a *Bam*HI/*Eco*RI fragment of StayGold; and replacing the EGFP gene in EGFP-CAAX/pCS2 (ref. ^57^) with a *Bam*HI/*Eco*RI fragment of StayGold. Lyn: 22 N-terminal amino acids of the non-receptor tyrosine kinase; CAAX: 20 C-terminal amino acids of K-Ras (Supplementary Fig. 9).

### Gene construction (Nb–FP fusion)

The Nb(S1) gene was amplified using primers containing 5′-*Bam*HI and 3′-*Eco*RI sites, and the restricted product was cloned into the *Bam*HI/*Eco*RI sites of pBS Coupler 4 (ref. ^56^) to generate pBS/Nb(S1) = . ‘=’ denotes ‘Coupler linker’, a triple repeat of the amino acid linker Gly-Gly-Gly-Gly-Ser ((GGGGS)_3_). The h-StayGold gene was amplified using primers containing 5′-*Hin*dIII and 3′-*Sal*I sites, and the restricted product was cloned in-frame into the *Hin*dIII/*Sal*I sites of pBS/Nb(S1) = to generate pBS/Nb(S1) = StayGold. In parallel, pRSET_B_ was engineered to have an *Sal*I site instead of the *Hin*dIII site. The resulting plasmid was named pRSET_B_(*S*). The DNA fragment encoding Nb(S1) = StayGold was extracted from pBS/Nb(S1) = StayGold and cloned into the *Bam*HI/*Sal*I sites of pRSET_B_(*S*) to generate pRSET_B_(*S*)/Nb(S1) = StayGold. The DNA linker that encodes ‘EV linker’, a 29 repeat of the amino acid linker Ser-Ala-Gly-Gly (ref. ^29^), was synthesized. The DNA fragment was amplified using primers containing 5′-*Eco*RI and 3′-*Hin*dIII sites, and the restricted product was cloned into the *Eco*RI/*Hin*dIII sites of pRSET_B_(*S*)/Nb(S1) = StayGold to generate pRSET_B_(*S*)/Nb(S1) = = StayGold. ‘= =’ denotes the EV linker.

In addition, the Achilles^41^ gene was amplified using primers containing 5′-*Hin*dIII and 3′-*Sal*I sites, and the restricted product was cloned into the *Hin*dIII/*Sal*I sites of pRSET_B_(*S*)/Nb(S1) = StayGold to generate pRSET_B_(*S*)/Nb(S1) = Achilles.

### Gene construction (tdStayGold)

pRSET_B_(*S*)/Nb(S1) = = StayGold (see ‘Gene construction (Nb–FP fusion)’) was used as a starting material. First, the (n1)StayGold gene was amplified using primers containing 5′-*Hin*dIII and 3′-*Sal*I sites, and the restricted product was cloned in-frame into the *Hin*dIII/*Sal*I sites of pRSET_B_(*S*)/Nb(S1) = = StayGold to generate pRSET_B_(*S*)/Nb(S1) = = (n1)StayGold. Next, the (n1)StayGold(c4) gene was amplified using primers containing 5′-*Bam*HI and 3′-*Eco*RI sites, and the restricted product was cloned in-frame into the *Bam*HI/*Eco*RI sites of pRSET_B_(*S*)/Nb(S1) = = (n1)StayGold to generate pRSET_B_(*S*)/(n1)StayGold(c4) = = (n1)StayGold, namely pRSET_B_(*S*)/tdStayGold (Supplementary Fig. 13a).

### Gene construction for mammalian expression (microtubule plus-end targeting)

The EB3 gene was amplified using primers containing 5′-*Bam*HI and 3′-*Eco*RI sites, and the restricted product was cloned in-frame into the *Bam*HI/*Eco*RI sites of pBS Coupler 4 (ref. ^56^) to generate pBS/EB3 = . The tdStayGold gene was amplified and subcloned into the *Hin*dIII/*Xho*I sites of pBS/EB3 = using In-Fusion (Takara Bio) to generate pBS/EB3 = tdStayGold. Finally, a *Bam*HI/*Xho*I fragment encoding EB3 = tdStayGold was subcloned into pcDNA3 to generate pcDNA/EB3 = tdStayGold (Extended Data Fig. 4a).

### Gene construction (tdoxStayGold)

The (n1)oxStayGold(c4) gene was amplified using primers containing 5′-*Bam*HI and 3′-*Eco*RI sites. The EV linker^29^ gene was amplified using primers containing 5′-*Eco*RI and 3′-*Hin*dIII sites. The (n1)oxStayGold gene was amplified using primers containing 5′-*Hin*dIII and 3′-*Xho*I sites. The restricted products were cloned into the *Bam*HI/*Xho*I sites of pRSET_B_ to generate pRSET_B_/tdoxStayGold.

### Gene construction for mammalian expression (PSD targeting)

The PSD-95 gene^58^ was amplified using primers containing 5′-*Xho*I and 3′-*Eco*RI sites, and the restricted product was cloned into the *Xho*I/*Eco*RI sites of pEGFP-N1 (Clontech, Takara Bio) to generate pPSD-95-EGFP-N1. In parallel, the tdoxStayGold gene was amplified using primers containing 5′-*Bam*HI and 3′-*Not*I sites, and the restricted product was cloned into the *Bam*HI/*Not*I sites of pBlueScript Coupler 1 (ref. ^56^). From the resultant plasmid, the *Kpn*I/*Not*I fragment encoding Coupler-tdoxStayGold (=tdoxStayGold) was prepared and cloned into the *Kpn*I/*Not*I sites of pPSD-95-EGFP-N1. The substitution of the =tdoxStayGold gene for the EGFP gene resulted in the generation of pPSD-95=tdoxStayGold-N1 for transfection of cultured neurons (Extended Data Fig. 4b).

### Cell culture, transfection and fixation

HeLa (HeLa.S3) cells were obtained from the American Type Culture Collection (ATCC) (CCL-2.2). COS-7 cells were obtained from the ATCC (CRL-1651). Cells were cultured on standard 35-mm glass-bottom dishes (Iwaki) in DMEM (Sigma-Aldrich) containing 5% FBS (AusGeneX Pty. Ltd.) supplemented with 4 mM L-glutamine (25030081, Thermo Fisher Scientific) and 1% penicillin–streptomycin (Nacalai Tesque). The cells were transfected with plasmid DNAs (0.5 μg of each) using Lipofectamine 2000 reagent (11668027, Thermo Fisher Scientific). After washing with PBS occasionally, the cells were fixed with 4% paraformaldehyde (PFA) at room temperature for 10 minutes.

### Lentivirus production

Replication-defective, self-inactivating lentiviral vectors were used^59^. The pCSII-EF-MCS vector encoding StayGold, EGFP, SiriusGFP, mClover3, mNeonGreen or mt-(n1)StyGold was co-transfected with the packaging plasmid (pCAG-HIVgp) and the VSV-G-/Rev-expressing plasmid (pCMV-VSV-G-RSV-Rev) into 293T cells. High-titer viral solutions were prepared and used for transduction into HeLa cells (MOI = 1–10). Most (>95%) of the resultant cells uniformly exhibited green fluorescence and were used as stable transformants: HeLa/StayGold, HeLa/EGFP, HeLa/SiriusGFP, HeLa/mClover3 and HeLa/mNeonGreen (Supplementary Fig. 5a). Uniform expression among cells was also confirmed in the HeLa/mt-(n1)StayGold line (Extended Data Fig. 3).

### Cell proliferation assay

HeLa/StayGold, HeLa/EGFP, HeLa/mNeonGreen and HeLa/mt-(n1)StayGold cells were tested in comparison with control HeLa cells. In total, 25,000 cells were seeded onto a standard 60-mm dish and maintained in growth medium (DMEM high glucose, supplemented with 10% FBS). Afterwards, cells were detached by trypsin and suspended in 1 ml of growth medium. Then, 25 μl of the cell suspension was mixed with 25 μl of 0.4% trypan blue solution (Sigma-Aldrich, F8154) for manual counting (Supplementary Fig. 5b).

### Cellular brightness assay

HeLa cells were seeded into 96-well glass-bottom plates (Matsunami Glass, GP96000) and maintained in growth medium (DMEM low glucose, supplemented with 10% FBS). On the following day, cells were transfected with cDNAs. Thirty hours after transfection, cells were imaged on an inverted microscope (IX-83, Olympus) equipped with an LED light bulb (X-Cite XYLIS, Excelitas Technologies), an objective lens (Olympus, UPlanXApo ×4/0.16 NA) and a scientific CMOS camera (ORCA-Fusion, Hamamatsu Photonics). Green-emitting FPs were observed using a filter cube (U-FBNA, Olympus). mCherry was observed using a filter cube (U-FMCHE, Olympus). Cells were transfected with 0.2 μg of pCSII-EF/mCherry-T2A-green-emitting FP per well. Green-emitting FP fluorescence was divided by mCherry fluorescence and normalized to the ratio of EGFP/mCherry as described previously^43^ (Table 1 and Supplementary Fig. 7b).

### WF photobleaching (purified protein)

An FP solution was mixed with 30% (w/v) acrylamide (Wako, 012-08023). Immediately after the addition of 10% ammonium persulfate (Wako, 202-04003) and N,N,N′,N′-tetramethylethylenediamine (Wako, 202-04003), 200 μl of the mixture was poured onto a 35-mm glass-bottom dish (Iwaki, 3911-035) and overlaid with a coverslip (No. 1, 0.13–0.17-mm thickness, Matsunami Glass). Sandwiched between the two coverslips was 1 μM FP gel embedded in 20% polyacrylamide. The protein sample was excited continuously on an inverted microscope (IX-81, Olympus) equipped with a standard 75-W xenon lamp, a ×40 objective lens (UPlanSApo ×40/0.95 NA) and a cooled CCD camera (ORCA AG, Hamamatsu Photonics). An appropriate excitation filter was used to choose the excitation wavelength. Whereas no neutral density (ND) filter was installed in the illuminator in principle, appropriate ND filters (1–12% transmittance) were used to attenuate the emitted fluorescence. Image acquisition was performed using an appropriate emission filter every 6 seconds with a short exposure time (250–360 ms). The whole system was controlled using AQUACOSMOS software (Hamamatsu Photonics).

The following experimental conditions were used for eight FP groups:

#### Green-emitting FP (StayGold, EGFP, SiriusGFP, mClover3 and mNeonGreen)

See Figs. 1h,i and 2b, Table 1 and Supplementary Table 1b. The following excitation filters, dichroic mirrors and emission filters combined with ND filters were used:

Exciter: 488.0 IF 10 (488 ± 5 nm) (Cheshire Optical)

Dichroic mirror: DM505 (Olympus)

The excitation light density above the objective was 5.6 W cm^−^^2^.

Emitter: BA510IF (510 nm < ) (Olympus) combined with NDX006 (6% transmittance) (Asahi Spectra)

#### Green-emitting FP (KikG, h2-3)

See Fig. 2c and Supplementary Table 1c. The following excitation filters, dichroic mirrors and emission filters combined with ND filters were used:

Exciter: 488.0 IF 10 (488 ± 5 nm) (Cheshire Optical)

Dichroic mirror: DM505 (Olympus)

The excitation light density above the objective was 5.4 W cm^−^^2^.

Emitter: BA510IF (510 nm < ) (Olympus) combined with NDX006 (6% transmittance) (Asahi Spectra)

#### Green-emitting FP (CU17S, oxStayGold and tdStayGold)

See Fig. 2a and Supplementary Table 1a. The following excitation filters, dichroic mirrors and emission filters combined with ND filters were used:

Exciter: 488.0 IF 10 (488 ± 5 nm) (Cheshire Optical)

Dichroic mirror: DM505 (Olympus)

The excitation light density above the objective was 5.6 W cm^−^^2^.

Emitter: BA510IF (510 nm < ) (Olympus) combined with NDX006 (6% transmittance) (Asahi Spectra)

#### Cyan-emitting FP (mTFP1 and AmCyan)

See Fig. 2b,c and Supplementary Table 1b,c. The following excitation filters, dichroic mirrors and emission filters combined with ND filters were used:

Exciter: 435.8 IF 10 (435.8 ± 5 nm) (Cheshire Optical)

Dichroic mirror: XF2034, 455DRLP (Omega Optical)

The excitation light density above the objective was 4.1 W cm^−^^2^.

Emitter: XF3075, 480AF30 (480 ± 15 nm) (Omega Optical)

#### Red-emitting FP with blue light excitation (dKeima and tKeima)

See Fig. 2c and Supplementary Table 1c. The following excitation filters, dichroic mirrors and emission filters combined with ND filters were used:

Exciter: 435.8 IF 10 (435.8 ± 5 nm) (Cheshire Optical)

Dichroic mirror: XF2034, 455DRLP (Omega Optical)

The excitation light density above the objective was 4.1 W cm^−^^2^.

Emitter: BA575IF (575 nm < ) (Olympus) combined with NDX050 (50% transmittance) (Asahi Spectra)

#### Yellow-emitting FP (Venus, Achilles, mVenus and mGold)

See Fig. 2b and Supplementary Table 1b. The following excitation filters, dichroic mirrors and emission filters combined with ND filters were used:

Exciter: 514.5 IF 10 (514.5 ± 5 nm) (Cheshire Optical)

Dichroic mirror: XF2030, 525DRLP (Omega Optical)

The excitation light density above the objective was 3.4 or 1.16 W cm^−^^2^.

Emitter: XF3074, 545AF35 (545 ± 17.5 nm) (Omega Optical) combined with NDX012 (12% transmittance) (Asahi Spectra)

#### Orange-emitting FP (mOrange2), red-emitting FP (mScarlet-H, TagRFP-T, mScarlet-I, mCherry and TurboRFP)

See Fig. 2b,c and Supplementary Table 1b,c. The following excitation filters, dichroic mirrors and emission filters combined with ND filters were used:

Exciter: 550.0 IF 10 (550 ± 5 nm) (Cheshire Optical)

Dichroic mirror: DM570 (Olympus)

The excitation light density above the objective was 3.4 W cm^−^^2^.

Emitter: BA575IF (575 nm < ) (Olympus) combined with NDX006 (6% transmittance) (Asahi Spectra)

#### Far-red FP (mCardinal)

See Fig. 2b and Supplementary Table 1b. The following excitation filters, dichroic mirrors and emission filters combined with ND filters were used:

Exciter: XF1207, 580AF20 (580 ± 10 nm) (Omega Optical) combined with NDX050 (50% transmittance) (Asahi Spectra)

Dichroic mirror: XF2020, 600DRLP (Omega Optical)

The excitation light density above the objective was 4.6 W cm^−^^2^.

Emitter: XF3081, 645AF75 (645 ± 37.5 nm) (Omega Optical)

The data were analyzed using Excel (2019). The fluorescence intensity at *t* = 0 was normalized to 1,000 photons/s/molecule, and the time axis was adjusted according to the standard method^6^.

For the examination of reversible photobleaching, the shutter of the illuminator was closed manually for 1–3 minutes during the photobleaching experiment (Supplementary Fig. 18).

### WF photobleaching (living cells in HBSS)

Living cells on 35-mm glass-bottom dishes were incubated in Hanks’ Balanced Salt Solution (HBSS) containing 15 mM HEPES-NaOH (pH 7.4) and imaged on an inverted microscope (IX-81, Olympus) equipped with a standard 75-W xenon lamp, a ×40 objective lens (UPlanSApo ×40/0.95 NA) and a cooled CCD camera (ORCA-AG, Hamamatsu Photonics). The data were analyzed using Excel (2019). The fluorescence intensity at *t* = 0 was normalized to 1,000 photons/s/molecule, and the time axis was adjusted according to the standard method^6^ (Fig. 1i and Table 1).

### WF photobleaching (living cells in DMEM)

Living cells on 35-mm glass-bottom dishes were incubated in DMEM (high glucose) containing 10% FBS without L-glutamine and phenol red (Wako, 040-30095) and imaged on an inverted microscope (IX-81, Olympus) equipped with a standard 75-W xenon lamp, a ×40 objective lens (UPlanSApo ×40/0.95 NA) and a cooled CCD camera (ORCA-AG, Hamamatsu Photonics). The data were analyzed using Excel (2019). The fluorescence intensity at *t* = 0 was normalized to 1,000 photons/s/molecule, and the time axis was adjusted according to the standard method^6^ (Table 1 and Supplementary Fig. 6a).

For the generation of anoxic and hyperoxic conditions, a gas cylinder containing 95% N_2_ and 5% CO_2_ and a gas cylinder containing 95% O_2_ and 5% CO_2_, respectively, were purchased from TOMOE SHOKAI Co., Ltd. (Supplementary Fig. 6b).

### SIM for live imaging

Super-resolution 3D-SIM images were acquired continuously (without intervals) on an N-SIM S microscope (Nikon Instruments) equipped with an SR HP Plan Apo λS 100×C/1.35 NA silicone immersion objective and a CMOS camera (ORCA FUSION, Hamamatsu Photonics) at room temperature (25–30 °C). When mitochondrial fission/fusion events were observed, the temperature was set at 37 °C. Each frame was constructed from 15 raw images (five phases and three angles). In all cases, ‘slice reconstruction’ was used to obtain confocal-like optically sectioned images. Image acquisition and analysis were carried out with NIS-Elements AR (version 5.30.00) (Supplementary Videos 3–5).

### Assessment of SIM image quality

Scores of reconstruction results of 3D-SIM are as follows (from the manual of N-SIM).

8: Reconstruction is executed normally. Every separation component is judged as an effective signal and is reflected on the reconstructed image.

7: The 2nd order diffraction component in one direction is judged as a defective signal; the reconstructed image is generated without the 2nd order diffraction component.

6: The 2nd order diffraction components in two directions are judged as defective signals; the reconstructed image is generated without these components.

5: The 1st order and the 2nd order diffraction components in one direction are judged as defective signals; the reconstructed image is generated without these components.

4: The 2nd order diffraction components in three directions are judged as defective signals; the reconstructed image is generated without these components.

3: The 1st order diffraction component in one direction and 2nd order diffraction components in the other directions are judged as defective signals; the reconstructed image is generated without these components.

2: The 1st order diffraction component in one direction and 2nd order diffraction components in the other two directions are judged as defective signals; the reconstructed image is generated without these components.

1: The 1st order and the 2nd order diffraction components in two directions or more are judged as defective signals; the reconstructed image is generated without these components.

### Lattice SIM for live imaging

Super-resolution 3D-SIM images were acquired continuously on ZEISS Elyra 7 equipped with a PlanApo ×40/1.4 NA oil immersion objective at 37 °C. The Burst mode for lattice SIM was used to increase the temporal resolution. Image analysis was carried out with ZEN 2014 (version 9.1) (Extended Data Fig. 1 and Supplementary Video 2).

### Spinning disk confocal microscopy

Living cells on 35-mm glass-bottom dishes in HBSS containing 15 mM HEPES-NaOH (pH 7.4) were imaged at 30 °C on an inverted microscope (IX-83, Olympus) equipped with a Nipkow spinning disk unit (CSU X1, Yokogawa) and a ×100 silicone objective lens (UPlanSApo ×100/1.35 NA). Images were collected every 53 ms in the streaming mode using an iXon Ultra 897 EMCCD camera (Andor Technology) with gain ×300. The exposure time was set to 50 ms. Image acquisition and analysis were carried out with Olympus cellSens software (version 2.6) (Fig. 3b).

Living cultured neurons on 35-mm poly-ʟ-lysine-coated glass-bottom dishes were imaged using the SpinSR10 imaging system (Olympus) equipped with an ORCA-Flash 4.0 camera (Hamamatsu Photonics) and a ×100 oil objective lens (UPLAPO OHR ×100/1.50 NA). Image acquisition and analysis were carried out with Olympus cellSens software (version 3.1.1) (Extended Data Fig. 4b).

Purified FP proteins (1 μM FP solutions in polyacrylamide gel) between two coverslips were excited continuously on this microscopy system with a ×40 objective lens (UPlanSApo ×40/0.95 NA). Illumination intensity was 1.7 W cm^−^^2^. Image acquisition was performed every 30 seconds with a short exposure time (250 ms) (Supplementary Fig. 17).

### Automatic quantification of ER network rearrangement

Image processing was performed using a customized program based on C++ and OpenCV 3.4.1 (https://opencv.org). First, regions containing observed cells were selected for image processing, and binarized images were generated from reconstructed 3D-SIM images according to the conventional method^60^. Based on the Otsu method, threshold value was automatically optimized in each image to get the best binarization output; this was crucial for analyzing er-oxGFP images that showed weak fluorescence due to photobleaching (Supplementary Fig. 11). Second, the regions were divided into 16 × 16-pixel sub-blocks. As the widths of most ER tubules spanned several pixels, each sub-block was considered sufficiently small to efficiently detect alterations of ER network morphology. Third, for each sub-block, frame *n* – 1 and frame *n* were compared, and the proportion of pixels that underwent conversions between + and – was calculated. The calculation was repeated *n*  –  1 times throughout the regions. Finally, the calculated values were normalized into integers ranging from 0 to 255 for the generation of *n*  –  1 heat maps. The heat maps shown in Fig. 4 are pseudo-colored images that were generated using the ‘16 Colors’ of ImageJ (Extended Data Fig. 2b).

### Analysis of rapid motion of ER tubules

We selected regions of interest (ROIs) that contained ER tubules with relatively stable endpoints. ROIs were subjected to image processing, as follows. First, binarized images were created to mask fluorescent objects, which were mostly tubular structures. Second, we developed a program to crop 16-bit raw images according to masked regions. The program was also used to remove noise components that measured <20 pixel area. This design enabled us to characterize ER tubule structures in great detail (16-bit processing) for kymography. Third, we used ImageJ to manually draw the axis containing two endpoints. Fourth, using another customized program, we traced fluorescence signal distribution along the perpendicular bisector to generate kymographic data. After confirming the single peak characteristics, we plotted the position that gave the maximum intensity over time. Finally, we performed FFT analysis on a kymograph in a window consisting of 128 (2^7^) consecutive time points. As the temporal resolution of the imaging experiment (Extended Data Fig. 1 and Supplementary Video 2) was 134.47 Hz, the FFT spectra are given with a frequency unit of 1.05 (134.47/128) Hz. All the customized programs were made based on C++ and OpenCV 3.4.1 (https://opencv.org).

### Measurement of irradiance (W cm^−^^2^)

The power of excitation light (W) above the objective at the focal plane was measured using Laser Power Meter LP1 (Sanwa Electric Instrument). Our present study used mostly dry objectives, and this measurement was simply carried out. For immersion objectives, a device (IX3-EXMAD, Olympus) was used. The only exception was the SR HP Plan Apo λS 100×C /1.35 NA silicone immersion objective in the N-SIM S microscope. Assuming that the front lens with special coating prevents total internal reflection, we collected exiting light with airspace for power measurement. There is a special slide-based power meter (https://www.thorlabs.com/newgrouppage9.cfm?objectgroup_id=2191) that enables precise measurement of irradiance even in high-spatial-resolution imaging experiments.

The microscopy field diaphragm was stopped down to the utmost outer periphery of a visual field, the area (cm^2^) of which was calculated from the field number of an eyepiece and the magnification of an objective lens. Alternatively, the field diaphragm was maximally stopped down, and the diameter of the minimum field of view was measured by a micro-ruler for area (cm^2^) calculation.

In all cases of WF microscopy, the illuminator (collimator lens) was adjusted to achieve Köhler illumination. A color acrylic plate (Tokyu Hands) was placed at the focal plane to evaluate illumination uniformity on a CCD (CMOS) image.

### Monitoring FP maturation in living cells (early phase)

HeLa cells on 35-mm glass-bottom dishes were incubated in phenol red-free DMEM containing 10% FBS. After transfection with cDNA (1 μg) of StayGold or mNeonGreen, cells were subjected to time-lapse imaging using a fully automated confocal laser scanning microscope (Olympus, FV10i) equipped with a built-in CO_2_ incubator at 37 °C and an objective lens (Olympus, UPlanSApo ×10/0.40 NA). The h-StayGold gene was used in this assay. Both fluorescence and phase contrast (PC) images were acquired every hour after transfection. To observe green fluorescence from StayGold or mNeonGreen, a 473-nm diode laser and a 490–590-nm emission filter were used. After background subtraction, total fluorescence intensity was divided by the cell occupation area in each field. The areas were obtained by ImageJ (version 1.53h) after all cells were manually delineated on PC images. Four experiments were performed on different days (Supplementary Fig. 7a, left).

### Monitoring FP maturation in living cells (long term)

HeLa cells on 35-mm glass-bottom dishes were incubated in phenol red-free DMEM containing 10% FBS. After transfection with cDNA (1 μg) of StayGold or mNeonGreen, cells were subjected to long-term, time-lapse imaging using a fully automated confocal laser scanning microscope (Olympus, FV10i) equipped with a built-in CO_2_ incubator at 37 °C and an objective lens (Olympus, UPlanSApo ×10/0.40 NA). The h-StayGold gene was used in this assay. Both fluorescence and phase contrast images were acquired every 6 hours after transfection. To observe green fluorescence from StayGold or mNeonGreen, a 473-nm diode laser and a 490–590-nm emission filter were used. Binarized images were generated manually using ImageJ to delineate transfected cells. Total fluorescence intensity (FI) was divided by the transfected cell occupation area to give ‘FI/transfected cell area’ (Fig. 1m).

### FP maturation analysis (flow cytometry)

HeLa cells on a standard 100-mm dish (353003, Corning) were transfected with the cDNA of StayGold or mNeonGreen (6 μg of each) for 4 hours. The h-StayGold gene was used in this assay. Then, fresh growth medium DMEM (041-29775, Fuji Film) (10% FBS, penicillin–streptomycin) was substituted. Seventy-two hours after the transfection, cells were harvested and suspended in 1 ml of PBS. They were analyzed using a FACSAria II (BD Biosciences). Both StayGold and mNeonGreen were excited by a 488-nm laser line (laser diode), and their emission was collected through 530/30BP. The data were analyzed using FlowJo software (Tree Star) (Supplementary Fig. 7a, right).

### OSER

The cDNA fragment encoding CytERM was synthesized according to the sequence information of Emerald-CytERM-N-17 (Addgene, 56290) with 5′-*Hin*dIII and 3′-*Bam*HI sites. As the CytERM gene has a *Bam*HI, an *Eco*RI and a *Hin*dIII site internally, all these sites were eliminated on the synthesis. The (n1)StayGold or tdStayGold gene was amplified using primers containing 5′-*Bam*HI and 3′-*Xho*I sites. The restricted products were cloned into the *Hin*dIII/*Xho*I sites of pcDNA3 to generate pcDNA3/CytERM-(n1)StayGold or pcDNA3/CytERM-tdStayGold. One day after transfection, HeLa cells on a standard 35-mm glass-bottom dish were incubated in HBSS containing 15 mM HEPES-NaOH (pH 7.4) and imaged on an inverted microscope (IX-83, Olympus) equipped with a ×40 objective lens (UPlanXApo ×40/0.95 NA) and a camera (ORCA-FUSION, Hamamatsu Photonics). Multiple (5 × 5) *x–y* images were tiled using the stitch function of cellSens Dimension (Olympus) to cover a large field of view (1.7 mm × 1.7 mm). The number of transfected cells showing whorl structures was counted. Also, the number of transfected cells avoiding whorl formation was counted. In addition, typical cells were imaged using an inverted laser scanning confocal microscopy system (Olympus FV3000) equipped with a ×60 water objective lens (Olympus, UPlanApo ×60/1.2 NA). The size of the confocal aperture was 1 Airy disk. Confocal images were acquired every 1 μm along the *z*-axis to create z*-*stacks (ten slices) for maximum intensity projection. Three independent experiments were carried out for each construct: CytERM-(n1)StayGold or CytERM-tdStayGold (Supplementary Fig. 13).

### WF imaging (microtubule plus-end targeting)

Two days after transfection with pcDNA/EB3 = tdStayGold, COS-7 cells on a 35-mm glass-bottom dish incubated in HBSS containing 15 mM HEPES-NaOH (pH 7.4) were imaged on an inverted microscope (IX-70, Olympus) equipped with a standard 75-W xenon lamp, a ×60 water objective lens (Olympus, UPlanApo ×60/1.2 NA) and a cooled CCD camera (CoolSNAP HQ, Photometrics). An excitation filter (485DF12, Omega), an emission filter (FF01-536/40, Semrock) and a dichroic mirror (FF506-Di03, Semrock) were used. Image acquisition and analysis were carried out with MetaMorph (version 7.10.2.240) (Molecular Devices) (Extended Data Fig. 4a and Supplementary Video 6).

### Confocal imaging (subcellular targeting)

Two days after transfection, HeLa cells on 35-mm glass-bottom dishes were imaged using an inverted laser scanning confocal microscopy system (Olympus FV3000) equipped with a ×60 water objective lens (Olympus, UPlanApo ×60/1.2 NA). The size of the confocal aperture was 1 Airy disk.

In experiments of microtubule, filamentous actin and plasma membrane localizations (Supplementary Fig. 9a–c), cells were live imaged. When StayGold was localized to the Golgi apparatus (Supplementary Fig. 9d), cells were fixed with 4% PFA at room temperature for 5 minutes. Fixed cells were permeabilized in 0.1% Triton X-100/PBS for 30 minutes and then reacted with rabbit anti-GM130 polyclonal antibody (PM061, MBL, 1:500 dilution) for 1 hour and Alexa Fluor 647-conjugated donkey anti-rabbit IgG (A31573, Thermo Fisher Scientific, 1:500 dilution) for 1 hour. In addition, cell samples were stained with DAPI (D523, Fuji Film, 1:1,000 dilution). Confocal images were acquired every 1 μm along the *z*-axis to create z*-*stacks (20 slices).

### Cultured neurons

Primary neurons from rat brain hippocampus were prepared and maintained according to the standard method^61^. Transfection of cultured neurons was performed according to the standard method^62^.

### Kinetic binding measurement using Biacore (SPR)

Nb(S1) = = StayGold or Nb(S1) = Achilles was immobilized onto an NTA sensor chip. Binding was evaluated by injecting S1-Fc (Sino Biological, 40591-V02H) solutions serially diluted (5–160 nM) with the running buffer (10 mM HEPES (pH 7.4), 150 mM NaCl, 50 mM EDTA and 0.005% Tween 20). The runs were performed in the single-cycle kinetics mode with the following parameters:

Flow rate: 30 ml min^−1^

Association time: 180 seconds

Dissociation time: 400 seconds

After each cycle, the chip surface was regenerated by injecting 350 mM EDTA (pH 8.0) at 30 μl min^−1^ for 60 seconds. The resulting sensorgrams were fit to a 1:1 binding model using Biacore T200 Evaluation Software (Fig. 5d and Supplementary Fig. 16).

### Visualizing SARS-CoV-2 S protein in infected cells

Infection experiments were conducted within a biosafety cabinet class II type B2 inside a Biosafety Level 3 laboratory. SARS-CoV-2 KUH003 strain (DDBJ accession number LC630936) was isolated from a patient with COVID-19 who was hospitalized at Kitasato University Hospital^63^. VeroE6/TMPRSS2 cells were purchased from the Japanese Collection of Research Bioresources (JCRB) Cell Bank (JCRB1818). Cells were fixed with cold methanol for 20 minutes and then incubated in blocking solution (PBS containing 3% BSA and 1% Triton X-100) for 60 minutes at room temperature. After washing in PBS three times, the cells were reacted with 2–10 μg ml^−1^ of Nb(S1) = = StayGold or Nb(S1) = Achilles in blocking solution at room temperature for 60 minutes. After three PBS washes, the samples were fixed with 4% PFA at room temperature for 5–20 minutes. This staining reaction for viral spike protein was combined with immunostaining using the following antibodies in blocking solution:

#### ERGIC

Rabbit antibody to ERGIC3 (1:200, Abcam, ab129179) and donkey antibody to rabbit IgG conjugated to Alexa Fluor 555 (1:500, Thermo Fisher Scientific, A-31572)

#### Nsp8

Mouse mAb to Nsp8 (1:100, GeneTex, GTX632696) and donkey antibody to mouse IgG conjugated to Alexa Fluor 555 (1:500, Thermo Fisher Scientific, A-31570)

#### dsRNA

Mouse mAb to dsRNA (1:100, Merck, MABE1134-100UL) and donkey antibody to mouse IgG conjugated to Alexa Fluor 555 (1:500, Thermo Fisher Scientific, A-31570)

#### Plasma membrane

Mouse mAb to pan-cadherin (1:250–500, Sigma-Aldrich, C1821) and donkey antibody to mouse IgG conjugated to Alexa Fluor 647 (1:500, Thermo Fisher Scientific, A-31571).

In addition, nuclear staining was performed using either DAPI (1:1,000, Fuji Film, 340-07971) or Vybrant DyeCycle Ruby Stain (1:500–1,000, Thermo Fisher Scientific, V10309) at room temperature in PBS for 5–15 minutes, respectively.

WF images were acquired using an inverted microscope (IX-70, Olympus) equipped with a standard 75-W xenon lamp, a ×10 dry objective (UPlanApo, NA 0.40), a ×60 water immersion objective (UPlanApo/IR, NA 1.20) and a cooled CCD camera (CoolSNAP HQ2, Photometrics). A 485DF15 (Omega) excitation filter, an FF506-Di03 (Semrock) dichroic mirror and an FF01-536/40 (Semrock) emission filter were used to observe StayGold fluorescence. A U-MRFPHQ filter cube (excitation: 535-555HQ, dichroic mirror: 565, emission: 570-625HQ, Olympus) was used to observe Alexa 555 fluorescence. An XF407 filter cube (excitation: 635QM30, dichroic mirror: 660DRLP, emission: 710QM80, Omega) was used to observe Alexa 647 or Vybrant fluorescence. A filter cube (excitation: 365QM35, dichroic mirror: 420DCLP, emission: 480QM30) was used to observe DAPI fluorescence. Image acquisition and analysis were carried out with MetaMorph (version 7.10.2.240) (Molecular Devices) and Fiji/ImageJ (version 1.53h) (https://fiji.sc), respectively.

3D-SIM images were acquired on an N-SIM S microscope (Nikon Instruments) equipped with an SR HP Plan Apo λS 100×C/1.35 NA silicone immersion objective and a CMOS camera (ORCA FUSION, Hamamatsu Photonics). Aiming at comprehensive mapping, in principle, volumetric imaging was performed in *z-*steps of 0.12 μm. Imaged volumes comprised 39–79 *z-*slices. StayGold and Alexa 555 were excited at 488 nm and 561 nm alternately in each frame to visualize the co-localization between S and ERGIC, Nsp8 or dsRNA. On the other hand, DAPI and Alexa 647 were excited by a 405-nm laser line and a 647-nm laser line, respectively, in the epi-illumination mode. Deconvoluted images of nucleus and plasma membrane were generated using the Richardson–Lucy algorithm. Volume rendering was performed using Volocity (version 6.3.1, Quorum Technologies).

### Statistical analysis

For comparison between StayGold and mNeonGreen (Supplementary Fig. 7a), the normality of the data was first assessed using the Kolmogorov–Smirnov test. Then, the statistical difference was determined by Welch’s unpaired two-sided *t-*test. Differences with *P* < 0.01 were considered significant. Origin Pro (version 2020b) was used for the analysis.

### Reporting Summary

Further information on research design is available in the Nature Research Reporting Summary linked to this article.

## Online content

Any methods, additional references, Nature Research reporting summaries, source data, extended data, supplementary information, acknowledgements, peer review information; details of author contributions and competing interests; and statements of data and code availability are available at 10.1038/s41587-022-01278-2.

## Supplementary information

Supplementary Information Supplementary Discussions 1–5, Supplementary Figs. 1–18, Supplementary Table 1, captions for Supplementary Videos 1–6 and Supplementary References

Reporting Summary

Supplementary Video 1 **Direct photostability comparison between StayGold and mNeonGreen or EGFP in living HeLa cells in a single field of view of WF microscopy**. First half: StayGold versus mNeonGreen. See Fig. 1j. Second half: StayGold versus EGFP. See Fig. 1k. Elapsed times (hours:minutes:seconds).

Supplementary Video 2 **Visualizing rapid motion of ER tubules by using a new 3D-SIM technique that achieves nanoscale resolution on a millisecond time scale**. A COS-7 cell expressing er-(n2)oxStayGold(c4) was imaged by lattice SIM (Elyra 7) continuously for 5.473 seconds. The total number of acquired frames was 736. Thus, the temporal resolution was 134.47 frames per second. The cell-wide field of view covered the ER network in the peripheral and perinuclear regions. This movie (11.5 MB) was generated by considerable compression of the original large-volume video data (8.98 GB). Compression was made using TMPGEnc. By minimizing the exposure time of each image (1 ms), owing to the Burst mode in the lattice SIM system, it was possible for us to visualize ER dynamics at a temporal resolution of >300 frames per second with similar image dataset quality. Whereas SIM-based techniques enabling high-speed imaging of the ER, such as iSIM (instant SIM) (100 Hz) (ref. ^11^) and GI-SIM (266 Hz) (ref. ^7^), were previously reported, their performance was assessed by use of ER markers that contained conventional *Aequorea* GFP variants. It is expected that er-(n2)oxStayGold(c4) will be effectively combined with these techniques (Extended Data Fig. 1).

Supplementary Video 3 **Agonist-induced and antagonist-induced longitudinal changes in ER structures revealed by fast, sustainable, cell-wide 3D-SIM imaging**. Three neighboring HeLa cells expressing er-(n2)oxStayGold(c4) were imaged by 3D-SIM (N-SIM S) continuously at a temporal resolution of 2.6 frames per second for 6 minutes. 10 μM histamine and 100 μM cyproheptadine were applied at 2 minutes and 4 minutes, respectively. This video (14.3 MB) was generated by considerable compression of the original large-volume movie data (14.3 GB). Compression was made using TMPGEnc. Elapsed times (minutes:seconds) (Fig. 4a–d).

Supplementary Video 4 **Agonist-induced and antagonist-induced longitudinal changes in ER structures revealed by fast, sustainable, cell-wide 3D-SIM imaging**. Three neighboring HeLa cells expressing er-(n2)oxStayGold were imaged by 3D-SIM (N-SIM S) continuously at a temporal resolution of 1.1 frames per second for 6 min. 10 μM histamine and 100 μM cyproheptadine were applied at 2 minutes and 4 minutes, respectively. This video (12.7 MB) was generated by considerable compression of the original large-volume movie data (6.10 GB). Compression was made using TMPGEnc. Elapsed times (minutes:seconds) (Fig. 4f–i).

Supplementary Video 5 **Sustainable and cell-wide 3D-SIM imaging for visualizing mitochondria**. A HeLa cell expressing mt-StayGold was imaged by 3D-SIM (N-SIM S) continuously at 0.72 frames second for 5 minutes. Raw (left) and reconstructed (right) images are presented side by side. Elapsed times (minutes:seconds) (Supplementary Fig. 12a).

Supplementary Video 6 **Visualizing microtubule plus-end dynamics by sustainable WF microscopy imaging**. COS-7 cells expressing EB3 = tdStayGold were imaged continuously at 2 frames per second for 30 minutes. Elapsed times (minutes:seconds) (Extended Data Fig. 4a).

## Data Availability

The accession numbers in the DDBJ/EMBL/GenBank databases are LC593677 for CU17S, LC601652 for StayGold, LC593679 for h-StayGold and LC601653 for oxStayGold. All data generated in this study are available through the RIKEN Research Data and copyrighted-work Management System (https://dmsgrdm.riken.jp/9gnxe/). Plasmid DNAs containing StayGold and its variants are available from the RIKEN Bio-Resource Center (http://en.brc.riken.jp) under a material transfer agreement with RIKEN. The K-874A gene is available from K.K. at Kitasato University upon reasonable request.

## Code availability

The custom-made software programs for ‘Quantification of ER network rearrangement’ and ‘Analysis of rapid motion of ER tubules’ are available through the RIKEN Research Data and copyrighted-work Management System (https://dmsgrdm.riken.jp/9gnxe/).
