# Author Correction: A highly photostable and bright green fluorescent protein

**DOI:** 10.1038/s41587-022-01469-x

**Published:** 2022-08-17

**Authors:** Masahiko Hirano, Ryoko Ando, Satoshi Shimozono, Mayu Sugiyama, Noriyo Takeda, Hiroshi Kurokawa, Ryusaku Deguchi, Kazuki Endo, Kei Haga, Reiko Takai-Todaka, Shunsuke Inaura, Yuta Matsumura, Hiroshi Hama, Yasushi Okada, Takahiro Fujiwara, Takuya Morimoto, Kazuhiko Katayama, Atsushi Miyawaki

**Affiliations:** 1 Biotechnological Optics Research Team, RIKEN Center for Advanced Photonics, Saitama, Japan; 2 Laboratory for Cell Function Dynamics, RIKEN Center for Brain Science, Saitama, Japan; 3 Asamushi Research Center for Marine Biology, Tohoku University, Aomori, Japan; 4 Department of Biology, Miyagi University of Education, Sendai, Japan; 5 Department of Infection Control and Immunology, Ōmura Satoshi Memorial Institute, Kitasato University, Tokyo, Japan; 6 Safety Science Laboratories, Kao Corporation, Tokyo, Japan; 7 Laboratory for Cell Polarity Regulation, RIKEN Center for Biosystems Dynamics Research, Osaka, Japan; 8 Department of Cell Biology and Department of Physics, UBI and WPI-IRCN, The University of Tokyo, Tokyo, Japan; 9 Institute for Integrated Cell-Material Sciences, Kyoto University, Kyoto, Japan; 10 Present Address: Graduate School of Integrated Sciences for Life, Hiroshima University, Hiroshima, Japan; 11 Present Address: Narita Elementary School, Miyagi, Japan

**Keywords:** Fluorescence imaging, Fluorescent proteins, Wide-field fluorescence microscopy, Super-resolution microscopy, Super-resolution microscopy

Correction to: *Nature Biotechnology* 10.1038/s41587-022-01278-2, published online 25 April 2022.

In the version of this article initially published, there were errors in the sequences shown in Supplementary Fig. 8, which has been replaced in the online version of the article.

