# Supplementary material for: A highly photostable and bright green fluorescent protein
Source: Nat Biotechnol. 2022 Apr 25;40(7):1132–42. doi: 10.1038/s41587-022-01278-2 (PMC9287174; doi:10.1038/s41587-022-01278-2)
Supplement: Supplementary file 1 — Supplementary Discussions 1–5, Supplementary Figs. 1–18, Supplementary Table 1, captions for Supplementary Videos 1–6 and Supplementary References [file 41587_2022_1278_MOESM1_ESM.pdf]

nature portfolio

https://doi.org/10.1038/s41587-022-01278-2

Supplementary information

A highly photostable and bright green fluorescent protein

In the format provided by the authors and unedited

Table of Contents

Supplementary Discussions 1–5

Supplementary Figures 1–18

Supplementary Table 1

Supplementary Video Captions 1–6

Supplementary References

Supplementary Discussions

Supplementary Discussion 1

The imaging modality that we have proposed through the 3D-SIM experiments using er-(n2)oxStayGold(c4) (Fig. 4a–d) required an extremely high photostability of the dyes for the following reasons.

(1) Due to the high motility of ER tubules, it was necessary to increase the imaging frame rate (2.6 frames/s). Accordingly, the illumination intensity was increased (2.4 W/cm^2) to realize sufficient photon budget for super-resolution SIM.

(2) Alterations of ER network morphology were quantified by successive image comparison between n-1 and n. Accordingly, continuous image acquisition (no intervals) was necessary throughout an experiment.

(3) To cover not only peripheral but also perinuclear ER, the entire cell was placed within the field of view.

(4) Unlike 2D-SIM (two-beam SIM), such as TIRF-SIM and GI-SIM, 3D-SIM (three-beam SIM) excited fluorophores both above and below the focal plane (ref. 1) (Supplementary Fig. 10).

(5) To examine the reversibility of the Ca^{2+} mobilization-dependent regulation of ER network dynamics, each imaging experiment spanned 6 min to enable the sequential administration of a histamine agent and an antihistamine agent.

Under these conditions, all the ER marker molecules existing inside individual cells were always exposed to intense illumination for a long time.

To verify whether er-oxGFP would be as robust to such a strong and long illumination as er-(n2)oxStayGold(c4), we first performed similar 3D-SIM experiments (2.4 W/cm^2, 3.1 frames/s, 6 min) but with no Ca^{2+} mobilization. We prepared four dishes that independently contained HeLa cells transfected with er-(n2)oxStayGold(c4) cDNA. In parallel, we prepared four dishes that contained HeLa cells transfected with er-oxGFP cDNA. The following day, we examined all the dishes by using a low NA objective and found that the statistical distribution of brightness was almost the same between er-(n2)oxStayGold(c4)- and er-oxGFP-expressing cells. We chose a few cells that were estimated to show the median intensity from each dish for the 3D-SIM imaging and plotted the fluorescence intensities of the observed cells against time (Supplementary Fig. 11a,e). We examined the quality of all the images for SIM reconstruction by using a function of the N-SIM S system, and obtained results that allowed us to distinguish er-(n2)oxStayGold(c4) from er-oxGFP (Supplementary Fig. 11b,f) clearly. Also, the FFT spectra of the images in the first and last frames were

analyzed in each experiment (Supplementary Fig. 11c,g) as are shown in Fig. 4c for the main experiment. These data indicated that the 3D-SIM images of er-oxGFP-expressing cells deteriorated a few minutes after the start of observation. In fact, the ER movement measurement with er-oxGFP was unstable and noiseful (Supplementary Fig. 11d,h). Next, we performed a 3D-SIM experiment with transient (2 min) Ca^{2+} mobilization and confirmed that er-oxGFP was unable to provide sustainable spatiotemporally improved images of the ER (Supplementary Fig. 11i). Notably, er-oxGFP-labeled tubules appeared to be fragmented and thickened at the final stage, which contrasted with er-(n2)oxStayGold(c4)-labeled tubules (Extended Data Fig. 2a).

In the ER imaging by 3D-SIM at 1–3 frames/s (Fig. 4a–d and Supplementary Fig. 11a–d), er-(n2)oxStayGold(c4) appeared to still have an advantage in terms of photostability. In an attempt, thus, we made an er-(n2)oxStayGold(c4)-expressing cell subjected to a much faster (134.47 frames/s) cell-wide 3D-SIM imaging that spanned only 5.473 s (Extended Data Fig. 1). However, further prolongation of the imaging time was not practical as the experimental conditions were limited by the data volume. The size of most of the 3D-SIM data from ER imaging experiments in this study was larger than 20 GB (See Data and materials availability).

Supplementary Discussion 2

Supplementary Fig. 12b shows a head-to-head comparison of mt-(n1)StayGold and mt-mNeonGreen. We chose transfected cells having similar initial brightness and came to the conclusion that mt-mNeonGreen is less capable of providing sustainable spatiotemporally improved images of mitochondria for cell-wide and long-term visualization of their fission and fusion than mt-(n1)StayGold.

Supplementary Discussion 3

Due to the poor accessibility of antibodies in the dense protein-rich assemblies of PSD, it is still difficult to fully immunolocalize endogenous PSD-95 molecules in neuronal samples (ref. 2). For this reason, FP-tagged PSD-95 was expressed to study the organization, recruitment, and stability of this protein. In many cases, FP-tagged PSD-95 was imaged comparatively before and a few tens of minutes after stimulation or was photobleached locally for subsequent measurement of fluorescence recovery, i.e., the mobility of this protein. However, as the overexpression of FP-tagged PSD-95 notoriously alters synaptic morphology and function (ref. 3), moderate expression levels are desired. Recent genetic methods that enable FP labeling of endogenous PSD-95 have shown much promise (refs. 4, 5). As speculated in the Discussion, it will be

possible to express PSD-95-tdoxStayGold molecules at a low copy number via genome-editing techniques and to track them at high spatiotemporal resolution for tens of minutes.

Supplementary Discussion 4

Among the physical properties of FPs, photobleaching is the most difficult to assess for the following three reasons.

First, the illumination mode drastically influences photobleaching efficiency. Single-photon excitation epifluorescence microscopes are categorized into two groups based on the illumination mode. On the one hand, wide-field microscopy employs Köhler illumination, and dyes in a specimen are constantly excited during the acquisition of each image. Classic wide-field microscopy is equipped with an arc lamp. In this study, we principally used this type of microscopy to investigate the photostability of FPs; our data demonstrate that StayGold is extremely stable. We have also shown that the wide-field microscopy technique SIM can bring out the best in StayGold in the context of sustainable super-resolution imaging. On the other hand, laser-scanning confocal microscopy (LSCM) employs critical illumination, and every dye in the focal plane is excited strongly but intermittently. It is notable that StayGold was somewhat susceptible to light from single-beam LSCM, suggesting that this FP is sensitive to very strong instantaneous illumination. Unfortunately, StayGold's photostability in this conventional LSCM was dependent on several factors including scanning speed, and consequently was difficult to quantify. By contrast, the multi-beam scanning method of spinning disk confocal microscopy did not degrade the photostability of StayGold (Fig. 3b, Supplementary Fig. 17).

Second, many FPs exhibit reversible photoswitching behavior, which is clearly observable by intermittent illumination (ref. 6). This behavior mostly arises from the *cis*-to-*trans* isomerization of the chromophore and should be distinguished from irreversible photobleaching. However, because there was no recovery in StayGold's fluorescence after constant illumination (Supplementary Fig. 18), we concluded that this FP's photobleaching has no reversible component.

Third, it is not easy to measure irradiance (W/cm^2) at the focal plane of objective lenses. On the one hand, the area of the illumination field (cm^2) is obtained by adjusting the microscope field stop. On the other hand, measurement of illumination power (W) is rather complicated. It is possible to collect all the exiting light for quantification by holding a conventional power meter over dry objectives. Our quantitative photobleaching experiments in this study used a dry objective (UPlanSApo

40×, Olympus), with which we were able to calculate irradiance values routinely. However, conventional power meters must be kept dry and cannot be used for immersion objectives, which usually have high numerical apertures (NAs). In the absence of an immersion liquid, such as water, silicone, or oil, it is not certain how much illumination light can exit from the front lens to reach a sensor tip. There is a special power meter on the market that enables precise measurement of irradiance even in high spatial resolution imaging experiments (see Methods). Because strong appeals have long been made regarding the necessity of sharing metadata about image data (refs. 7, 8) , and irradiance is one of the most important metadata, we hope that commercial microscopy systems will evolve to become amenable to the addition of irradiance measurement functions.

Supplementary Discussion 5

Guo et al. imaged tubular ER growth intensively together with microtubules (MTs) at 0.5 frames/s by dual-color GI-SIM (ref. 9). They proposed five different mechanisms for the morphological changes. Three mechanisms involve direct interaction with MTs, one mechanism involves hitchhiking on motile organelles, and the last mechanism appears as *de novo* ER budding. In this study, we did not focus on any specific ER events, but instead automatically quantified alterations of ER network morphology, including tubular ER growth, at 1.1–2.6 frames/s by 3D-SIM. We discovered that ER network dynamics are less mobile during Ca^{2+} mobilization. A variety of multi-color 3D-SIM imaging experiments will allow us to better understand this unexpected phenomenon. For example, it is challenging but important to image Ca^{2+} concentrations near or inside ER tubules at the same time as ER morphology. It is also interesting to observe MTs together to determine which of the aforementioned mechanism(s) is responsible for this Ca^{2+}-dependent behavior.

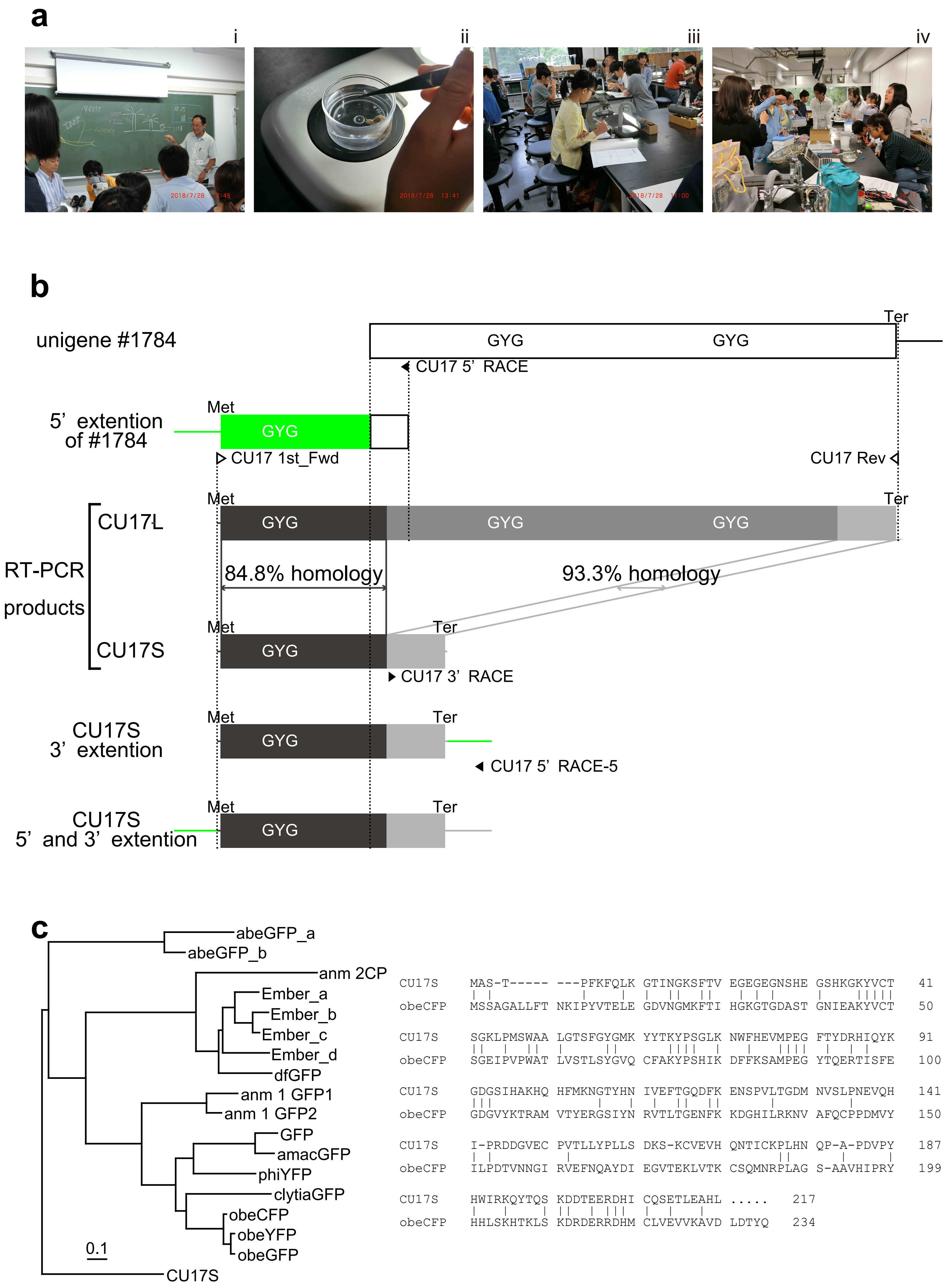

Supplementary Fig. 1 | CU17S derived from *C. uchidae*.

Supplementary Fig. 1 | CU17S derived from *C. uchidae.*

a, Contribution of *C. uchidae* to science education.

An experimental program using the fluorescent jellyfish *C. uchidae* to educate school children at Miyagi University of Education in Sendai. This program used to be held annually. The last program was held on July 28, 2018, and nineteen elementary students and three junior high school students participated (i).

Each student was given a set of jellyfish samples: a pair of medusae and a gastropod with polyps (ii), for observation by bright-field and fluorescence microscopy (iii).

The students were told that the origin of the green fluorescence remains unknown. The significance of the green fluorescence was discussed as a mystery (iv).

b, Molecular cloning of CU17S.

A unigene (#1784) was shown to encode a polypeptide consisting of multiple GFP-like domains. The polypeptide contains two GYG sequences, each of which could be responsible for chromophore synthesis. The polypeptide gene was extended by 5'-RACE-PCR using primer 'CU17 5' RACE' to its N-terminus that contained an additional GYG sequence. Next, RT-PCR using primers 'CU17 1st_Fwd' and 'CU17 Rev' provided two RNA transcripts encoding CU17L and CU17S. CU17L corresponded to protein product #1784, which appeared to be a three-domain FP. However, CU17L itself did not fluoresce in our expression systems. In addition, none of the domains fluoresced when expressed individually. On the other hand, CU17S appeared to be a single-domain FP. Interestingly, its N-terminal region (approximately three-quarters of the protein) had 84.8% similarity with the corresponding region of the first repeat of CU17L. Moreover, the remaining C-terminal region had 93.3% similarity with the corresponding region of the third repeat of CU17L. To validate the existence of the CU17S transcript, the RT-PCR product was extended in the 3' direction using primer 'CU17 3' RACE' and then in the 5' direction using primer 'CU17 5' RACE-5'. Both of these anchored PCR and RT-PCR analyses used total RNA prepared from *C. uchidae* strain #17. Primers used for RACE-PCR are indicated by solid arrowheads. Primers used for RT-PCR are indicated by open arrowheads. Met: methionine at N-terminus. Ter: termination. The region newly extended by RACE-PCR is depicted in green.

c, Unique primary structure of CU17S.

left, Phylogenetic tree of known hydrozoan GFP-like proteins. It is evident that CU17S is evolutionarily distant from other known FPs.

right, Amino acid sequence alignment of CU17S and obeCFP, an FP cloned from *Obelia* medusa.

a

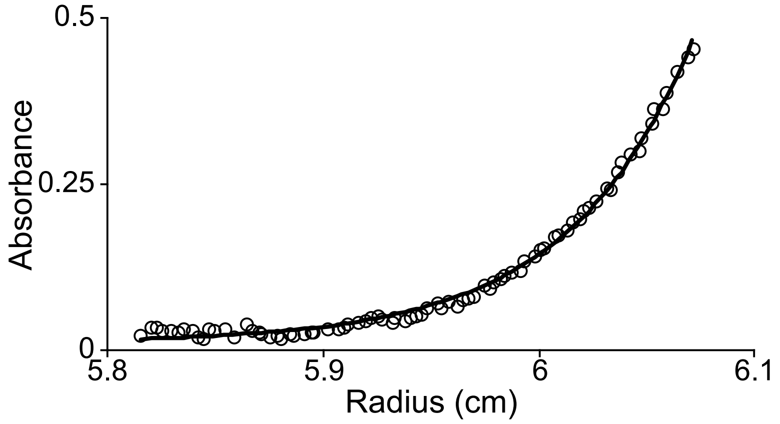

b

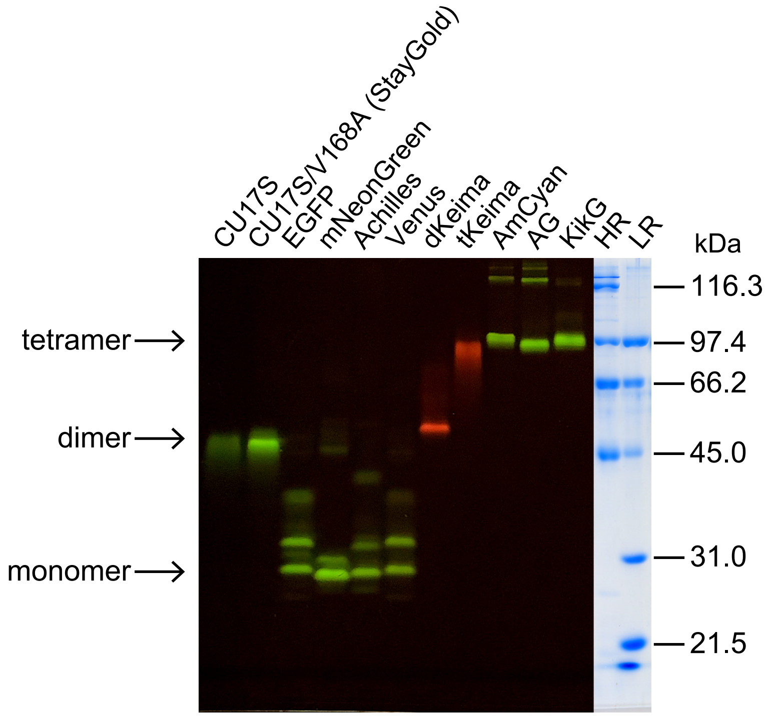

Supplementary Fig. 2 | Structural characteristics of CU17S/V168A (StayGold).

a, The equilibrium radial absorbance profile at 20,000 rev./min by analytical ultracentrifugation analysis for purified CU17S/V168A. The absolute molecular mass was determined to be 59.9 kDa, which was about twice larger than the value (28.3 kDa) deduced from the primary structure of this FP with a His-tag. Data are derived from single measurements. A similar determination (59.5 kDa) was obtained from another independent experiment. **b,** Pseudonative SDS/PAGE of purified CU17S and CU17S/V168A (StayGold). EGFP, mNeonGreen, Achilles, and Venus were analyzed as reference monomers. Dimeric-Keima (dKeima) was analyzed as a reference dimer. Tetrameric-Keima (tKeima), AmCyan, Azami-Green (AG), and Kikume-Green (KikG) were analyzed as reference tetramers. HR: SDS/PAGE Molecular Weight Standards, High Range. RL: SDS/PAGE Molecular Weight Standards, Low Range. Shown is a representative of n = 4 experiments.

a

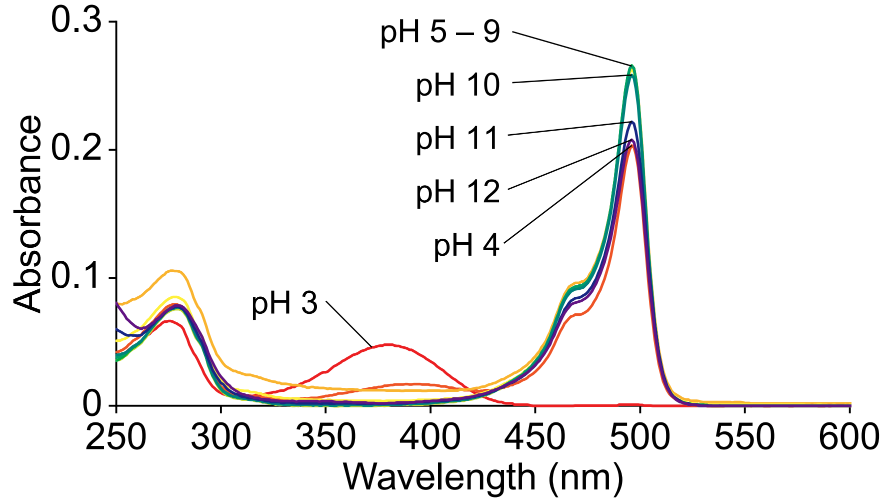

b

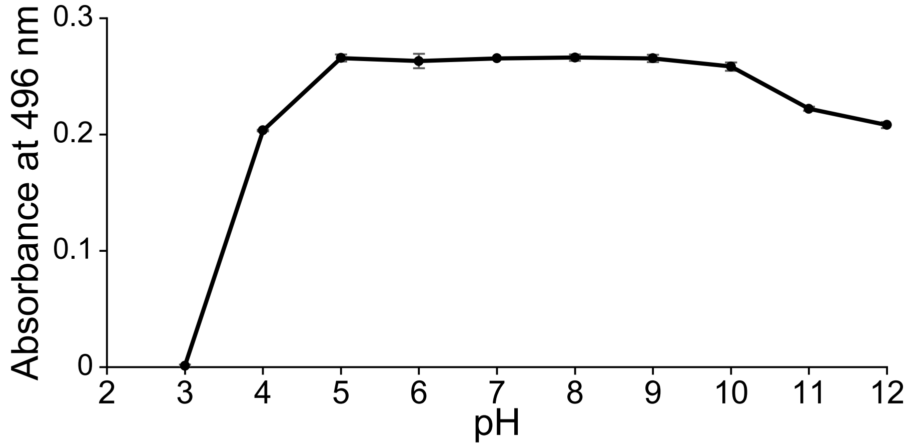

Supplementary Fig. 3 | pH titrations of CU17S/V168A (StayGold).
a, Absorbance spectra at different pHs. Shown is a representative of n = 3 experiments.
b, pH-dependence of the absorbance at 496 nm. Data points are shown as means ± SD (n = 3 independent experiments).

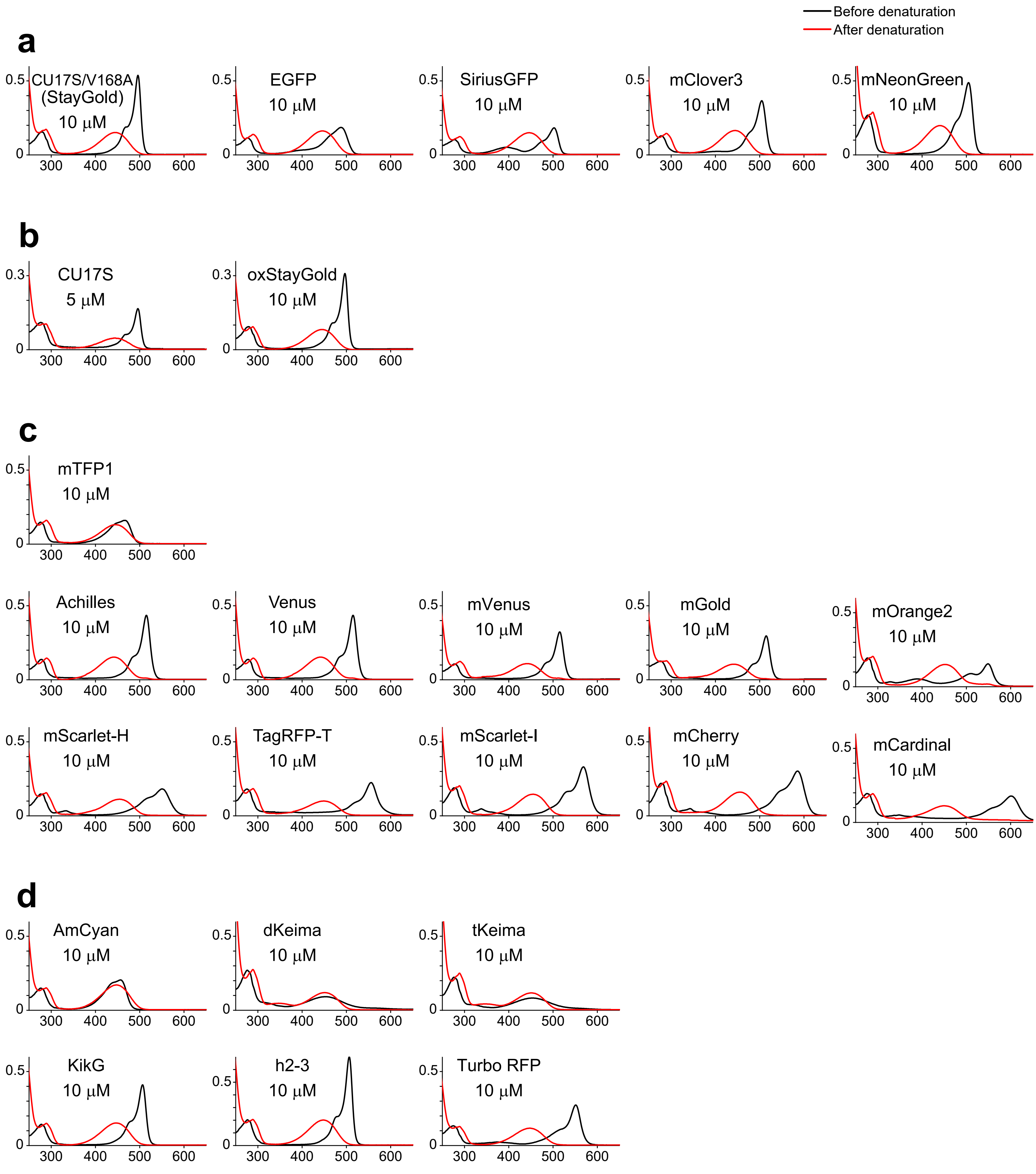

Supplementary Fig. 4 | Assessment of inherent qualities of FP chromophores.
Absorption spectra of FPs before (solid line) and after (red line) denaturation with 0.1 M NaOH. FP concentration was 5 μM or 10 μM. Path length was 1 cm. All these FPs carry X-Tyr-Gly, a chromophore-forming tripeptide, and their alkali-denatured chromophores contain a dehydrotyrosine residue conjugated to the imidazolone group and absorb light maximally at 447 nm with a molar extinction coefficient of 44,000 M^{-1} cm^{-1}. This value was used for the determination of their absolute molar extinction coefficients.
a, Green-emitting FPs including StayGold. Related to Fig. 1h, Table 1.
b, StayGold variants. Related to Fig. 2a, Supplementary Table 1a.
c, Various colored FPs. Related to Fig. 2b, Supplementary Table 1b.
d, Multimeric FPs. Related to Fig. 2c, Supplementary Table 1c.

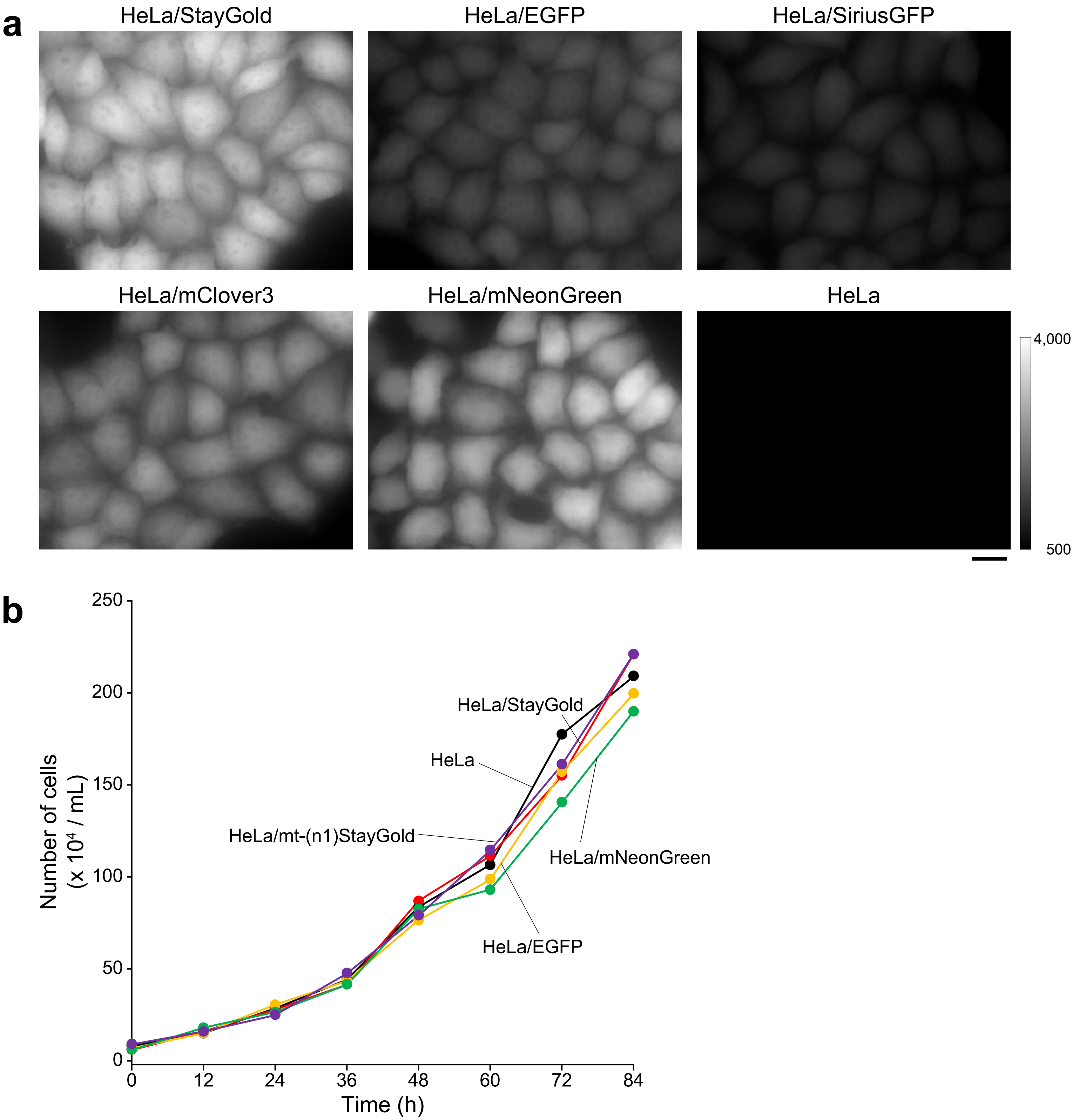

Supplementary Fig. 5 | HeLa cell stably expressing green-emitting FPs.

a, Representative low-magnification fluorescence images of HeLa cell populations stably expressing StayGold, EGFP, SiriusGFP, mClover3, or mNeonGreen. *right bottom,* control HeLa cells. All the images were acquired under the same conditions. Scale bar, 20 μm. The gray scale (500–4,000) indicates that the lowest and highest fluorescence intensities of the image are 500 and 4,000, respectively. Almost uniform fluorescence intensity among all the observed cells of each stable cell line was confirmed. HeLa/StayGold and HeLa/mNeonGreen cell lines were used for the experiment shown in Fig. 1j. HeLa/StayGold and HeLa/EGFP cell lines were used for the experiment shown in Fig. 1k.

b, Assessment of cytotoxicity of FP expressed in cultured HeLa cells. The number of cultured HeLa cells, HeLa/StayGold cells, HeLa/EGFP cells, or HeLa/mNeonGreen cells was plotted over time. Cell growth curves of StayGold-, EGFP-, and mNeonGreen-expressing HeLa cell lines are shown together with that of control HeLa cells. No growth retardation was found in these FP-expressing cell lines. It was thus concluded that StayGold, as well as EGFP and mNeonGreen, can label cells without cytotoxicity. In addition, the growth of HeLa/mt-(n1)StayGold cells was assessed together (Supplementary Fig. 12b, Extended Data Fig. 3). Data are derived from single measurements using a hematocytometer (n = 1).

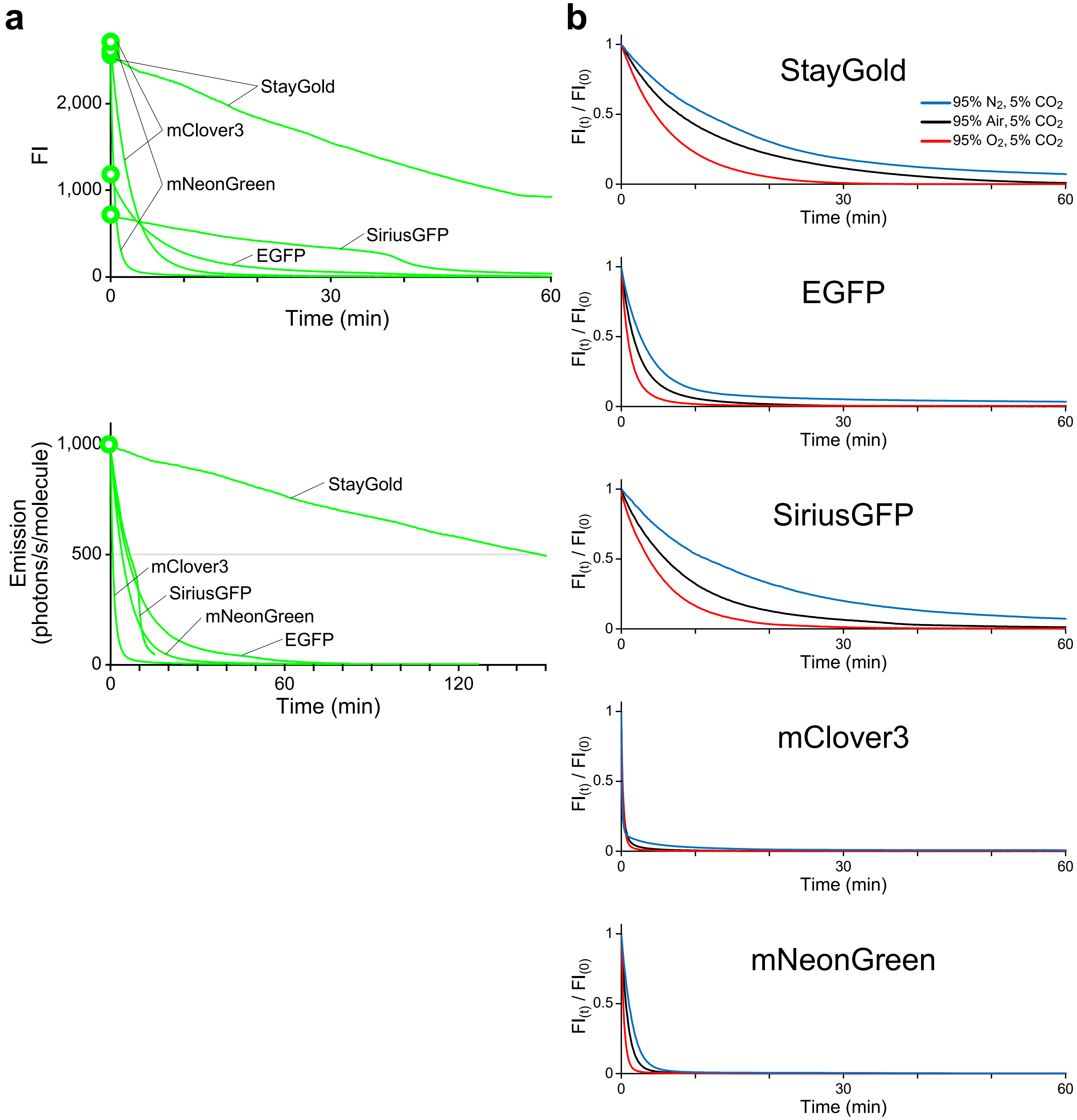

Supplementary Fig. 6 | Oxygen sensitivity of photobleaching of green-emitting FPs.
Photostability of StayGold, EGFP, SiriusGFP, mClover3, and mNeonGreen in living HeLa cells cultured in growth medium (DMEM without phenol red).
a, Photobleaching curves for StayGold, EGFP, SiriusGFP, mClover3, and mNeonGreen under normoxic condition (95% air, 5% CO_2). The curves are representative of 4 repetitions (n = 4 independent transfections).
top, Measured fluorescence intensities were plotted over time.
bottom, Normalized bleaching curves. The time axis was normalized against an initial photon emission rate of 1,000 photons/s/molecule. Related to Table 1. A dry 40× objective lens (UPlanSApo 40×/0.95 NA) was used to excite all cells with intense blue light (488 ± 10 nm, 5.6 W/cm^2).
b, Comparison of photobleaching curves for each green-emitting FP in the presence of 95% air and 5% CO_2 (normoxic condition, black line), 95% N_2 and 5% CO_2 (anoxic condition, blue line), and 95% O_2 and 5% CO_2 (hyperoxic condition, red line). An oil immersion 60× objective lens (UPlanSApo 60×/1.35 NA) was used.

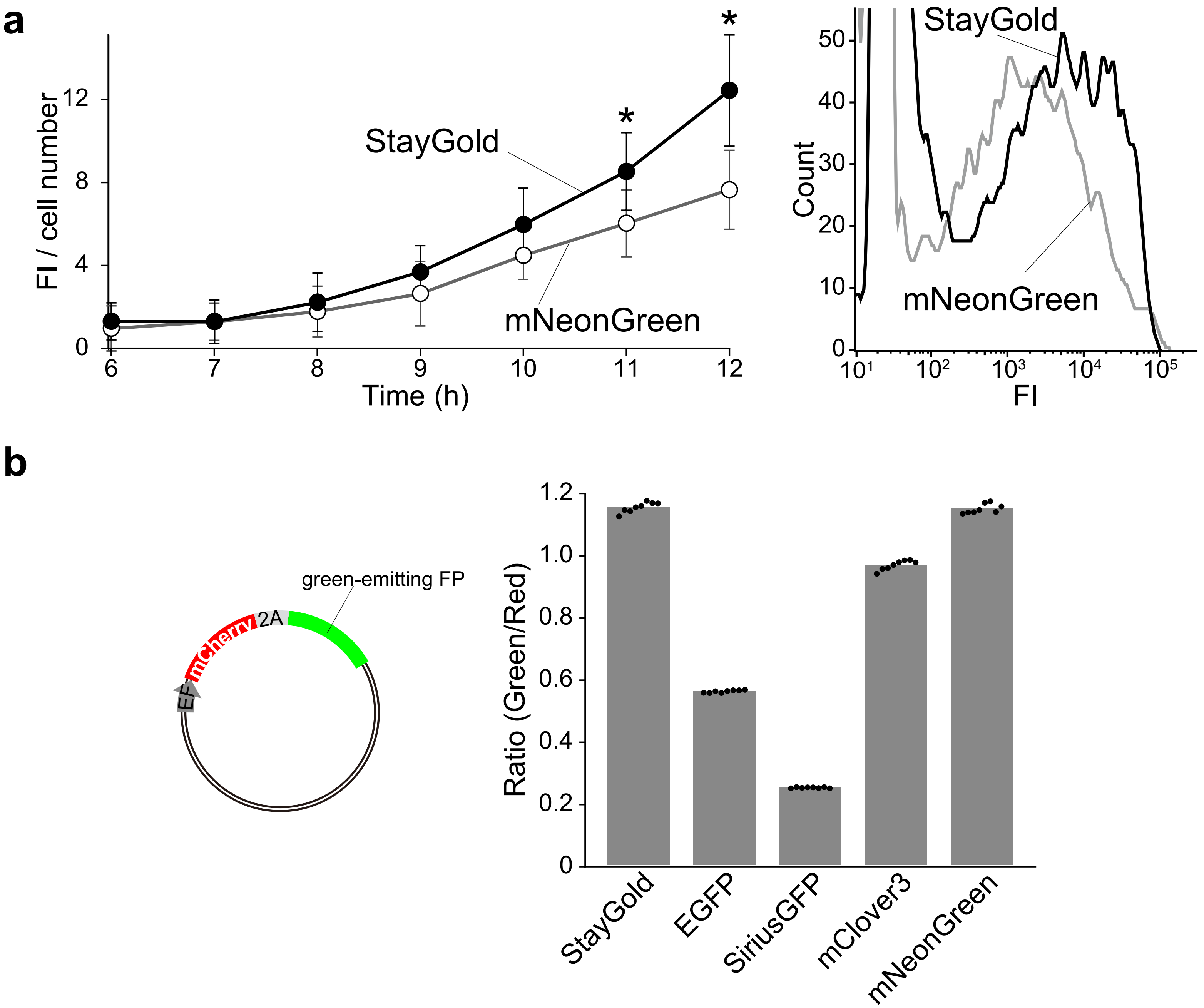

Supplementary Fig. 7 | Development of fluorescence of green-emitting FPs in HeLa cells after cDNA transfection.

a, StayGold was compared with mNeonGreen, which is a very rapidly-maturing FP.

left, Fluorescence development in HeLa cells in an early phase (6–12 h) after transfection with cDNAs of StayGold (solid circles) or mNeonGreen (open circles). Fluorescence intensity was normalized for cell number. Data points are shown as means ± SEM (n = 12 fields from three different experiments). Statistical significance (*$P < 0.01$) was examined by Welch's unpaired two-sided t test. $P = 0.00294$ (11 h). $P = 0.000107$ (12 h).

right, Flow cytometry analysis performed on HeLa cells 72 h after transfection with cDNAs encoding StayGold (black line) and mNeonGreen (grey line). The traces are representative of 2 repetitions (n = 2 independent experiments).

b, Cellular fluorescence brightness of five green-emitting FPs in HeLa cells 30 h after cDNA transfection. Normalized to mCherry fluorescence.

Cotranslation of green-emitting FP with mCherry using the bicistronic coexpression system. Transfection was performed with pCSII-EF/mCherry-T2A-green-emitting FP. The green/red ratio values were plotted. Statistically, they were 1.151 ± 0.016, 0.559 ± 0.004, 0.250 ± 0.001, 0.965 ± 0.016, and 1.146 ± 0.015 (means ± SD, n = 8 different wells) for StayGold, EGFP, SiriusGFP, mClover3, and mNeonGreen, respectively. These values were normalized to that of EGFP and are shown in Table 1 (cellular brightness).

		Source	E. coli (FI)	HeLa cell (PM targeting)
**N-terminus**				
StayGold	MASTPFKFQL...		○	△
(n1)StayGold	MAST**GEELFTGVV**PFKFQL...	EGFP(5–13)	○	○
(n2)StayGold	MAS**KGEELFTGV**TPFKFQL...	EGFP(4–12)	○	○
(n3)StayGold	MAST**IKEFM**PFKFQL...	DsRed(8–12)	△	–
(n4)StayGold	MASTP**NVIKEFMR**FKFQL...	DsRed(6–13)	△	–
(n5)StayGold	MAS**SKNVIKEF**TPFKFQL...	DsRed(4–11)	○	△
(n6)StayGold	MASTPFKF**PMTSKI**QL...	dfGFP(11–16)	×	–
(n7)StayGold	MASTP**ALFQYPMT**FKFQL...	dfGFP(6–13)	△	–
(n8)StayGold	MAS**GRALFQYP**TPFKFQL...	dfGFP(4–11)	○	△
**C-terminus**				
StayGold	...QSETLEAHL		○	△
StayGold(c1)	...QSETLEAHL**ITLGMDELYK**	EGFP(230–239)	×	–
StayGold(c2)	...QSETLEAHL**HLFL**	DsRed(222–225)	×	–
StayGold(c3)	...QSETLEA**GNPWHEPSASAV**	dfGFP(221–233)	×	–
StayGold(c4)	...QSETLEAHL**PWHEPSASAV**	dfGFP(223–233)	△	○
StayGold(c5)	...QSETLEA**VDLDTYQ**	obeliaCFP(228–234)	×	–
StayGold(c6)	...QSETLEAHL**LDTYQ**	obeliaCFP(230–234)	×	–

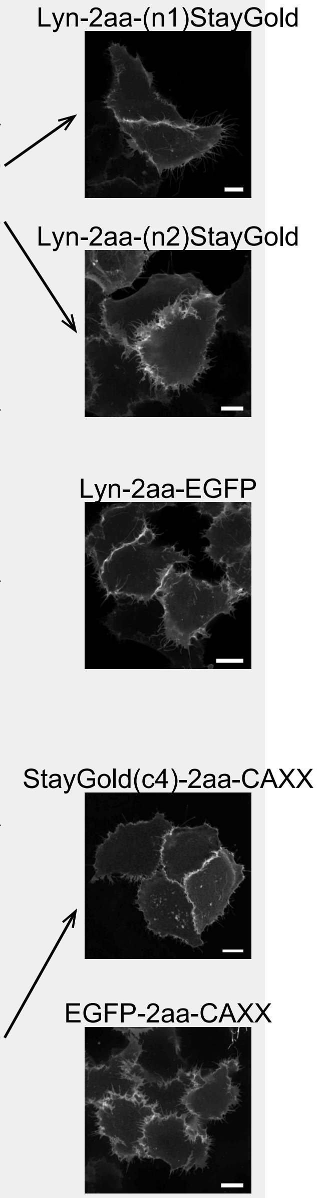

Supplementary Fig. 8 | Extension of N- and C-termini of StayGold for fusion applications.
For functional fusion to plasma membrane (PM)-targeting signals, amino acid residues of other FPs (indicated in red) were transplanted into the N- and C-termini of StayGold. Fluorescence development of the resulting constructs was examined in their bacterial colonies. FI: fluorescence intensity. The constructs that produced bright and dim colonies (open circles and triangles, respectively) were selected for PM-targeting experiments using cultured HeLa cells.
top, StayGold, (n1)StayGold, (n2)StayGold, (n3)StayGold, (n4)StayGold, (n5)StayGold, (n7)StayGold, and (n8)StayGold were fused to the 22 amino acid N-terminus of a receptor tyrosine kinase (Lyn). Among them, (n1)StayGold and (n2)StayGold were found to label the plasma membrane homogenously. Typical fluorescence images of HeLa cells expressing Lyn-2aa-(n1)StayGold, Lyn-2aa-(n2)StayGold, and Lyn-2aa-EGFP are shown. Scale bars, 10 μm.
bottom, StayGold and StayGold(c4) were fused to the 20 amino acid C-terminus of K-Ras. In spite of the dim fluorescence in bacterial colonies, StayGold(c4) provided better labeling of the plasma membrane than StayGold. Typical fluorescence images of HeLa cells expressing StayGold(c4)-2aa-CAAX and EGFP-2aa-CAAX are shown. Scale bars, 10 μm.
Brightness of colony fluorescence was judged in single experiments (n = 1). The PM-targeting images are representative of 3 repetitions (n = 3 independent transfections).

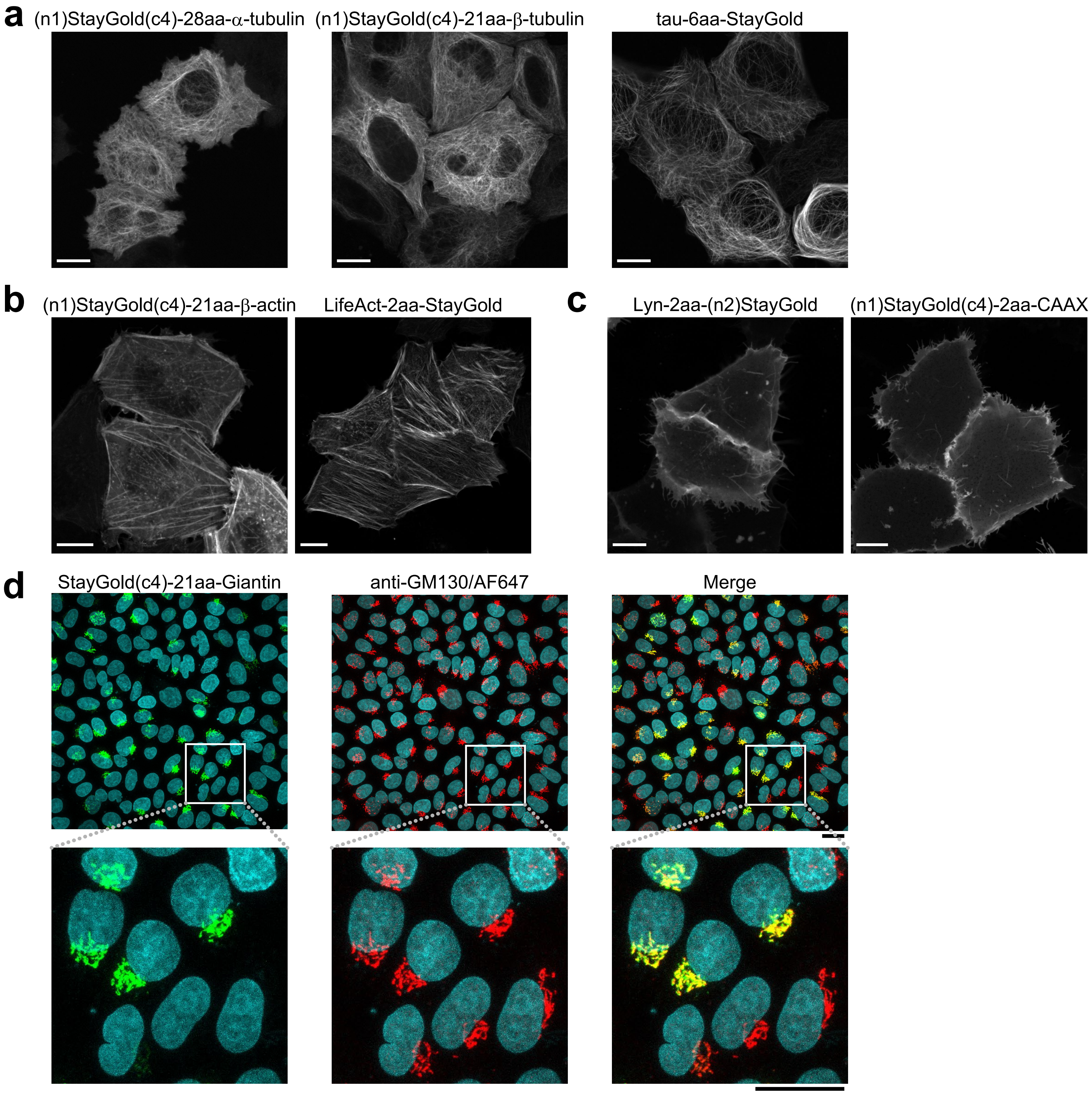

Supplementary Fig. 9 | Fluorescence imaging of StayGold subcellular targeting fusions.

a, Microtubule localization.

b, Filamentous actin localization.

c, Plasma membrane localization. Lyn: 22 N-terminal amino acids of the nonreceptor tyrosine kinase. CAAX: 20 C-terminal amino acids of K-Ras.

a–c, Confocal images (single planes) of the localization in living HeLa cells. Scale bars, 10 μm.

d, Golgi localization. Amino acids 3,131–3,259 of human Giantin were used to target the Golgi apparatus. Nuclei were counterstained with DAPI (cyan). Confocal images (Maximum Intensity Projection) of StayGold (green), anti-GM130 immunostaining (red) and DAPI (cyan) in fixed HeLa cells. Complete match between the green and red signals indicates the correct targeting of StayGold(c4)-21aa-Giantin.

In addition, no difference in Golgi morphology was observed between transfected and non-transfected cells, suggesting that StayGold(c4)-21aa-Giantin had no adverse effect. Scale bars, 20 μm.

a–d, HeLa cells were transfected with plasmids encoding constructs fused to StayGold, StayGold(c4), (n1)StayGold(c4), or (n2)StayGold (See Supplementary Fig. 8). Number indicates the linker amino acid length. The images are representative of 3 repetitions (n = 3 independent experiments).

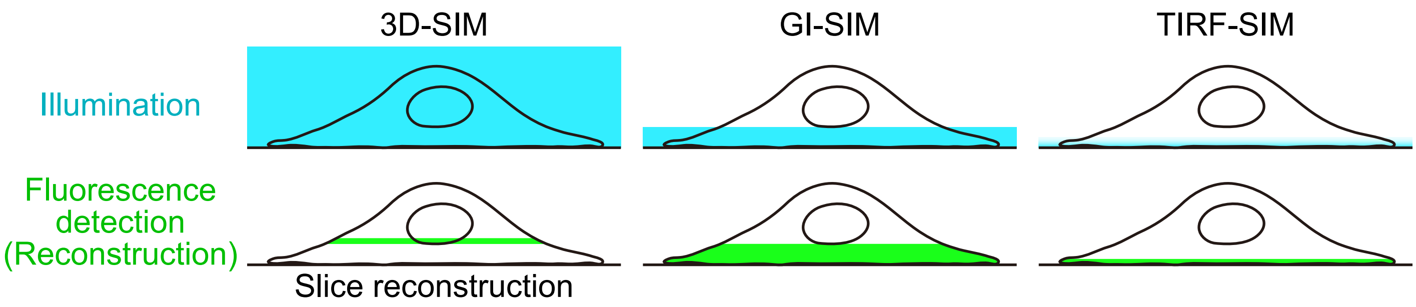

Supplementary Fig. 10 | Comparison of 3D-SIM, GI-SIM, and TIRF-SIM.
The upper panel illustrates the illumination depth of the three SIM modalities (ref. 9). Shaded are temporally averaged illumination patterns; 2D or 3D interference pattern is created at each time point.
The lower panel illustrates the localization of detected emitters after reconstruction. Slice reconstruction of 3D-SIM allows axial super-resolution imaging with optical sectioning at an arbitrary *z*-position.

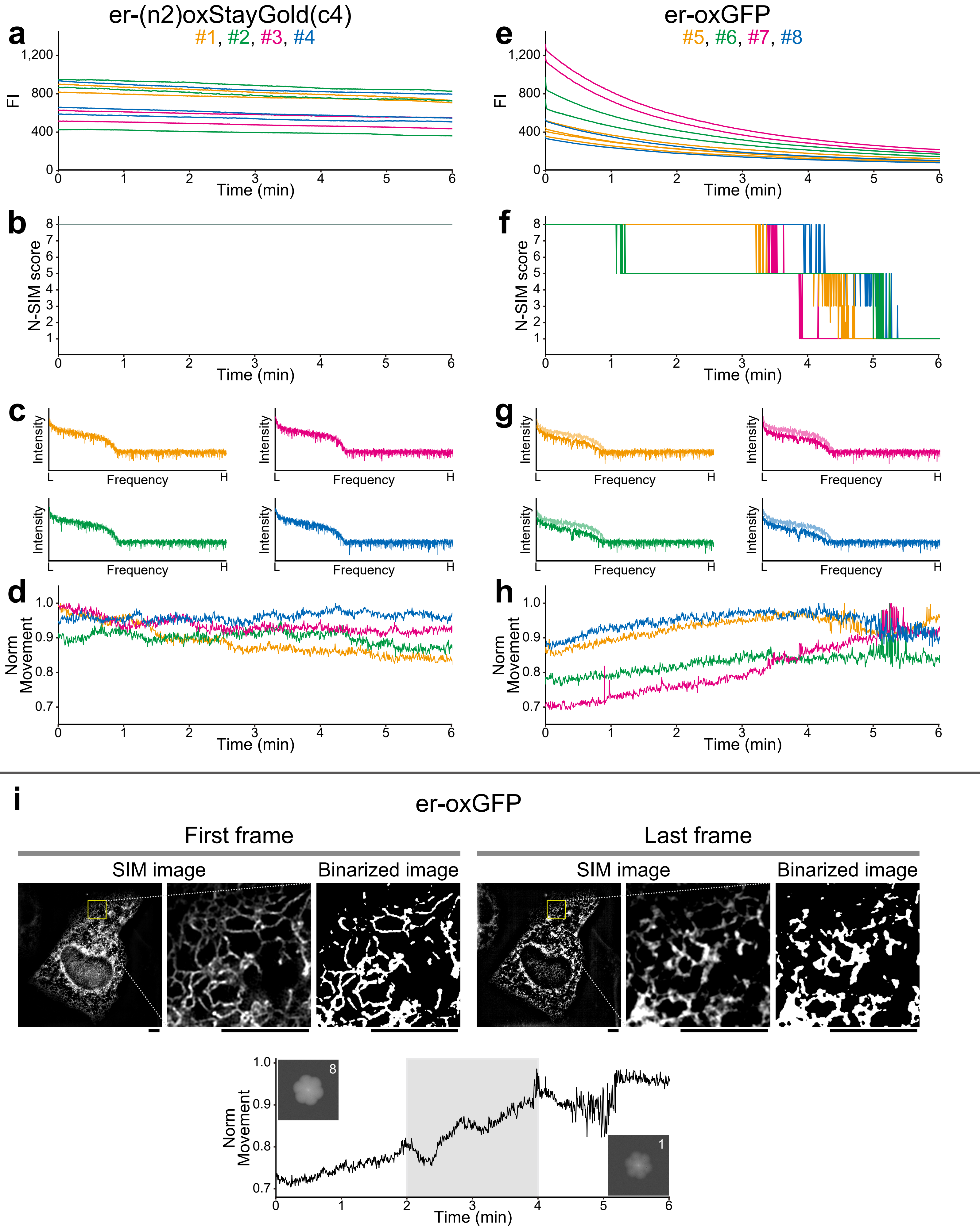

Supplementary Fig. 11 | High photostability of ER marker is essential to ER dynamics observation by 3D-SIM (Figures 4a–d).

Supplementary Fig. 11 | High photostability of ER marker is essential to ER dynamics observation by 3D-SIM (Figures 4a–d).

a–h, Comparison of er-(n2)oxStayGold(c4) and er-oxGFP in similar 3D-SIM experiments (2.4 W/cm^2, 3.1 frames/s, 6 min) but with no Ca^{2+} mobilization.

a–d, Four independent experiments that used HeLa cells expressing er-(n2)oxStayGold(c4). Two, three, two, and three cells were observed in experiment #1 (yellow), experiment #2 (green), experiment #3 (red), and experiment #4 (blue), respectively.

e–h, Four independent experiments that used HeLa cells expressing er-oxGFP. Four, two, two, and two cells were observed in experiment #5 (yellow), experiment #6 (green), experiment #7 (red), and experiment #8 (blue), respectively.

a, e, Averaged fluorescence intensities (FIs) in individual cells were plotted against time.

b–d, f–h, The full image of each experiment was analyzed.

b, f, Temporal profiles of the SIM image quality index. The N-SIM score ranges between 8 (good) and 1 (bad).

c, g, FFT spectra of the first and last images (light and dark lines, respectively) from the reconstructed datasets.

d, h, Temporal profiles of ER movement.

i, A 3D-SIM experiment (2.4 W/cm^2, 3.1 frames/s, 6 min) using a HeLa cell expressing er-oxGFP with transient Ca^{2+} mobilization. SIM and binarized images at t = 0 min (left) and t = 6 min (right). In the last frame, apparent fragmentation and thickening of the ER tubules are evident, indicative of the deterioration of SIM image quality. Compare with Extended Data Fig. 2a. The cell was challenged with a histamine (0.1 mM) at 2 min and an anti-histamine reagent (10 μM) at 4 min; the time zone of Ca^{2+} mobilization is shaded. The calculated ER movement fluctuated greatly, its dependency on Ca^{2+} mobilization could not be well characterized. 2D FFT images at t = 0 and 6 min are shown with their N-SIM scores. Scale bars, 5 μm.

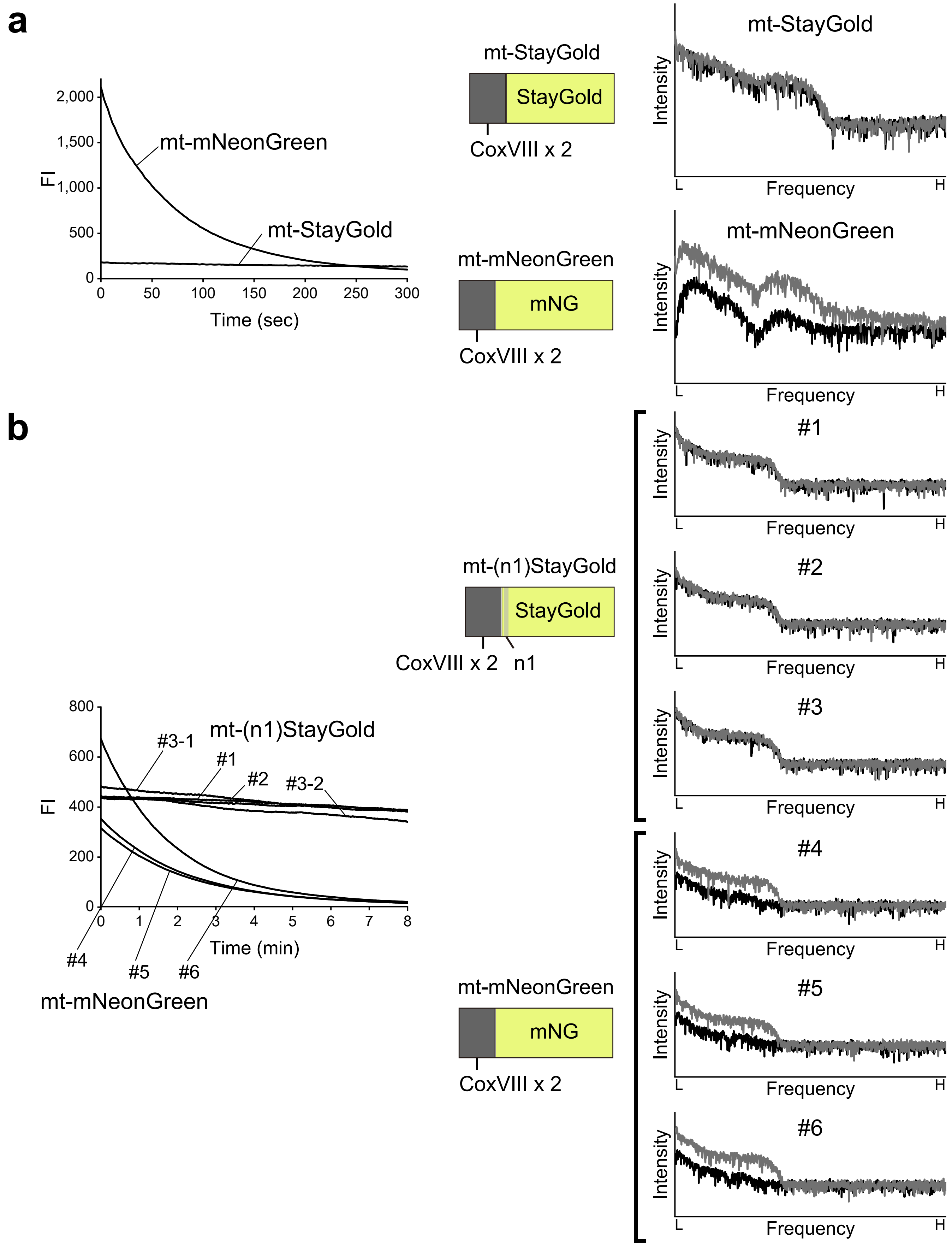

Supplementary Fig. 12 | Comparison of StayGold and mNeonGreen for continuous and long-term imaging of mitochondria by 3D-SIM.

After cDNA transfection, HeLa cells expressing mitochondrially-targeted FPs were imaged by 3D-SIM. mNG: mNeonGreen. FI: fluorescence intensity. mt-(n1)StayGold provides brighter mitochondrial labeling than mt-StayGold.

left, The averaged fluorescence intensities of individual cells are plotted against time.

right, FFT spectra of the first and last images (grey and black lines, respectively) from the reconstructed data set.

a, HeLa cells expressing mt-StayGold or mt-mNeonGreen were imaged continuously at 0.72 frames/s for 5 min. Illumination intensity: 3.4 W/cm^2. See Supplementary Video 5 for the performance of mt-StayGold.

b, HeLa cells expressing mt-(n1)StayGold or mt-mNeonGreen were imaged continuously at 3.1 frames/s for 8 min. Illumination intensity: 2.4 W/cm^2. Six (3 × 2) independent experiments were performed. Experiment #3 observed two cells.

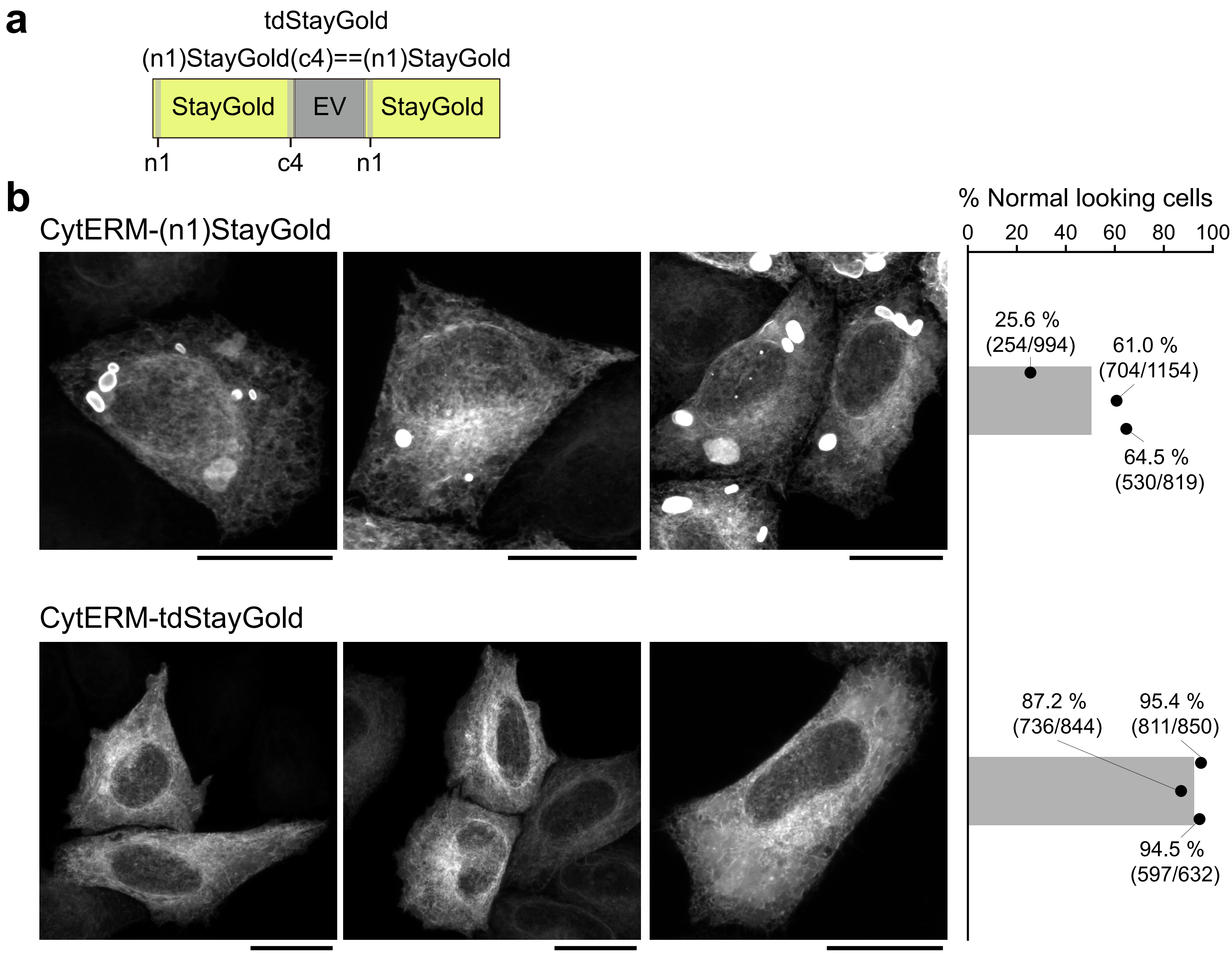

Supplementary Fig. 13 | StayGold tandem dimer (tdStayGold).

a, Domain structure of tdStayGold. ==: EV linker, a 29 repeat of the amino acid linker Ser-Ala-Gly-Gly.

b, OSER assay for assessment of oligomeric state of StayGold vs. tdStayGold. HeLa cells were transfected with constructs encoding CytERM-(n1)StayGold (top) or CytERM-tdStayGold (bottom). Transfection was repeated three times, and one representative image (confocal *z*-stack projection) was generated from each of the transfection experiments. Scale bars, 20 µm. Percentage of cells scored without visible whorl structures is summarized on the right. The mean scores are shown by grey bars.

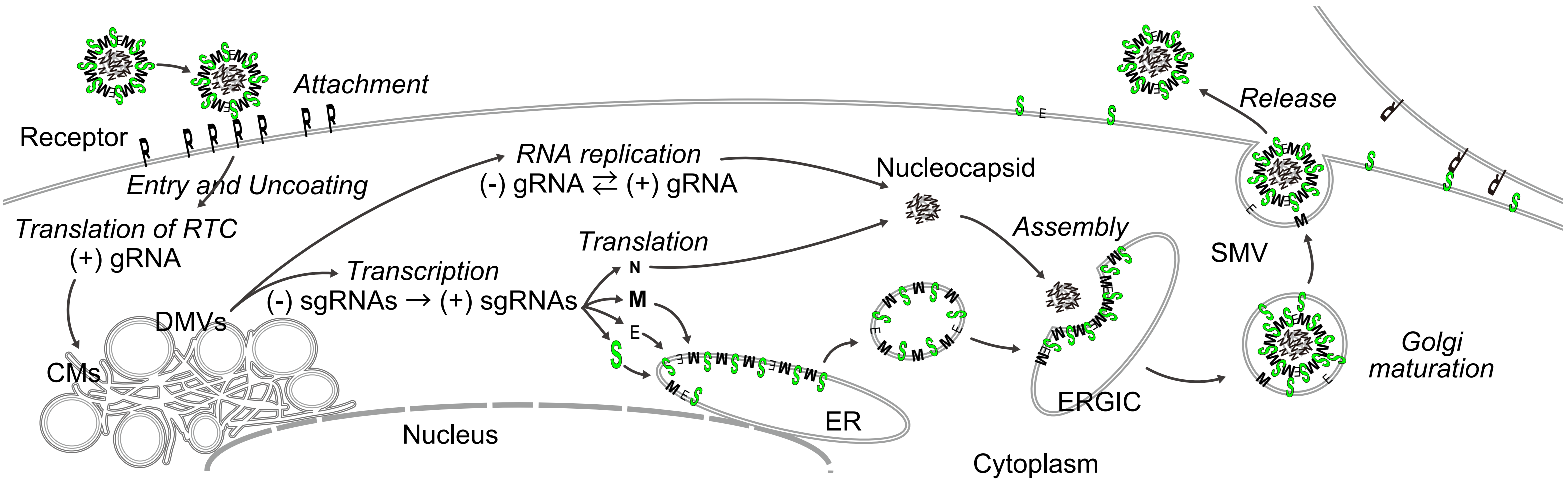

Supplementary Fig. 14 | The replication cycle of SARS-CoV-2.
This schema detains events surrounding viral assembly (ref. 10, 11). Nucleocapsid, spike, envelope, and membrane structural proteins are indicated by their initial letters: N, S, E, and M, respectively. S is highlighted in green. RTC: replication transcription complex. gRNA: genomic RNA. sgRNA: sub-genomic RNA. Event names are italicized.

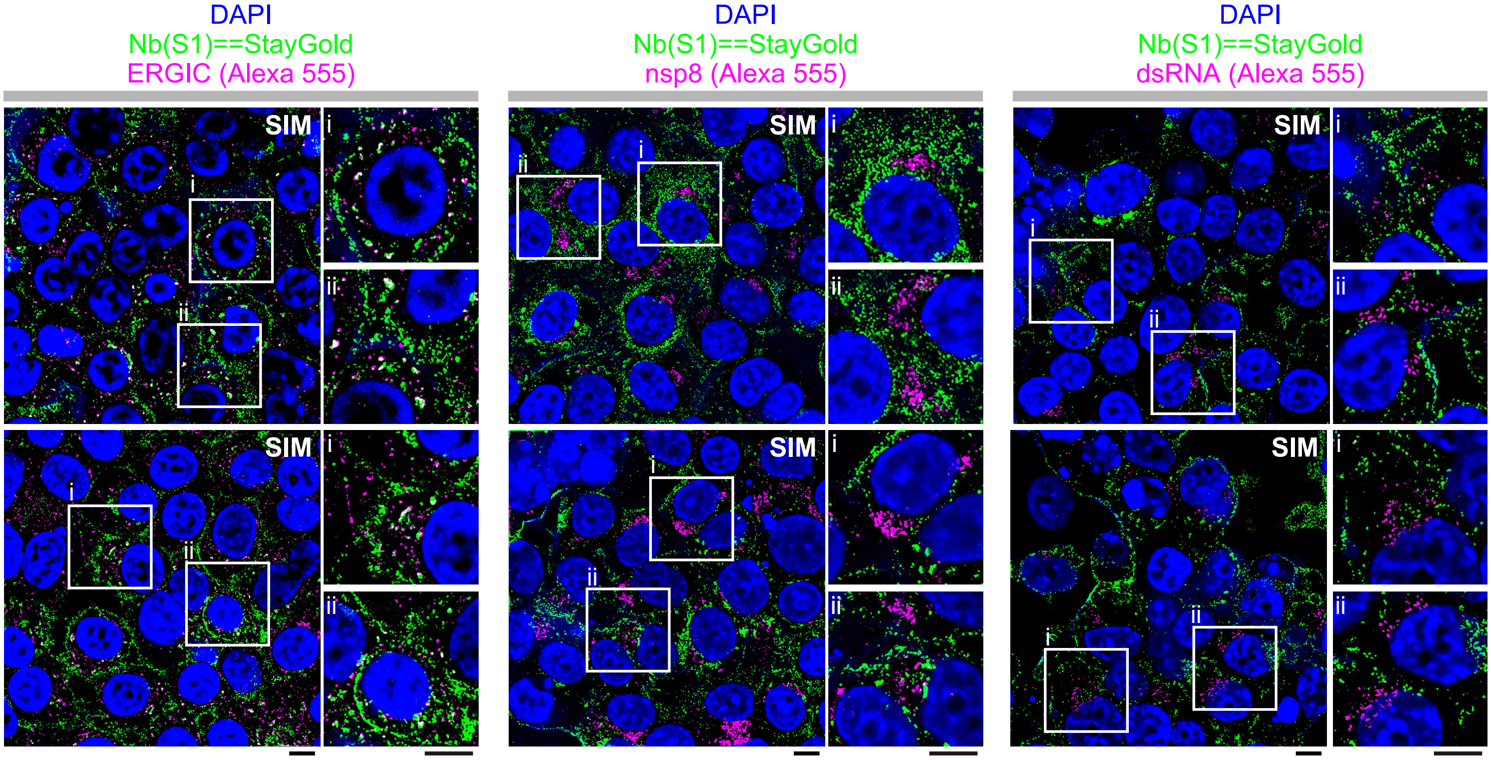

Supplementary Fig. 15 | Dual-color 3D-SIM images of SARS-CoV-2-infected VeroE6/TMPRSS2 cells.
left, Two *xy* image datasets similar to Fig. 5i but from different volumetric imaging experiments.
Single-plane 3D-SIM images of SARS-CoV-2 spike protein (green) and ERGIC3 (magenta).
middle, Two *xy* image datasets similar to Fig. 5j but from different volumetric imaging experiments.
Single-plane 3D-SIM images of SARS-CoV-2 spike protein (green) and nsp8 (magenta).
right, Two *xy* image datasets similar to Fig. 5k but from different volumetric imaging experiments.
Single-plane 3D-SIM images of SARS-CoV-2 spike protein (green) and dsRNA (magenta).
Nuclear (blue) images are sectional but not SIM-reconstructed. Areas enclosed by white boxes are enlarged on the right. These data indicate the reproducibility of the finding that S signals overlapped ERGIC but not nsp8 or dsRNA signals. Scale bars, 5 µm.
The data shown are from single samples per staining condition.

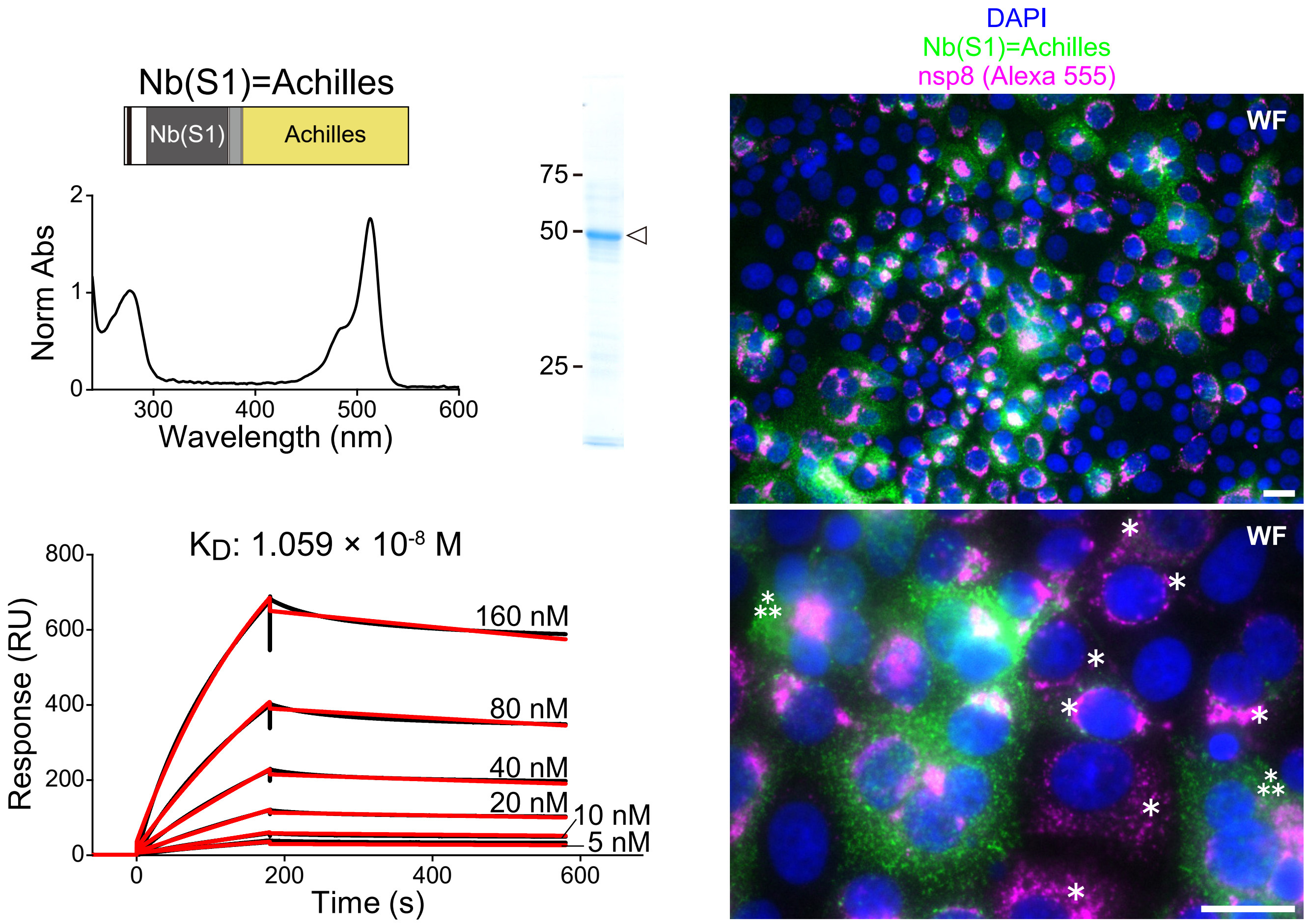

Supplementary Fig. 16 | Visualizing SARS-CoV-2 assembly using Nb(S1)=Achilles.
upper left, Domain structure of Nb(S1)=Achilles. =: Coupler linker, a triple repeat of the amino acid linker Gly-Gly-Gly-Gly-Ser (indicated by a gray bar in the schema). Absorption spectrum of Nb(S1)=Achilles. Normalized to the peak at 280 nm. Coomassie Brilliant Blue staining for the visualization of Nb(S1)=Achilles separated by SDS-PAGE. The molecular weight of Nb(S1)=Achilles is 46 kDa.
lower left, SPR experiment of S1 binding to immobilized Nb(S1)=Achilles. Black traces show raw data; red lines show kinetic fit.
right, VeroE6/TMPRSS2 cells infected with SARS-CoV-2 (MOI 0.5, 24 hpi). Spike protein (green). nsp8 (magenta). Nuclei were counterstained with DAPI (blue). WF: wide-field microscopy observation. In a low magnification micrograph (top), it is possible to distinguish infected cells from uninfected ones. In a high magnification micrograph (bottom), cells stained exclusively for nsp8 are labeled with asterisks; these cells should presumably be in an initial stage of infection. In contrast, cells filled with S signals as well are labeled with asterisms; they should be in a later stage of infection as evidenced by their frequent formation of syncytia. Since such yellow-emitting fluorescent proteins as Achilles are not sufficiently photostable for 3D-SIM (ref. 1), samples reacted with Nb(S1)=Achilles were imaged only by wide-field (WF) microscopy. Scale bars, 20 µm.

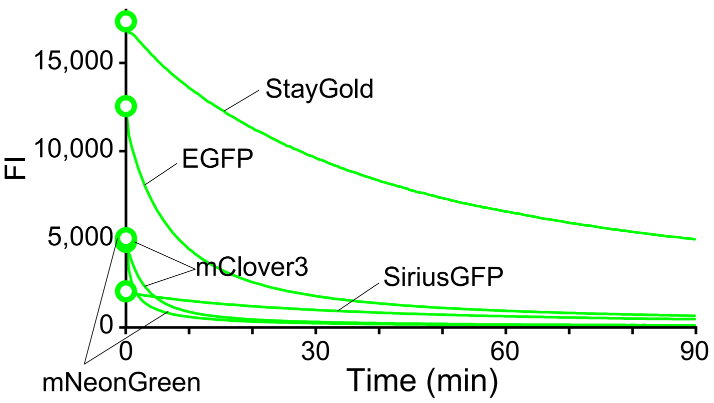

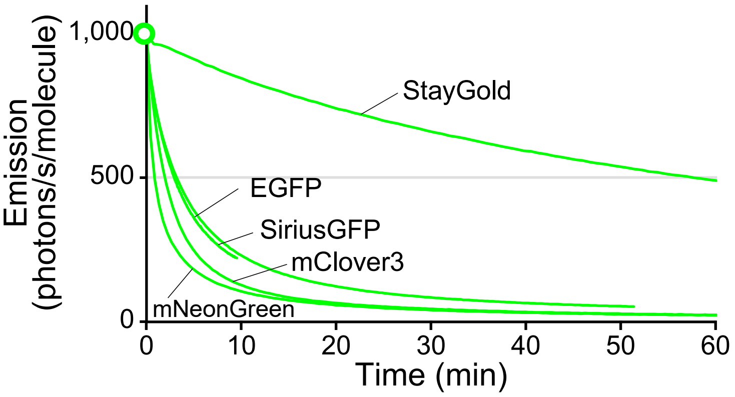

Supplementary Fig. 17 | Photostability of purified green-emitting FPs (1 μM in polyacrylamide gel) under continuous illumination by spinning disk confocal microscopy.
Illumination intensity: 1.7 W/cm^2. Plotted as measured intensity vs. time (top) or as intensity vs. normalized total exposure time with an initial emission rate of 1,000 photons/s/molecule (bottom). The calculated $t_{1/2}$ values were: 3,470 sec (StayGold), 206 sec (EGFP), 179 sec (SiriusGFP), 127 sec (mClover3), and 60 sec (mNeonGreen). The curves shown are representative of 2 repetitions (n = 2 independent experiments).

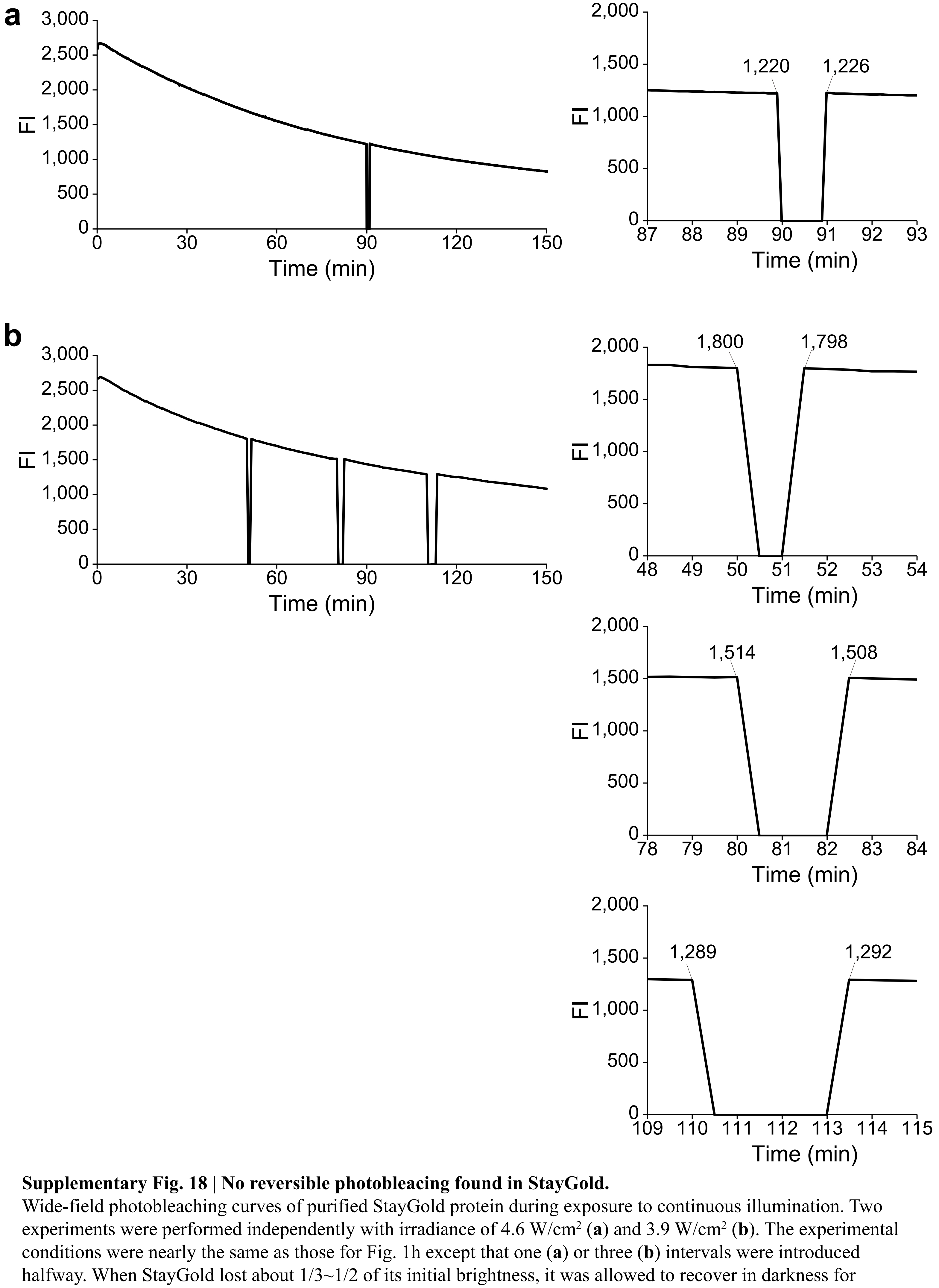

Supplementary Fig. 18 | No reversible photobleacing found in StayGold.

Wide-field photobleaching curves of purified StayGold protein during exposure to continuous illumination. Two experiments were performed independently with irradiance of 4.6 W/cm^2 (**a**) and 3.9 W/cm^2 (**b**). The experimental conditions were nearly the same as those for Fig. 1h except that one (**a**) or three (**b**) intervals were introduced halfway. When StayGold lost about 1/3~1/2 of its initial brightness, it was allowed to recover in darkness for 1 min (**a**) or 1, 2, and 3 min (**b**). However, no substantial recovery was observed.

a

Excitation[a] (nm)	Irradiance[b] (W/cm^2)	Photon flux[c] ($photons/s/cm^2$)	FP[d]	λab[e] (nm)	λem[f] (nm)	ε[g] ($10^3\ M^{-1}\ cm^{-1}$)		QY$_f$[h]	Photostability $t_{1/2}$ (sec)[i]
						λab	488 nm		
488	5.6	1.38×10^{19}	StayGold	496	505	159	105	0.93	11,487
			CU17S	496	505	164	111	0.90	10,145
			oxStayGold	497	506	169	108	0.93	11,784
			tdStayGold	496	504	162	108	0.90	11,361

b

Excitation[a] (nm)	Irradiance[b] (W/cm^2)	Photon flux[c] ($photons/s/cm^2$)	FP[d]	λab[e] (nm)	λem[f] (nm)	ε[g] ($10^3\ M^{-1}\ cm^{-1}$)		QY$_f$[h]	Photostability $t_{1/2}$ (sec)[i]
						λab	Excitation wavelength		
435.8	4.1	9.02×10^{18}	mTFP1	466	492	54	38	0.79	805
488	5.6	1.38×10^{19}	StayGold	496	505	159	105	0.93	11,487
			EGFP	488	509	51	51	0.71	701
			SiriusGFP	502	516	54	35	0.19	477
			mClover3	505	518	99	52	0.84	289
			mNeonGreen	505	518	112	64	0.87	176
514.5	3.4	8.83×10^{18}	Achilles	513	525	144	141	0.76	584
			Venus	515	528	128	128	0.69	302
	1.16	3.00×10^{18}	mVenus	515	528	135	135	0.69	352
			mGold	515	528	129	129	0.70	865
550	3.4	9.44×10^{18}	mOrange2	549	565	46	45	0.49	1,178
			mScarlet-H	550	592	74	74	0.22	1,273
			TagRFP-T	556	585	101	92	0.40	994
			mScarlet-I	569	593	104	62	0.51	459
			mCherry	585	610	86	49	0.21	485
580	4.6	1.35×10^{19}	mCardinal	601	659	70	50	0.20	350

c

Excitation[a] (nm)	Irradiance[b] (W/cm^2)	Photon flux[c] ($photons/s/cm^2$)	FP[d]	Multiplicity	λab[e] (nm)	λem[f] (nm)	ε[g] ($10^3\ M^{-1}\ cm^{-1}$)		QY$_f$[h]	Photostability $t_{1/2}$ (sec)[i]
							λab	Excitation wavelength		
435.8	4.1	9.02×10^{18}	AmCyan	4	456	486	52.9	46.6	0.88	1,228
			dKeima	2	453	616	33.8	31.1	0.29	593
			tKeima	4	453	616	30.9	28.2	0.16	289
488	5.4	1.33×10^{19}	KikG	4	506	517	119.9	54.3	0.90	384
			h2-3	2	506	516	154.3	57.9	0.90	650
550	3.4	9.44×10^{18}	Turbo RFP	2	551	574	104.1	103.7	0.78	626

Supplementary Table 1 | Characteristics of purified FPs.

Supplementary Table 1 | Characteristics of purified FPs.
a, StayGold and its variants. See Fig. 2a.
b, Various colored FPs. Purified protein data from Table 1 for green-emitting FPs, including StayGold, are incorporated here. See Fig. 2b.
c, Multimeric FPs. See Fig. 2c.
[a] Center wavelength of the illumination. [b] Power density of illumination from a 40× objective lens (UPlanSApo 40×/0.95 NA) (see Methods). [c] Calculated from the excitation wavelength and irradiance. [d] Fluorescent protein. [e] Absorbance maximum. [f] Emission maximum. [g] Absolute extinction coefficient at λab (left) and 488 nm (right). The measurement was based on the fact that after alkali denaturation of these FPs, the chromophore, containing a dehydrotyrosine residue conjugated to the imidazolone group, absorbs light maximally at 447 nm with a molar extinction coefficient of 44,000 M^{-1} cm^{-1}. See Supplementary Fig. 4. [h] Fluorescence quantum yield measured using an absolute photoluminescence quantum yield spectrometer. [i] Time in seconds required to reduce emission rate from 1,000 to 500 photons/s/molecule under wide-field illumination. All values were measured in this study.

Supplementary Video captions

Supplementary Video 1 | Direct photostability comparison between StayGold and mNeonGreen or EGFP in living HeLa cells, in a single field of view of wide-field microscopy.

First half: StayGold vs. mNeonGreen. See Fig. 1j.

Second half: StayGold vs. EGFP. See Fig. 1k.

Elapsed times (h: min: s).

Supplementary Video 2 | Visualizing rapid motion of ER tubules by using a new 3D-SIM technique that achieves nanoscale resolution on a millisecond time scale.

A COS cell expressing er-(n2)oxStayGold(c4) was imaged by lattice SIM (Elyra 7) continuously for 5.473 s. The total number of acquired frames was 736. Thus, the temporal resolution was 134.47 frames/s. The cell-wide field of view covered the ER network in the peripheral and perinuclear regions. This video (11.5 MB) has been generated via considerable compression of the original large-volume video data (8.98 GB). Compression was made using TMPGEnc. By minimizing the exposure time of each image (1 ms) based on the Burst mode in the lattice SIM system, it was possible for us to visualize ER dynamics at a temporal resolution of > 300 frames/s with similar image dataset quality. As SIM-based techniques enabling high-speed imaging of the ER, iSIM (instant SIM) (100 Hz) (ref. 12) and GI-SIM (266 Hz) (ref. 9) were previously reported, but their performance was assessed by use of ER markers that contained conventional *Aequorea* GFP variants. Again, it is expected that er-(n2)oxStayGold(c4) will be effectively combined with these techniques. See Extended Data Fig. 1.

Supplementary Video 3 | Agonist- and antagonist-induced longitudinal changes in ER structures revealed by fast, sustainable, cell-wide 3D-SIM imaging.

Three neighboring HeLa cells expressing er-(n2)oxStayGold(c4) were imaged by 3D-SIM (N-SIM S) continuously at a temporal resolution of 2.6 frames/s for 6 min. 10 μM histamine and 100 μM cyproheptadine were applied at 2 and 4 min, respectively. This video (14.3 MB) has been generated via considerable compression of the original large-volume video data (14.3 GB). Compression was made using TMPGEnc. Elapsed times (min: s). See Fig. 4a–d.

Supplementary Video 4 | Agonist- and antagonist-induced longitudinal changes in ER structures revealed by fast, sustainable, cell-wide 3D-SIM imaging.

Three neighboring HeLa cells expressing er-(n2)oxStayGold were imaged by 3D-SIM (N-SIM S) continuously at a temporal resolution of 1.1 frames/s for 6 min. 10 μM histamine and 100 μM cyproheptadine were applied at 2 and 4 min, respectively. This video (12.7 MB) has been generated via considerable compression of the original large-volume video data (6.10 GB). Compression was made using TMPGEnc. Elapsed times (min: s). See Fig. 4f–i.

Supplementary Video 5 | Sustainable and cell-wide 3D-SIM imaging for visualizing mitochondria.
A HeLa cell expressing mt-StayGold was imaged by 3D-SIM (N-SIM S) continuously at 0.72 frames/s for 5 min. left, Raw (left) and reconstructed (right) images are presented side-by-side. Elapsed times (min: s). See Supplementary Fig. 12a.

Supplementary Video 6 | Visualizing microtubule plus-end dynamics by sustainable wide-field microscopy imaging.
COS cells expressing EB3=tdStayGold were imaged continuously at 2 frames/s for 30 min. Elapsed times (min: s). See Extended Data Fig. 4a.

Supplementary References

1. Heintzmann, R. & Huser, T. Super-resolution structured illumination microscopy. *Chem. Rev.* **117,** 13890–13908 (2017).
2. Fukaya, M. & Watanabe, M. Improved immunohistochemical detection of postsynaptically located PSD-95/SAP90 protein family by protease section pretreatment: a study in the adult mouse brain. *J. Comp. Neurol.* **426,** 572–586 (2000).
3. El-Husseini, A.E. et al. PSD-95 involvement in maturation of excitatory synapses. *Science* **290,** 1364–1368 (2000).
4. Fortin, D.A. et al. Live imaging of endogenous PSD-95 using ENABLED: a conditional strategy to fluorescently label endogenous proteins. J. Neurosci. 34, 16698-16712 (2014).
5. Mikuni, T., Nishiyama, J., Sun, Y., Kamasawa, N. & Yasuda, R. High-throughput, high-resolution mapping of protein localization in mammalian brain by un vivo genome editing. *Cell* **165,** 1803–1817 (2016).
6. Shaner, N.C. et al. Improving the photostability of bright monomeric orange and red fluorescent proteins. *Nat. Methods* **5,** 545–551 (2008).
7. Linkert, M. et al. Metadata matters: access to image data in the real world. *J. Cell Biol.* **189,** 777–782 (2010).
8. Rigano, A. et al. Micro-Meta App: an interactive tool for collecting microscopy metadata based on community specifications. *Nat. Methods* **18,** 1489–1495 (2021).
9. Guo, Y. et al. Visualizing intracellular organelle and cytoskeletal interactions at nanoscale resolution on millisecond timescales. *Cell* **175,** 1430–1442 (2018).
10. Perlman, S. & Masters, P.S. *Emerging viruses: Fields virology*, P.M. Howley, D.M. Knipe, and S. Whelan, ed. (2020).
11. V'kovski, P., Kratzel, A., Steiner, S., Stalder, H. & Thiel, V. Coronavirus biology and replication: implications for SARS-CoV-2. *Nat. Rev. Micobiol.* **19,** 155–170 (2021).
12. York, A.G. et al. Instant super-resolution imaging in live cells and embryos via analog image processing. *Nat. Methods* **10,** 1122–1126 (2013).
