# Supplementary material for: A highly photostable and bright green fluorescent protein
Source: Nat Biotechnol. 2022 Apr 25;40(7):1132–42. doi: 10.1038/s41587-022-01278-2 (PMC9287174; doi:10.1038/s41587-022-01278-2)
Supplement: Supplementary file 2 — Reporting Summary [file 41587_2022_1278_MOESM2_ESM.pdf]

nature research

Corresponding author(s): Atsushi Miyawaki

Last updated by author(s): Mar 4, 2022

Reporting Summary

Nature Research wishes to improve the reproducibility of the work that we publish. This form provides structure for consistency and transparency in reporting. For further information on Nature Research policies, see our Editorial Policies and the Editorial Policy Checklist.

Statistics

For all statistical analyses, confirm that the following items are present in the figure legend, table legend, main text, or Methods section.

n/a	Confirmed	
☐	☒	The exact sample size (*n*) for each experimental group/condition, given as a discrete number and unit of measurement
☐	☒	A statement on whether measurements were taken from distinct samples or whether the same sample was measured repeatedly
☐	☒	The statistical test(s) used AND whether they are one- or two-sided<br>*Only common tests should be described solely by name; describe more complex techniques in the Methods section.*
☒	☐	A description of all covariates tested
☐	☒	A description of any assumptions or corrections, such as tests of normality and adjustment for multiple comparisons
☐	☒	A full description of the statistical parameters including central tendency (e.g. means) or other basic estimates (e.g. regression coefficient) AND variation (e.g. standard deviation) or associated estimates of uncertainty (e.g. confidence intervals)
☐	☒	For null hypothesis testing, the test statistic (e.g. *F*, *t*, *r*) with confidence intervals, effect sizes, degrees of freedom and *P* value noted<br>*Give P values as exact values whenever suitable.*
☒	☐	For Bayesian analysis, information on the choice of priors and Markov chain Monte Carlo settings
☒	☐	For hierarchical and complex designs, identification of the appropriate level for tests and full reporting of outcomes
☒	☐	Estimates of effect sizes (e.g. Cohen's *d*, Pearson's *r*), indicating how they were calculated

Our web collection on statistics for biologists contains articles on many of the points above.

Software and code

Policy information about availability of computer code

Data collection	ProteomeLab XL-A/XL-I (ver. 6.0, Beckman Coulter), AQUACOSMOS (ver. 2.63, Hamamatsu Photonics), cellSens (ver. 2.6 and ver. 3.3.1, Olympus), NIS-Elements AR (ver. 5.30.00, Nikon), Elyra 7(ZEN 3.0 SR FP2 (black) ver. 16.0.10.306, ZEISS), FV10i (ver. 2.1.1.7, Olympus), FV3000 (ver. 2.6.1.243, Olympus), MetaMorph (ver. 7.10.2.240, Molecular Devices).
Data analysis	Excel (2019), Origin Pro (ver. 2020b), BLAST (https://blast.ncbi.nlm.nih.gov/Blast.cgi?CMD=Web&PAGE_TYPE=BlastHome), ProteomeLab XL-A/XL-I (ver. 6.0, Beckman Coulter), EPSON Scan (ver. 3.24J), AQUACOSMOS (ver. 2.63, Hamamatsu Photonics), NIS-Elements AR (ver. 5.30.00), cellSens (ver. 2.6 and ver. 3.3.1), ImageJ (ver. 1.53h), MetaMorph (ver. 7.10.2.240, Molecular Devices), Biacore T200 Evaluation Software (ver. 2.0), TMPGEncPlus (ver. 2.5), Volocity (ver. 6.3.1, Quorum Technologies) and ZEN (ver. 9.1, 2014).<br>A customized program was generated based on C++ and OpenCV 3.4.1 (https://opencv.org) for ER network dynamics (Fig. 4, Extended Data Fig. 1b, Extended Data Fig.2b and Supplementary Fig. 11d, h, i). The source code of the program is included in the same R2DMS repository as the raw data (see the Data section blow).

For manuscripts utilizing custom algorithms or software that are central to the research but not yet described in published literature, software must be made available to editors and reviewers. We strongly encourage code deposition in a community repository (e.g. GitHub). See the Nature Research guidelines for submitting code & software for further information.

Data

Policy information about availability of data

All manuscripts must include a data availability statement. This statement should provide the following information, where applicable:

- Accession codes, unique identifiers, or web links for publicly available datasets
- A list of figures that have associated raw data
- A description of any restrictions on data availability

The accession numbers in the DDBJ/EMBL/GenBank databases are [LC593677] for CU17S, [LC601652] for StayGold, [LC593679] for h-StayGold, and [LC601653] for oxStayGold.
Deposited raw data at the R2DMS (RIKEN Research Data and copyrighted-work Management System) repository (https://dmsgrdm.riken.jp:5000/9gnxe/) are associated with the following items:
• Figures 1, 2, 3, 4 and 5
• Extended Data Figures 1, 3 and 4
• Supplementary Figures 2, 3, 4, 5, 6, 7, 8, 9, 11, 12, 13, 15, 16, 17 and 18
• Supplementary Videos 1, 2, 3, 4, 5 and 6

Field-specific reporting

Please select the one below that is the best fit for your research. If you are not sure, read the appropriate sections before making your selection.

☒ Life sciences ☐ Behavioural & social sciences ☐ Ecological, evolutionary & environmental sciences

For a reference copy of the document with all sections, see nature.com/documents/nr-reporting-summary-flat.pdf

Life sciences study design

All studies must disclose on these points even when the disclosure is negative.

Sample size

No explicit calculations were made to determine sample size. We empirically determined that ensured reproducibility. n = 4 different experiments (Figure 1m); n = 12 fields from three different experiments (Supplementary Fig. 7a); n = 3 different experiments (Supplementary Fig. 13b); n = 3 different experiments (Supplementary Fig. 3b).

Data exclusions

No data were excluded.

Replication

It always takes a great amount of time to follow the fluorescence from photostable FPs until they fade away substantially. So, we analyzed their photobleaching behavior from all angles (i.e., in vitro, in living cells in distinct media, in different organelles, by using different microscopy systems...) with as many different reference FPs as possible.

All imaging experiments were independently performed at least twice with similar results. In principle, representative images are shown with "multiple cells per field of view." An exception is seen in Supplementary Fig. 13b.
<Fig. 4a-d>
Three neighboring cells exhibited the same ER dynamics response to calcium mobilization.
<Fig. 4f-i>
Three neighboring cells exhibited the same ER dynamics response to calcium mobilization.
<Fig. 5e>
Representative of n = 9 independent samples.
<Figure 5f>
Representative of n = 2 independent infections.
<Figure 5g>
A single-layer 3D-SIM image of SARS-CoV-2 spike protein (green) and Nuclear (blue) extracted from volumetric image data shown in Extended Data Fig. 5. Representative of n = 5 cells over 3 independent infections.
<Figure 5h>
Representative of n = 2 areas over 2 independent infections.
<Figure 5i-k>
Representative of n = 3 imaging experiments from a single sample per staining condition (Supplementary Fig. 15).
<Extended Data Fig. 1>
Shown is a representative of n = 5 independent experiments that imaged rapid motion of ER tubules at the temporal resolution of > 100 frames/s.
<Extended Data Fig. 2a,b>
Shown is a representative of n = 9 independent transfections, including experiments #1, #2, #3, and #4 (Supplementary Fig. 11a-d).
<Extended Data Fig. 3>
Similar results were obtained from 2 other independent cultured cell samples.
<Extended Data Fig. 4a>
The EB3 dynamics shown are representative of n = 3 similar observations.
<Extended Data Fig. 4b>
Similar images were obtained from 6 other imaging experiments performed at DIV 21–25 on 4 independent neuronal samples.
<Extended Data Fig. 5>
The volumetric imaging experiment in z steps of 0.12 micrometer was repeated 4 times with 39–79 slices. In each case, the fluorescence in

the last frame (slice) was considerably strong for reliable super-resolution imaging.
<Supplementary Fig. 7b>
8 different passages of cells were used for each replicate.
<Supplementary Fig. 8>
Brightness of colony fluorescence was judged in single experiments (n = 1). The PM-targeting images are representative of 3 repetitions (n = 3 independent transfections).
<Supplementary Fig. 9>
The images are representative of 3 repetitions (n = 3 independent experiments).
<Supplementary Fig. 11a-d>
4 different passages of cells were used.
<Supplementary Fig. 11e-h>
4 different passages of cells were used.
<Supplementary Fig. 12b>
3 different passages of cells were used for each replicate.
<Supplementary Fig. 13b>
3 different passages of cells were used for each replicate.
<Supplementary Fig. 15>
The data shown are from single samples per staining condition.

All other experiments were independently performed at least twice with similar results.
<Supplementary Fig. 2a>
Representative of n = 2 independent experiments.
<Supplementary Fig. 2b>
Representative of n = 4 independent experiments.
<Supplementary Fig. 3>
Representative of n = 3 independent experiments.

Randomization: No allocation was performed in this study. All the cell samples observed were randomly selected.

Blinding: Blinding was not done because the data acquisition and analysis were conducted under the identical criteria/conditions/parameters in each comparison.

Reporting for specific materials, systems and methods

We require information from authors about some types of materials, experimental systems and methods used in many studies. Here, indicate whether each material, system or method listed is relevant to your study. If you are not sure if a list item applies to your research, read the appropriate section before selecting a response.

Materials & experimental systems

n/a	Involved in the study	
☐	☒	Antibodies
☐	☒	Eukaryotic cell lines
☒	☐	Palaeontology and archaeology
☐	☒	Animals and other organisms
☒	☐	Human research participants
☒	☐	Clinical data
☒	☐	Dual use research of concern

Methods

n/a	Involved in the study	
☒	☐	ChIP-seq
☒	☐	Flow cytometry
☒	☐	MRI-based neuroimaging

Antibodies

Antibodies used:
Rabbit Ab to ERGIC3 (Abcam, ab129179)
donkey Ab to rabbit IgG conjugated to Alexa Fluor 555 (Thermo Fisher Scientific, A-31572)
Mouse mAb to Nsp8 (GeneTex, GTX632696)
donkey Ab to mouse IgG conjugated to Alexa Fluor 555 (Thermo Fisher Scientific, A-31570)
Mouse mAb to dsRNA (Merck, MABE1134-100UL)
donkey Ab to mouse IgG conjugated to Alexa Fluor 555 (Thermo Fisher Scientific, A-31570)
Mouse mAb to pan-cadherin (Sigma-Aldrich, C1821)
Rabbit Ab to GM130 (MBL, PM061)
donkey Ab to mouse IgG conjugated to Alexa Fluor 647 (Thermo Fisher Scientific, A-31571)
Donkey Ab to rabbit IgG conjugated to Alexa Fluor 647 (Thermo Fisher Scientific, A-31573)

Validation:
Rabbit Ab to GM130 (MBL, PM061): validated by the manufacturer based on Western blotting and immuno-cytochemical data.
Relevant citations include Nat. Commun. 10 (1):603 (2019) and JBC 292 (10): 4089-4098 (2017).
Rabbit Ab to ERGIC3 (Abcam, ab129179): validated by the manufacturer based on Western blotting and immuno-cytochemical data.
A relevant citation is JBC 294:10900 - 10912 (2019).
Mouse mAb to Nsp8 (GeneTex, GTX632696): validated by the manufacturer based on Western blotting, flow cytometry, and

immuno-cytochemical data. Relevant citations include Sci. Rep. 11(1): 2229 (2021) and Cell Death Dis. 11(8): 656 (2020).
Mouse mAb to dsRNA (Merck, MABE1134-100UL): validated by the manufacturer based on immuno-cytochemical data. Relevant citations include Cell Rep. 15 (11): 2323 - 2330 (2016) and Cell Rep. 16(1): 232 - 246 (2016).
Mouse mAb to pan-cadherin (Sigma-Aldrich, C1821): validated by the manufacturer based on Western blotting and immuno-histo/cytochemical data. Relevant citations include Mol. Cell. Biol. 28(2): 666 - 677 (2007) and JBC 290 (21): 13427 - 13439 (2015).

Eukaryotic cell lines

Policy information about cell lines

Cell line source(s)	HeLa.S3 cells were obtained from ATCC (CCL-2.2).<br>COS-7 cells were obtained from ATCC (CRL-1651).<br>VeroE6/TMPRSS2 cells were purchased from Japanese Collection of Research Bioresources (JCRB) Cell Bank (JCRB1818).<br>SARS-CoV-2 KUH003 strain (DDBJ accession number LC630936) was isolated from a COVID-19 patient hospitalized at Kitasato University Hospital.
Authentication	The HeLa. S3 cell line was authenticated by STR profiling. The COS-7 and VeroE6/TMPRSS2 cell lines were not authenticated.
Mycoplasma contamination	Cell lines were not tested for mycoplasma contamination.
Commonly misidentified lines (See ICLAC register)	No commonly misidentified cell lines were used in this study.

Animals and other organisms

Policy information about studies involving animals; ARRIVE guidelines recommended for reporting animal research

Laboratory animals	This study did not involve laboratory animals.
Wild animals	This study did not involve wild animals.
Field-collected samples	Colonies of hydrozoan Cytaeis uchidae were obtained from the sea near Asamushi Marine Biological Station (Aomori Prefecture, Japan) and maintained in artificial seawater SEA LIFE (Marine Tech, Tokyo, Japan) at 20 °C in 12h/12h light/dark cycles.
Ethics oversight	No ethical approval was required because the jelly fish are not categorized as experimental animals in the Japanese laws.

Note that full information on the approval of the study protocol must also be provided in the manuscript.
